# Synthesis and evaluation of anticancer activity of quillaic acid derivatives: A cell cycle arrest and apoptosis inducer through NF-**κ**B and MAPK pathways

**DOI:** 10.3389/fchem.2022.951713

**Published:** 2022-09-07

**Authors:** Xing Huang, Chang-Hao Zhang, Hao Deng, Dan Wu, Hong-Yan Guo, Jung Joon Lee, Fen-Er Chen, Qing-Kun Shen, Li-Li Jin, Zhe-Shan Quan

**Affiliations:** Key Laboratory of Natural Medicines of the Changbai Mountain, Affifiliated Ministry of Education, College of Pharmacy, Yanbian University, Jilin, China

**Keywords:** quillaic acid, Western blot, antitumor, cell-cycle arrest, apoptosis

## Abstract

A series of quillaic acid derivatives with different substituents on the 28-carboxyl group were designed and synthesized. Five human cancer cell lines (HCT116, BEL7402, HepG2, SW620, and MCF-7) were evaluated for their antitumor activity *in vitro.* Some of the tested derivatives showed improved antiproliferative activity compared to the lead compound, quillaic acid. Among them, compound **E** (IC_50_ = 2.46 ± 0.44 μM) showed the strongest antiproliferative activity against HCT116 cells; compared with quillaic acid (IC_50_ > 10 μM), its efficacy against HCT116 cancer cells was approximately 4-fold higher than that of quillaic acid. Compound **E** also induces cell cycle arrest and apoptosis by modulating NF-κB and MAPK pathways. Therefore, the development of compound **E** is certainly valuable for anti-tumor applications.

## 1 Introduction

Malignant tumors, also known as cancers, have become a disease that threatens human health (Bray et al., 2018). Drug therapy remains an effective and important method for the clinical treatment of cancer. One of the main obstacles to the pharmacological properties of compounds with effective anti-tumor activity is their toxicity to normal cells. Therefore, it is of great importance to develop new anti-cancer drugs that are less toxic to normal cells (Shen et al., 2019).

In recent years, the discovery of natural plant-derived antitumor drugs has attracted significant attention from medicinal chemists. All approved therapeutic drugs from 1981 to 2019 (39 years) and corresponding diseases from 1946 to 2019, a total of 1,881 drugs were reported. Among them, 71 (3.8%) were natural products, 14 (0.8%) were natural products’ “Botanical” (in general these have been approved), and 356 (18.9%) were from semi-synthetic modifications of natural products (Newman and Cragg 2020). Natural products remain the best source of effective drugs for treating human diseases.

**FIGURE 1:**
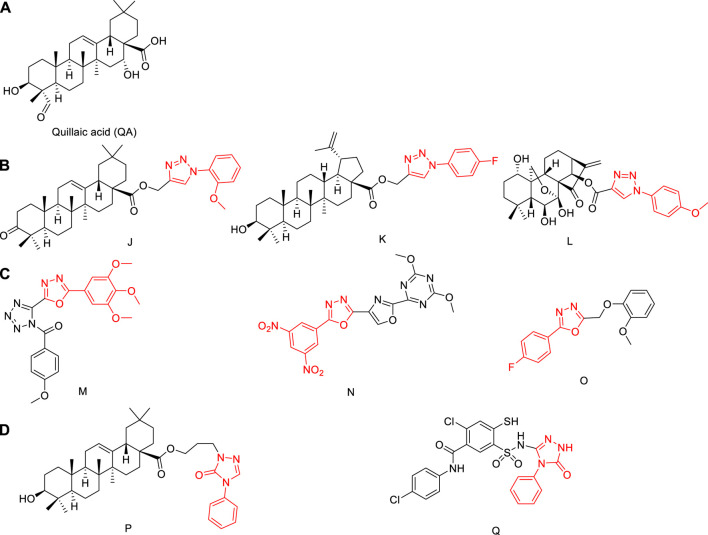
**(A)** Structures of quillaic acid. **(B)** Structures of natural-product derivatives containing phenyl-1,2,3-triazole; **(C)** Structures of compounds containing phenyl-1,3,4-oxadiazole; **(D)** Structures of compounds containing 1,2,4-triazol-3-one.

*Saponaria officinalis L.* (Caryophyllaceae), commonly known as soap weed, is native to Europe and Asia, and is grown as a horticultural plant worldwide. Quillaic acid (Figure 1A), which is isolated from this plant, is a pentacyclic triterpenoid (Lu et al., 2015).

Quillaic acid has a variety of biological activities, including anti-inflammatory (Rodriguez-Diaz et al., 2011), immune-stimulatory (Moses et al., 2014), anti-viral (Boke Sarikahya et al., 2021), and cytotoxic (Guzman et al., 2020). Quillaic acid is a well-known cytotoxic triterpenoid that has demonstrated apoptosis-inducing ability in diverse cells (Yazici et al., 2021).

Phenyl-1,2,3-triazole has a high dipole moment and can form hydrogen bonds with drug targets, which is conducive to the binding of the compound to the target (Bock et al., 2007), Triazole is an important heterocyclic structural unit, used in anticonvulsant effect (Liu et al., 2017; Huang et al., 2018; Shao et al., 2018; Zhang G. R. et al., 2018), antibacterial (Yan et al., 2021; Zhang et al., 2021), anti-inflammatory (Pang et al., 2020; Ma et al., 2022; Zhang et al., 2022), anti-cancer (Liu et al., 2021), and anti-toxoplasmic (Zhang H. B. et al., 2018; Shang et al., 2021) applications, among others.

A biological allele of amides and esters, 1,3,4-oxadiazole can affect the pharmacokinetic properties of a drug by increasing its lipophilicity, thereby improving the ability of the drug to diffuse across a membrane (Bajaj et al., 2015). One of four isomers of oxadiazole, 1,3,4-oxadiazole also has antibacterial (Wang et al., 2021), antitumor (Zhao et al., 2021), and other biological properties.

Fragments of phenyl-1,2,3-triazole or phenyl-1,3,4-oxadiazole molecules are sometimes combined with natural products to improve their anti-tumor activities (Chi et al., 2017; Shen et al., 2019). The synthetic strategies of compounds **J**, **K**, **L**, **M**, **N**, **and O** in Figure 1B and Figure 1C have attracted increasing attention (Rashid et al., 2013; Khan et al., 2016; Lakshmithendral et al., 2019; Shen et al., 2019; Kotla et al., 2020; Balaraju et al., 2021). Among them, compounds **J** and **K** were modified at the carboxyl position and linked to phenyl-1,2,3-triazole with an ester bond, and the antitumor activity was significantly enhanced. Compound **L** is also linked by an ester bond, and also obtains better antitumor activity. Therefore, combining a phenyl-1,2,3-triazole group or phenyl-1,3,4-oxadiazole with quillaic acid through an ester bond is an effective strategy to enhance the antitumor activity of quillaic acid.

3*H*-1,2,4-triazol-3-one also has a wide range of applications in anti-fungal (Sheng et al., 2011) and anti-inflammatory (Wei et al., 2018) fields. Compounds **P** and **Q** in Figure 1D showed significant anti-tumor activity (Chi et al., 2017; Zhang H. J. et al., 2018). Therefore, combining 3*H*-1,2,4-triazol-3-one with quillaic acid through an ester bond enhanced the antitumor activity of quillaic acid.

In this study, quillaic acid was selected as the lead compound, and phenyl-1,2,3-triazole, phenyl-1,3,4-oxadiazole, and 3*H*-1,2,4-triazol-3-one were introduced into the C-28 carboxyl group of quillaic acid. Thereby, we designed and synthesized different phenyl-1,2,3-triazoles (**A1–10**), phenyl-1,3,4-oxadiazoles (**B1–B6**), 3*H*-1,2,4-triazol-3-ones (**C1–C6**).

**FIGURE 2:**
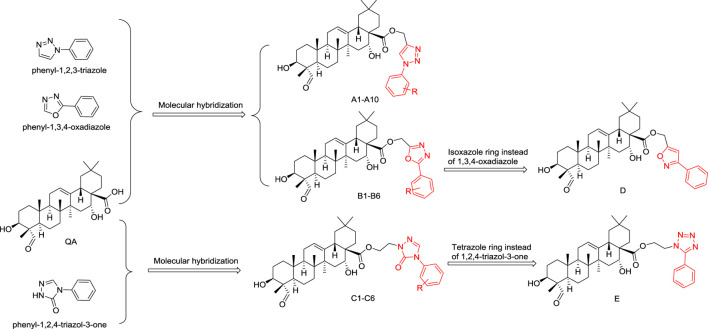
Designing quillaic-acid derivatives containing a nitrogen-containing heterocycle to achieve potential anti-proliferative activity.

Compounds **D** and **E** were designed and synthesized according to an alternative design with an isoxazole or tetrazole ring because of their similar physicochemical and biological properties to the 1,3,4-oxadiazole and 1,2,4-triazole-3-one rings, respectively (Figure 2). Subsequently, all 24 new compounds were screened against five different cancer cell lines. The toxicity of the compounds in normal human liver L02 cells was also tested.

## 2 Results and discussion

### 2.1 Chemistry

**SCHEME 1:**
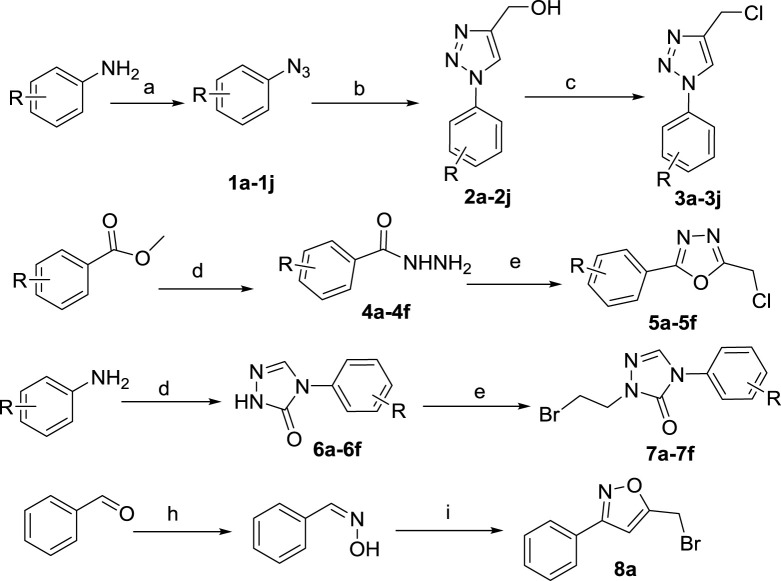
Reagents and conditions: **(A)** (i) HCl, NaNO_2_, H_2_O, 0–5°C, 30 min; (ii) NaN_3_, H_2_O, 0–5°C, 2–4 h; **(B)** propynol, Na ascorbate, CuSO_4_•5H_2_O, H_2_O, t-BuOH/H_2_O, 25°C, overnight; **(C)** thionyl chloride, CH_2_Cl_2_, r. t., overnight; **(D)** NH_2_-NH_2_•H_2_O, EtOH, reflux, overnight; **(E)** chloroacetic acid, POCl_3_, reflux, 4 h; **(F) (I)** methyl hydrazinocarboxylate, triethyl orthoformate, EtOH, reflux, 48 h; (ii) NaOCH_3_, EtOH, reflux, 48 h; **(G)** 1,2-dibromoethane, DMF, K_2_CO_3_, 60°C, 4–6 h; **(H)** NH_2_-OH•HCl, K_2_CO_3_, CH_3_OH:H_2_O = 1:1, r. t., overnight; **(I)** NaClO, CH_2_Cl_2_, H_2_O, 0°C, 8 h.

The synthesis of the intermediates is shown in Scheme 1. Compounds **1a–1j** were obtained by diazotization and azide reactions of various anilines (Zhao et al., 2019). Then, a click reaction between intermediates **1a–1j** and propynol gave compounds **2a–2j** (Luan et al., 2019). Compounds **2a–2j** were mixed with thionyl chloride in CH_2_Cl_2_ at room temperature to give compounds **3a–3j** (Ghosh et al., 2012).

The substituted methyl benzoate reacted with hydrazine hydrate to obtain **4a–4f**, which were then closed with chloroacetic acid under the action of phosphorus oxychloride to obtain **5a–5f** (Sheng et al., 2011). Aniline reacted with methyl carbazate and triethyl orthoformate; it was then catalyzed by sodium methoxide to obtain **6a-6f**, which reacted with dibromoethane to obtain the intermediates **7a-7f** (Wei et al., 2017). Benzaldehyde reacts with hydroxylamine hydrochloride and then reacts with sodium hypochlorite solution to obtain **8a** (Liu et al., 2010).

**SCHEME 2:**
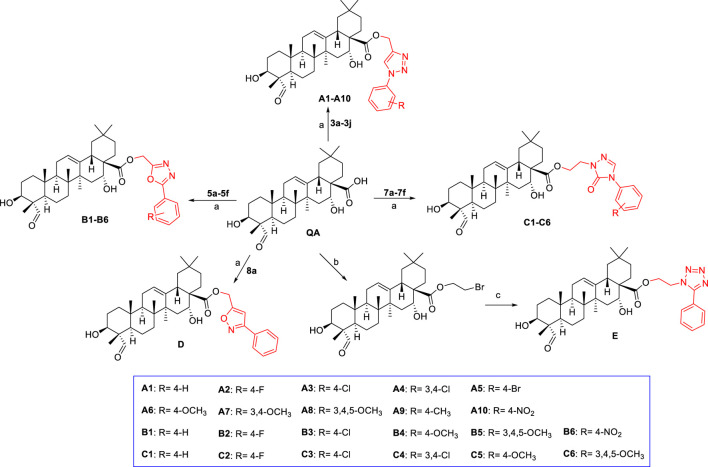
Reagents and conditions: **(A)** DMF, K_2_CO_3_, 60°C, 4–6 h; **(B)** 1,2-dibromoethane, DMF, K_2_CO_3_, KI, 60°C, 4–6 h; **(C)** 5-phenyl-1*H*-tetrazole, DMF, K_2_CO_3_, KI, 60°C, 8 h.

The general path to synthesize the target quillaic acid analogues **A1–A10**, **B1–B6**, **C1–C6**, **D**, and **E** is shown in Scheme 2. Quillaic acid was used as the raw material, and K_2_CO_3_ as the catalyst, to react with different intermediates (**3a-3j**, **5a-5f**, **7a-7f**, **8a**) in anhydrous DMF at 60°C to obtain the target compounds **A1–A10**, **B1–B6**, **C1–C6**, and **D** (Chi et al., 2017; Wei et al., 2018). Quillaic acid first reacted with dibromoethane and then reacted with 5-phenyl-1*H*-tetrazole with K_2_CO_3_, a KI catalyst, at 60°C in anhydrous DMF to obtain the target compound **E** (Zhang et al., 2019). All synthesized compounds were characterized by ^1^H-NMR, ^13^C-NMR, and high-resolution mass spectrometry.

### 2.2 Biological evaluation

#### 2.2.1 *In vitro* anti-proliferative activity and SAR study

The anti-proliferative activities against five human cancer cell lines, including HCT116 (human colorectal cancer cell line), MCF-7 (human breast cancer cell line), SW620 (human colorectal cancer cell line), BEL7402 (human hepatocellular carcinoma cell line), and HepG2 (human hepatocellular carcinoma cell line) were established for all the target compounds using the MTT assay. The IC_50_ values (concentration required to inhibit tumor cell proliferation by 50%) are listed in Table 1.

**TABLE 1:** Antiproliferative efficacy of quillaic acid derivatives of compounds **A1- A10**, **B1-B6**, **C1-C6**, **D**, and **E** in three human cancer cell lines^a^.

Compound	IC_50_ values (μM)^b^				
	HCT116	SW620	BEL7402	HepG2	MCF-7
**QA**	>10	>10	>10	>10	>10
**A1**	3.61 ± 1.01	8.96 ± 1.78	>10	8.28 ± 2.10	>10
**A2**	3.04 ± 0.92	8.78 ± 1.77	3.18 ± 1.02	>10	>10
**A3**	>10	>10	>10	>10	>10
**A4**	>10	>10	>10	>10	>10
**A5**	>10	>10	>10	>10	>10
**A6**	>10	>10	>10	>10	>10
**A7**	>10	>10	>10	>10	>10
**A8**	>10	>10	>10	>10	>10
**A9**	>10	>10	>10	>10	>10
**A10**	>10	>10	>10	>10	>10
**B1**	>10	>10	>10	>10	>10
**B2**	3.12 ± 0.87	7.75 ± 1.43	2.84 ± 0.66	4.59 ± 1.73	3.99 ± 1.28
**B3**	5.01 ± 1.12	8.18 ± 1.85	>10	>10	>10
**B4**	3.65 ± 1.06	7.23 ± 1.56	9.15 ± 2.21	8.38 ± 2.22	>10
**B5**	3.81 ± 1.23	>10	7.36 ± 1.45	8.45 ± 2.15	>10
**B6**	3.65 ± 1.22	>10	>10	>10	>10
**C1**	4.43 ± 1.34	6.12 ± 1.56	>10	>10	>10
**C2**	>10	>10	>10	>10	>10
**C3**	3.11 ± 1.02	7.11 ± 1.54	>10	>10	>10
**C4**	>10	>10	>10	>10	>10
**C5**	3.21 ± 1.07	7.25 ± 1.72	>10	>10	>10
**C6**	5.60 ± 1.55	7.35 ± 1.70	>10	>10	>10
**D**	>10	>10	>10	>10	>10
**E**	2.46 ± 0.44	4.69 ± 1.10	6.89 ± 1.69	8.89 ± 2.24	7.59 ± 1.78

a MTT, methods: cells were incubated with indicated compounds for 48 h (means ± SD, *n* = 3).

b IC_50_: concentration that inhibits 50% of cell growth.

The IC_50_ of quillaic acid against five types of cancer cells was greater than 10 μM. Quillaic acid derivatives **A1** and **A2** containing different phenyl 1,2,3-triazoles exhibited stronger anti-proliferative activities against all five selected cancer cell lines than quillaic acid. Among them, compound **A2** with 4-fluorophenyl 1,2,3-triazole was the most potent compound in the series, having an IC_50_ value of 3.04 μM in the HCT116 cell line.

Compounds **B2–B5**, with different phenyl-1,3,4-oxadiazoles, were slightly more potent against HCT116, SW620, MCF-7, HepG2, and BEL7402 cells than quillaic acid. Among them, compound **B2** (with 4-fluorophenyl-1,2,3-triazole) was the most potent compound in this series with an IC_50_ value of 3.12 μM against the HCT116 cell line and 2.84 μM against the BEL7402 cell line. In order to explore the anti-proliferative activity of the phenyl-1,2,3-triazole group, we designed and synthesized compound **D** by replacing it with isoxazole. Unfortunately, compound **D** had no strong activity against the five types of cancer cells. The order of activity was 4-F > 4-NO_2_ > 3,4,5-OCH_3_ > 4-OCH_3_ > 4-Cl.

Compounds **C1**, **C3**, **C5**, and **C6**, with different phenyl-1,2,4-triazol-3-ones, were slightly more potent against HCT116 and SW620 cells than quillaic acid. Compound **C3** (with 4-chlorophenyl-1,2,4-triazol-3-one) was the most potent compound in this series with an IC_50_ value of 3.11 μM against the HCT116 cell line. The order of activity was 4-Cl > 4-OCH_3_ > 4-H > 3,4,5-OCH_3_. In order to explore the importance of the phenyl-1,2,4-triazol-3-one group for anti-proliferative activity, we designed and synthesized compound **E** by replacing it with tetrazole. Fortunately, compound **E** had strong activity against five types of cancer cells. Compound **E** had the strongest activity against HCT116 cells, with an IC_50_ of 2.46 μM, followed by SW620 with an IC_50_ of 4.69 μM.

#### 2.2.2 Selective inhibition of cancer-cell growth by compounds **B2**, and **E** *in vitro*

The lack of selectivity between normal and cancer cells is one of the main limitations of antitumor drugs (Dy and Adjei, 2013). Therefore, we evaluated the cytotoxicity of compounds **B2**, and **E** in normal cell lines L02 (human normal liver cells) to determine the selectivity index (ratio of cytotoxicity in L02 cells compared to that in cancer cells). As shown in Table 2, compound **E** exhibited a 4.06-fold higher selectivity for HCT116 cells than for normal L02 cells; compound **E** not only exhibited the strongest anti-proliferative activity against HCT116 cells, but selectively inhibited tumor cells. Therefore, this compound was chosen for further biological studies.

**TABLE 2:** *In vitro* cytotoxic of compounds **B2** and **F** against normal cell line (L02)^a^.

Comp	L02 (IC_50,_ μM)	Selectivity index^b^				
		HCT116	SW620	BEL7402	HepG2	MCF-7
**B2**	>10	3.20	1.29	3.52	2.18	2.51
**E**	>10	4.06	2.13	1.45	1.12	1.32

a IC_50_: concentration that inhibits 50% of cell growth.

b SI: selective index (IC_50_ on normal cells/IC_50_ on tumour cells).

#### 2.2.3 Compound **E** inhibited HCT116 cell colony formation

**FIGURE 3:**
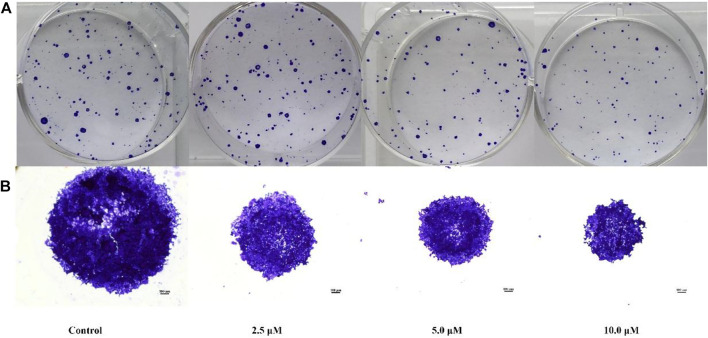
Colony formation of HCT116 cells inhibited by compound **E**. **(A)** HCT116 cells were incubated with varying concentrations of **E** (0, 2.5, 5, 10 μM) and stained with crystal violet. **(B)** The micrographic differences between the colonies. Images were taken of stained single colonies observed under a microscope.

The colony-forming cell assay was used to determine the ability of cells to proliferate and differentiate into colonies and thereby to investigate the anti-proliferative efficacy of compound **E** (Lei et al., 2015). As shown in Figure 3, exposure of HCT116 cells to compound **E** significantly decreased the number and size of colonies in a concentration-dependent manner.

#### 2.2.4 Compound **E** induced cell-cycle arrest

**FIGURE 4:**
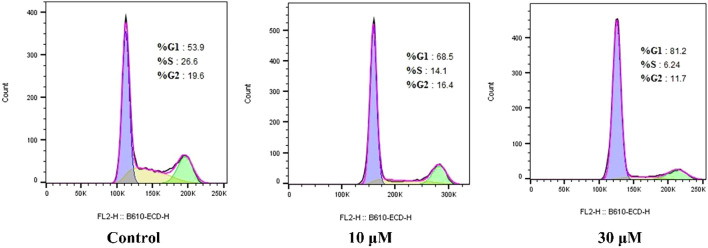
Compound **E** induced cell cycle arrest at G1 phase in HCT116.

To determine the effect of compound **E** on cell-cycle progression, flow cytometry analysis was performed on HCT116 cells treated with different concentrations (10 and 30 µM) of compound **E**. A dose-dependent G1 phase-transition arrest was observed in HCT116 cells treated with **E**, as shown in Figure 4. Treatment of HCT116 cells with compound **E** at a concentration of 30 μM resulted in 81.2% of cells arrested in the G1 phase, compared with 53.9% in the untreated group.

#### 2.2.5 Compound **E** induced morphological changes in HCT116 cells by haematoxylin and eosin staining

**FIGURE 5:**
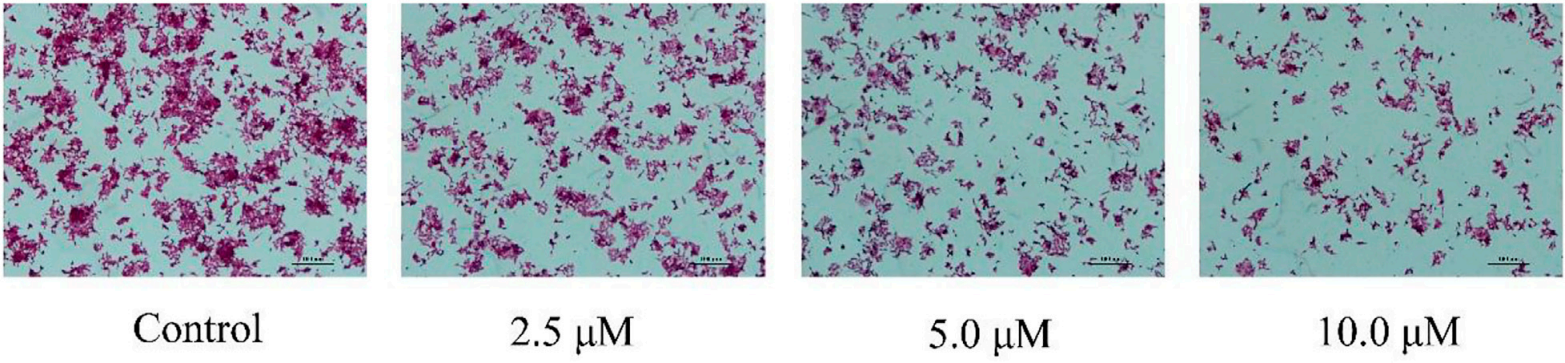
Cell morphological alterations and nuclear changes associated with HCT116 cells after incubation with varying concentrations **E** (0, 2.5, 5, 10 μM) for 24 h were assessed by staining with H&E and visualized by microscopy.

Induction of apoptosis is the mechanism of action of many anti-proliferative drugs. When cells undergo apoptosis, they generally also undergo morphological changes (Liu et al., 2009). Therefore, we used H&E staining to assess morphological changes in the cells. As shown in Figure 5, HCT116 cells incubated with **E** for 24 h displayed significant apoptotic morphological changes.

#### 2.2.6 Compound **E** induced cancer-cell apoptosis

**FIGURE 6:**
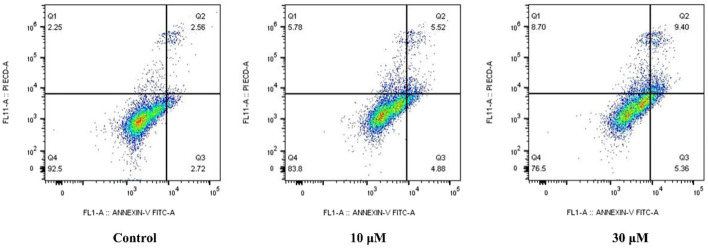
Compound **E** induced cell apoptosis in a concentration-dependent manner.

To confirm that **E** induces apoptosis in HCT116 cells, HCT116 cells were treated with different concentrations (10 and 30 µM) of compound **E**. After treatment with compound **E**, the apoptosis rate of HCT116 cells increased from 5.28% (control) to 14.76%, as shown in Figure 6. This result indicated that compound **E** induced apoptosis.

#### 2.2.7 Western-blot analysis of the expression of Bax, and Bcl-2

**FIGURE 7:**
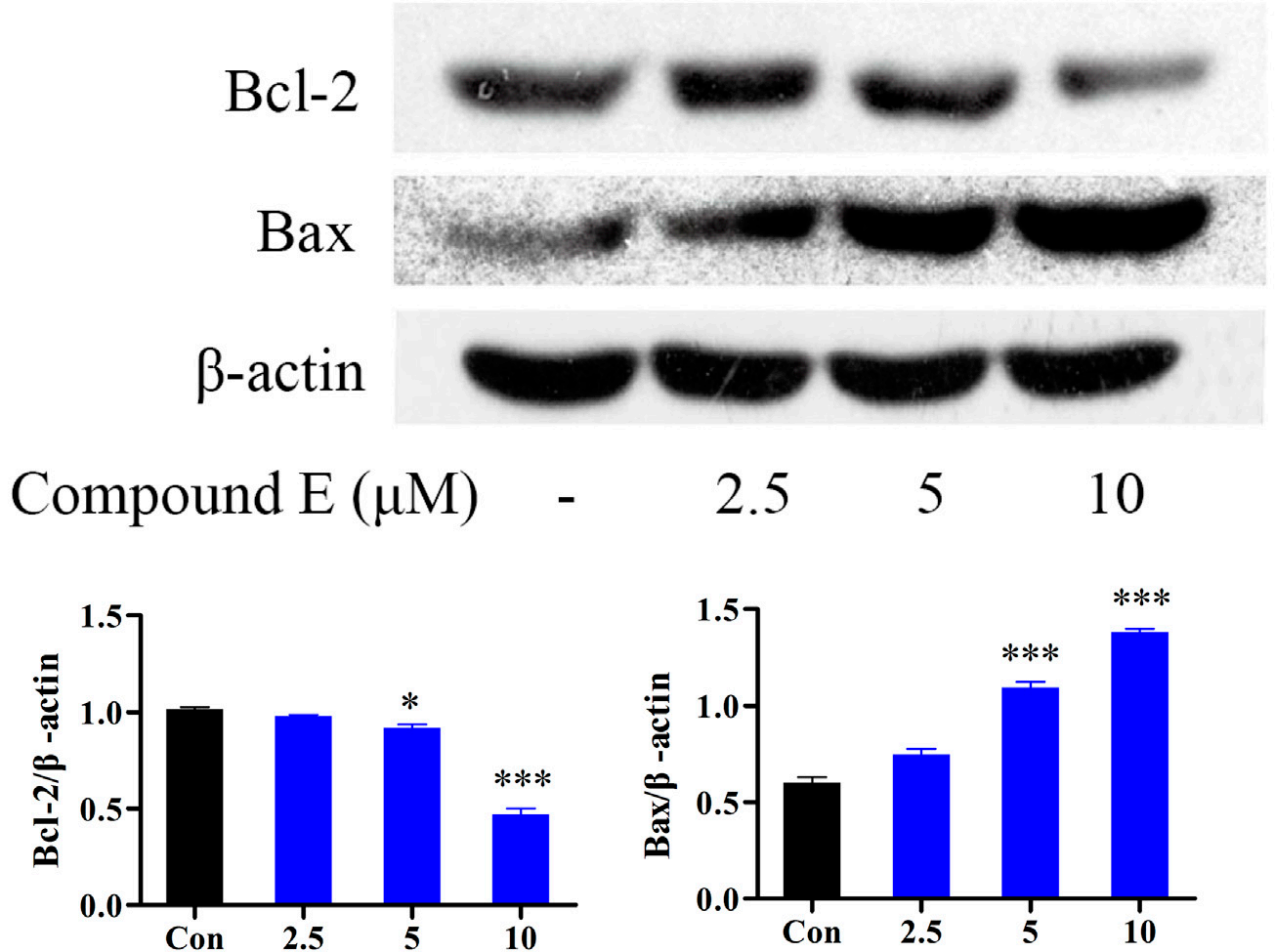
Effect of compound **E** on the levels of Bax, and Bcl-2 in HCT116 cells. Cells were pretreated with the various concentrations of compound **E** (2.5, 5 and 10 μM) for 24 h. The expression of Bax, and Bcl-2 were analyzed by Western blot. ***p < 0.001, **p < 0.01, *p < 0.05 vs. control group. The error bars represent the mean ± SD for three independent experiments.

The Bcl-2 protein family (including the anti-apoptotic protein Bcl-2 and the pro-apoptotic protein Bax) is a group of key apoptotic proteins that play an important role in the regulation of apoptosis (Han et al., 2013; Zhang and Saghatelian, 2013; Zheng et al., 2021). HCT116 cells were treated with different concentrations (2.5, 5, and 10 μM) of **E** to detect the expression of Bax and Bcl-2 proteins. The addition of 10 µM of compound **E** significantly increased the expression of Bax and decreased the level of Bcl-2, as shown in Figure 7.

#### 2.2.8 Effect of compound **E** on NF-κB signaling pathway in HCT116 cells

**FIGURE 8:**
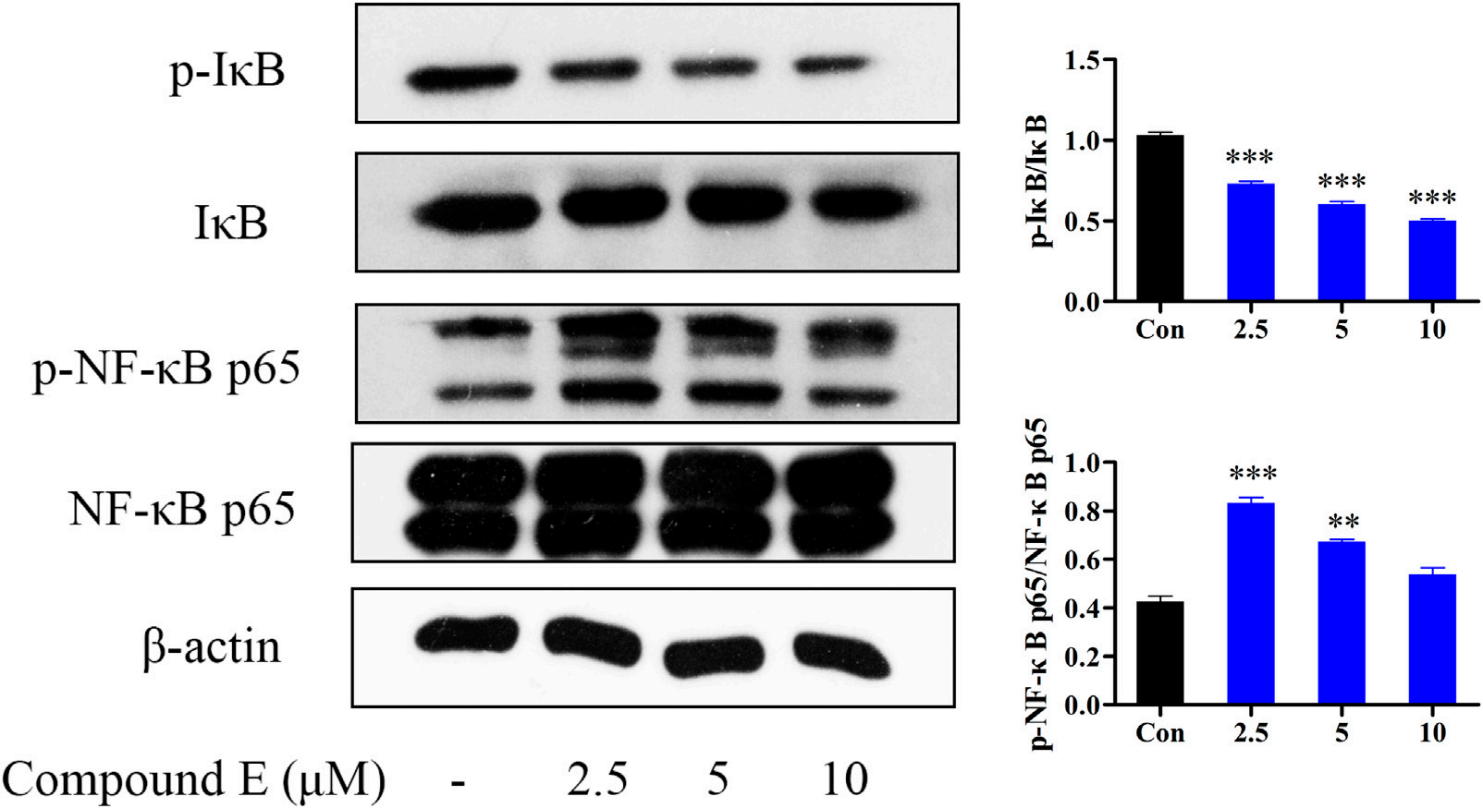
Effect of compound **E** on NF-κB signaling pathway in HCT116 cells. Cells were pretreated with the various concentrations of compound **E** (2.5, 5 and 10 μM) for 24 h. The expression of phospho-NF-κB p65, NF-κB p65, phospho-IκB, IκB were analyzed by Western blot. ***p < 0.001, **p < 0.01, *p < 0.05 vs. control group. The error bars represent the mean ± SD for three independent experiments.

The NF-κB pathway plays an important role in a variety of cellular functions, including cell growth, apoptosis, and tumorigenesis (Hwang et al., 2012). Interfering with NF-κB signaling can inhibit tumor cell proliferation and metastasis (Yenmis et al., 2021). Therefore, NF-κB inhibitors are considered as potential anticancer drugs (Wang et al., 2017). The results showed that compound **E** inhibited the phosphorylation and degradation of IκB (Figure 8), but had no inhibitory effect on NF-κB p65.

#### 2.2.9 Effect of compound **E** on MAPK signaling pathway in HCT116 cells

**FIGURE 9:**
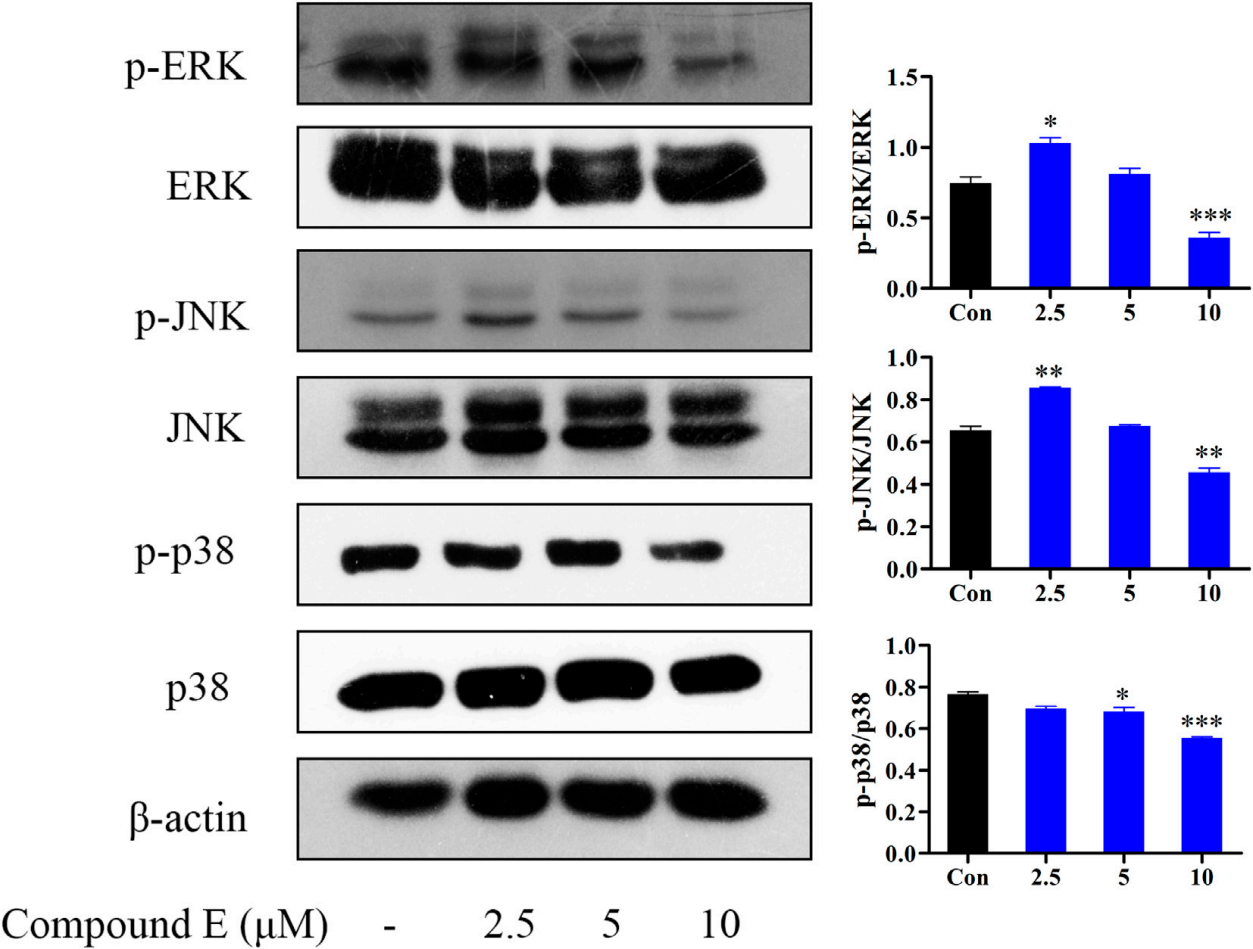
Effect of compound **E** on NF-κB signaling pathway in HCT116 cells. Cells were pretreated with the various concentrations of compound **E** (2.5, 5 and 10 μM) for 24 h. The expression of phospho-ERK, ERK, phospho-JNK, JNK, phospho-p38, p38 were analyzed by Western blot. ***p < 0.001, **p < 0.01, *p < 0.05 vs. control group. The error bars represent the mean ± SD for three independent experiments.

Extracellular signal-regulated kinases are the most closely related MAPK. MAPK, a family of serine/threonine protein kinases, is also an important transmitter of signaling from the cell surface to the nucleus, and it is involved in the regulation of many physiological processes, including apoptosis (Bian et al., 2021). The MAPK pathway has been implicated in regulating tumor angiogenesis, proliferation, metastasis and invasion (Ko et al., 2017). MAPK family is mainly composed of ERK, p38 and JNK subfamily. The results showed that compound **E** down-regulated the phosphorylation levels of ERK, JNK and p38 at 10 μM (Figure 9).

## 3 Conclusion

In summary, a new type of saponin derivative was designed, synthesized, and tested for its antitumor activity. The *in vitro* cell-growth inhibition test showed that compound **E** had the highest anti-proliferative activity against HCT116 cells (IC_50_ = 2.46 μM). The results of flow cytometry showed that compound **E** induced cell apoptosis and blocked cells in the G1 phase. Compound **E** induced HCT116 cell apoptosis by increasing the expression of Bax and decreasing the level of Bcl-2. Compound **E** also exerted its antitumor activity through NF-κB signaling pathway and MAPK signaling pathway. Therefore, compound **E** may be a potential anti-cancer drug and has value for further research.

## 4 Experimental section

### 4.1 Chemistry

All reagents and solvents were purchased from commercial sources and used as received without further purification. Reactions were monitored by thin-layer chromatography (TLC) in silica gel, and the TLC plates were visualised by exposure to ultraviolet light (254 and 365 nm). Compounds were purified using flash column chromatography over silica gel (200–300 mesh). Melting points were determined in open capillary tubes and are uncorrected. ^1^H-NMR and ^13^C-NMR spectra were measured on Bruker AV-300 and AV-500 spectrometer, with TMS as internal standard. Mass spectra were obtained on an HP1100LC (Agilent Technologies, USA).

### 4.2 General procedure for synthesis of compounds **A1-A10, B1-B6, C1-C6**, and **D**

A 25 ml round bottomed flask was charged with quillaic acid (50 mg, 0.10 mmol), intermediates **3a-3j**, **5a-5f**, **7a-7f**, and **8a** (0.12 mmol), K_2_CO_3_ (41.4 mg, 0.30 mmol) and DMF (5 ml). This reaction mixture was stirred vigorously at 60°C for 6 h and poured into the crushed ice. The mixture was extracted with ethyl acetate and the organic phase was washed with saturated sodium bicarbonate solution and brine, and dried over Na_2_SO_4_. The evaporation of the solvents gave the crude products, which were purified by silica gel column to afford compounds **A1-A10, B1-B6, C1-C6**, and **D.**

#### 4.2.1 (1-phenyl-1H-1,2,3-triazol-4-yl)methyl (4aR,5R,6aS,6bR,8aR,9S,10S,12aR,12bR,14bS)-9-formyl-5,10-dihydroxy-2,2,6a,6b,9,12a-hexamethyl-1,3,4,5,6,6a,6b,7,8,8a,9,10,11,12,12a,12b,13,14b-octadecahydropicene-4a (2H)-carboxylate (**A1**)

White solid; Yield: 79%; mp: 186–188°C; Purity: 95.72%; ^1^H-NMR (300 MHz, CDCl_3_) δ: 9.40 (s, 1H, -CHO), 8.02 (s, 1H, triazole-H), 7.75–7.71 (m, 2H, Ar-H), 7.58–7.53 (m, 2H, Ar-H), 7.50–7.45 (m, 1H, Ar-H), 5.40 (s, 1H, C_12_-H), 5.26 (dd, *J*_*1*_ = 18 Hz, *J*_*2*_ = 12 Hz, 2H, -COO-CH_2_-), 4.58 (s, 1H, C_16_-H), 3.78 (d, *J* = 9 Hz, 1H, C_3_-H), 3.08 (dd, *J*_*1*_ = 15 Hz, *J*_*2*_ = 6 Hz, 1H, C_18_-H), 2.18 (t, *J* = 15 Hz, 1H), 1.94–1.84 (m, 3H), 1.78 (d, *J* = 6 Hz, 3H), 1.73–1.56 (m, 7H), 1.49–1.26 (m, 9H), 1.24–1.10 (m, 3H), 1.04 (s, 3H), 0.98 (s, 3H), 0.92 (s, 3H), 0.83 (s, 3H), 0.46 (s, 3H). ^13^C-NMR (75 MHz, CDCl_3_) δ: 207.09, 176.71, 142.81, 136.85, 129.81, 128.96, 122.63, 122.49, 120.48, 74.66, 71.82, 57.64, 55.21, 48.65, 48.16, 46.53, 46.38, 41.39, 40.68, 39.76, 38.07, 35.92, 35.47, 32.78, 32.26, 31.01, 30.43, 26.87, 26.12, 24.52, 23.21, 20.66, 16.78, 15.56, 8.94. ESI-HRMS calcd for C_39_H_54_N_3_O_5_^+^ ([M + H]^+^): 644.40580; found: 644.40446.

#### 4.2.2 (1-(4-fluorophenyl)-1H-1,2,3-triazol-4-yl)methyl (4aR,5R,6aS,6bR,8aR,9S,10S,12aR,12bR,14bS)-9-formyl-5,10-dihydroxy-2,2,6a,6b,9,12a-hexamethyl-1,3,4,5,6,6a,6b,7,8,8a,9,10,11,12,12a,12b,13,14b-octadecahydropicene-4a (2H)-carboxylate (**A2**)

White solid; Yield: 67%; mp: 171–172°C; Purity: 94.65%; ^1^H-NMR (300 MHz, CDCl_3_) δ: 9.40 (s, 1H, -CHO), 7.98 (s, 1H, triazole-H), 7.73–7.69 (m, 2H, Ar-H), 7.24 (d, *J* = 9 Hz, 2H, Ar-H), 5.39 (s, 1H, C_12_-H), 5.24 (t, *J* = 15 Hz, 2H, -COO-CH_2_-), 4.57 (s, 1H, C_16_-H), 3.78 (d, *J* = 9 Hz, 1H, C_3_-H), 3.06 (d, *J* = 12 Hz, 1H, C_18_-H), 2.18 (t, *J* = 15 Hz, 1H), 1.87 (t, *J* = 15 Hz, 3H), 1.77 (s, 1H), 1.66 (s, 6H), 1.49 (t, *J* = 12 Hz, 2H), 1.36 (s, 3H), 1.28 (s, 2H), 1.20–1.14 (m, 3H), 1.11 (t, *J* = 3 Hz, 1H), 1.05 (s, 3H), 0.97 (s, 3H), 0.91 (s, 3H), 0.89 (s, 1H), 0.87 (s, 2H), 0.85 (s, 3H), 0.46 (s, 3H). ^13^C-NMR (75 MHz, CDCl_3_) δ: 207.50, 176.09, 162.09 (d, *J* = 244.5 Hz), 143.74, 143.47, 133.50, 123.90, 122.66 (d, *J* = 9.0 Hz), 121.91, 117.14 (d, *J* = 23.25 Hz), 86.44, 73.09, 57.23, 55.51, 48.20, 46.69, 41.37, 37.51, 35.68, 35.40, 34.96, 33.25, 31.66, 30.63, 26.81, 24.58, 16.71, 15.51, 15.12, 13.72, 9.24. ESI-HRMS calcd for C_39_H_52_N_3_O_5_F^+^ ([M + H]^+^): 662.39638; found: 662.39465.

#### 4.2.3 (1-(4-chlorophenyl)-1H-1,2,3-triazol-4-yl)methyl (4aR,5R,6aS,6bR,8aR,9S,10S,12aR,12bR,14bS)-9-formyl-5,10-dihydroxy-2,2,6a,6b,9,12a-hexamethyl-1,3,4,5,6,6a,6b,7,8,8a,9,10,11,12,12a,12b,13,14b-octadecahydropicene-4a (2H)-carboxylate (**A3**)

White solid; Yield: 67%; mp: 193–194°C; ^1^H-NMR (300 MHz, DMSO-*d6*) δ: 9.20 (s, 1H, -CHO), 8.81 (s, 1H, triazole-H), 7.95 (d, *J* = 9 Hz, 2H, Ar-H), 7.67 (d, *J* = 9 Hz, 2H, Ar-H), 5.17 (d, *J* = 9 Hz, 3H), 4.85 (s, 1H), 4.63 (s, 1H), 4.34 (s, 1H), 3.65 (s, 1H), 2.91 (d, *J* = 18 Hz, 1H), 2.21 (t, *J* = 12 Hz, 1H), 2.04 (d, *J* = 6 Hz, 1H), 1.85 (t, *J* = 15 Hz, 3H), 1.66–1.51 (m, 6H), 1.40 (t, *J* = 12 Hz, 3H), 1.28 (s, 3H), 1.19–1.06 (m, 5H), 0.98 (s, 2H), 0.90 (s, 4H), 0.83 (s, 3H), 0.65 (s, 3H), 0.21 (s, 3H). ^13^C-NMR (75 MHz, DMSO-*d6*)) δ: 207.50, 176.08, 143.74, 143.60, 135.77, 133.43, 130.28, 123.85, 122.00, 73.09, 70.84, 57.13, 55.51, 48.17, 46.69, 46.30, 41.35, 35.67, 35.41, 34.91, 33.25, 30.62, 26.80, 24.57, 23.24, 20.51, 16.70, 15.49, 9.23. ESI-HRMS calcd for C_39_H_53_N_3_O_5_Cl^+^ ([M + H]^+^): 678.36683; found: 678.36523.

#### 4.2.4 (1-(3,4-dichlorophenyl)-1H-1,2,3-triazol-4-yl)methyl (4aR,5R,6aS,6bR,8aR,9S,10S,12aR,12bR,14bS)-9-formyl-5,10-dihydroxy-2,2,6a,6b,9,12a-hexamethyl-1,3,4,5,6,6a,6b,7,8,8a,9,10,11,12,12a,12b,13,14b-octadecahydropicene-4a (2H)-carboxylate (**A4**)

White solid; Yield: 65%; mp: 205–206°C; ^1^H-NMR (300 MHz, DMSO-*d6*) δ: 9.20 (s, 1H, -CHO), 8.91 (s, 1H, triazole-H), 8.26 (s, 1H, Ar-H), 7.99 (d, *J* = 9 Hz, 1H, Ar-H), 7.89 (d, *J* = 9 Hz, 1H, Ar-H), 5.16 (t, *J* = 9 Hz, 3H), 4.85 (d, *J* = 3 Hz, 1H), 4.63 (d, *J* = 6 Hz, 1H), 3.62 (s, 1H), 2.91 (d, *J* = 9 Hz, 1H), 2.21 (t, *J* = 15 Hz, 1H), 1.90–1.74 (m, 3H), 1.67–1.46 (m, 7H), 1.35–1.27 (m, 6H), 1.14 (t, *J* = 15 Hz, 4H), 0.91 (m, 7H), 0.82 (d, *J* = 6 Hz, 2H), 0.65 (s, 3H), 0.63 (s, 3H). ^13^C-NMR (75 MHz, DMSO-*d6*)) δ: 207.48, 176.04, 143.76, 136.49, 132.88, 132.28, 131.48, 124.24, 122.04, 121.91, 120.33, 73.07, 70.82, 57.11, 55.50, 48.15, 46.66, 46.27, 41.31, 38.03, 35.66, 35.42, 34.90, 33.25, 32.14, 31.68, 30.62, 26.77, 26.37, 24.57, 23.17, 20.50, 16.70, 15.42, 9.20. ESI-HRMS calcd for C_39_H_52_N_3_O_5_Cl_2_^+^ ([M + H]^+^): 712.32785; found: 712.32629.

#### 4.2.5 (1-(4-bromophenyl)-1H-1,2,3-triazol-4-yl)methyl (4aR,5R,6aS,6bR,8aR,9S,10S,12aR,12bR,14bS)-9-formyl-5,10-dihydroxy-2,2,6a,6b,9,12a-hexamethyl-1,3,4,5,6,6a,6b,7,8,8a,9,10,11,12,12a,12b,13,14b-octadecahydropicene-4a (2H)-carboxylate (**A5**)

White solid; Yield: 56%; mp: 190–192°C; ^1^H-NMR (300 MHz, DMSO-*d6*) δ: 9.20 (s, 1H, -CHO), 8.82 (d, *J* = 3 Hz, 1H, triazole-H), 7.89 (d, *J* = 6 Hz, 2H, Ar-H), 7.80 (dd, *J*_*1*_ = 9 Hz, *J*_*2*_ = 3 Hz, 2H, Ar-H), 5.24–5.09 (m, 3H), 4.84 (s, 1H), 4.64 (s, 1H), 4.34 (s, 1H), 3.66 (t, *J* = 15 Hz, 1H), 2.91 (t, *J* = 15 Hz, 1H), 2.21 (t, *J* = 12 Hz, 1H), 2.06–1.47 (m, 10H), 1.40–1.23 (m, 7H), 1.19–1.06 (m, 4H), 0.98–0.83 (m, 12H), 0.76 (s, 1H), 0.62 (d, *J* = 6 Hz, 3H). ^13^C-NMR (75 MHz, DMSO-*d6*) δ: 207.50, 176.07, 143.74, 143.62, 136.18, 133.21, 123.83, 122.23, 121.91, 121.77, 86.44, 73.08, 70.83, 57.19, 55.51, 48.17, 46.69, 46.30, 41.35, 37.49, 35.67, 35.43, 34.96, 33.25, 32.16, 31.66, 30.63, 27.72, 26.79, 26.40, 24.57, 23.18, 20.53, 16.70, 15.48, 15.09, 13.72, 9.25. ESI-HRMS calcd for C_39_H_53_N_3_O_5_Br^+^ ([M + H]^+^): 722.31631; found: 722.31482.

#### 4.2.6 (1-(4-methoxyphenyl)-1H-1,2,3-triazol-4-yl)methyl (4aR,5R,6aS,6bR,8aR,9S,10S,12aR,12bR,14bS)-9-formyl-5,10-dihydroxy-2,2,6a,6b,9,12a-hexamethyl-1,3,4,5,6,6a,6b,7,8,8a,9,10,11,12,12a,12b,13,14b-octadecahydropicene-4a (2H)-carboxylate (**A6**)

White solid; Yield: 59%; mp: 192–193°C; ^1^H-NMR (300 MHz, DMSO-*d6*) δ: 9.21 (s, 1H, -CHO), 8.65 (s, 1H, triazole-H), 7.79 (d, *J* = 9 Hz, 2H, Ar-H), 7.13 (d, *J* = 9 Hz, 2H, Ar-H), 5.18–5.07 (m, 3H), 4.84 (d, *J* = 6 Hz, 1H), 4.65 (d, *J* = 3 Hz, 1H), 4.34 (s, 1H), 3.82 (s, 3H, -OCH_3_), 3.63 (s, 1H), 2.91 (d, *J* = 18 Hz, 1H), 2.22 (t, *J* = 12 Hz, 1H), 1.95–1.47 (m, 10H), 1.41–1.05 (m, 10H), 0.99–0.82 (m, 13H), 0.69 (s, 4H). ^13^C-NMR (75 MHz, DMSO-*d6*)) δ: 207.52, 176.09, 159.73, 143.79, 143.19, 130.40, 123.60, 122.00, 121.90, 115.28, 73.10, 70.83, 57.32, 56.03, 55.52, 48.18, 46.70, 46.33, 41.37, 38.08, 35.69, 34.94, 33.25, 32.17, 31.65, 30.63, 26.82, 26.39, 24.57, 23.20, 20.56, 16.75, 15.57, 9.25. ESI-HRMS calcd for C_40_H_56_N_3_O_6_^+^ ([M + H]^+^): 674.41636; found: 674.41486.

#### 4.2.7 (1-(3,4-dimethoxyphenyl)-1H-1,2,3-triazol-4-yl)methyl (4aR,5R,6aS,6bR,8aR,9S,10S,12aR,12bR,14bS)-9-formyl-5,10-dihydroxy-2,2,6a,6b,9,12a-hexamethyl-1,3,4,5,6,6a,6b,7,8,8a,9,10,11,12,12a,12b,13,14b-octadecahydropicene-4a (2H)-carboxylate (**A7**)

White solid; Yield: 71%; mp: 176–178°C; ^1^H-NMR (300 MHz, CDCl_3_) δ: 9.38 (s, 1H, -CHO), 7.96 (s, 1H, triazole-H), 7.33 (s, 1H, Ar-H), 7.15 (d, *J* = 6 Hz, 1H, Ar-H), 6.96 (d, *J* = 9 Hz, 1H, Ar-H), 5.39 (s, 1H, C_12_-H), 5.28–5.18 (m, 2H, -COO-CH_2_-), 4.57 (m, 1H, C_16_-H), 3.97 (s, 3H, -OCH_3_), 3.95 (s, 3H, -OCH_3_), 3.77 (t, *J* = 6 Hz, 1H, C_3_-H), 3.06 (d, *J* = 9 Hz, 1H, C_18_-H), 2.18 (t, *J* = 15 Hz, 2H), 1.93–1.85 (m, 3H), 1.75 (d, *J* = 12 Hz, 4H), 1.70–1.63 (m, 4H), 1.46 (d, *J* = 12 Hz, 1H), 1.36 (s, 3H), 1.27 (s, 2H), 1.03 (s, 3H), 0.96 (s, 3H), 0.89 (d, *J* = 6 Hz, 5H), 0.85 (d, *J* = 6 Hz, 5H), 0.47 (s, 3H). ^13^C-NMR (75 MHz, CDCl_3_) δ: 207.04, 176.73, 149.82, 149.52, 143.23, 142.83, 130.41, 122.59, 122.46, 112.33, 111.17, 104.76, 74.64, 71.80, 57.60, 56.26, 55.19, 48.64, 48.13, 46.52, 46.37, 41.39, 40.66, 39.76, 38.05, 35.92, 35.44, 32.76, 32.24, 30.94, 30.42, 26.85, 26.09, 24.50, 23.21, 20.67, 16.75, 15.59, 8.90. ESI-HRMS calcd for C_41_H_58_N_3_O_7_^+^ ([M + H]^+^): 704.42693; found: 704.42505.

#### 4.2.8 (1-(3,4,5-trimethoxyphenyl)-1H-1,2,3-triazol-4-yl)methyl (4aR,5R,6aS,6bR,8aR,9S,10S,12aR,12bR,14bS)-9-formyl-5,10-dihydroxy 2,2,6a,6b,9,12a-hexamethyl-1,3,4,5,6,6a,6b,7,8,8a,9,10,11,12,12a,12b,13,14b octadecahydropicene-4a (2H)-carboxylate (**A8**)

White solid; Yield: 70%; mp: 170–171°C; ^1^H-NMR (300 MHz, CDCl_3_) δ: 9.38 (s, 1H, -CHO), 7.99 (s, 1H, triazole-H), 6.93 (s, 2H, Ar-H), 5.39 (s, 1H, C_12_-H), 5.23 (dd, *J*_*1*_ = 18 Hz, *J*_*2*_ = 12 Hz, 2H, -COO-CH_2_-), 4.57 (s, 1H, C_16_-H), 3.94 (s, 6H, -OCH_3_), 3.89 (s, 3H, -OCH_3_), 3.77 (d, *J* = 6 Hz, 1H, C_3_-H), 3.05 (d, *J* = 6 Hz, 1H, C_18_-H), 2.18 (t, *J* = 15 Hz, 2H), 2.05 (s, 1H), 1.90 (d, *J* = 18 Hz, 2H), 1.77–1.62 (m, 9H), 1.46 (d, *J* = 12 Hz, 1H), 1.36 (s, 3H), 1.27–1.09 (m, 7H), 1.03 (s, 3H), 0.96 (s, 3H), 0.90 (s, 3H), 0.84 (s, 3H), 0.46 (s, 3H). ^13^C-NMR (75 MHz, CDCl_3_) δ: 207.01, 176.77, 153.96, 143.31, 142.86, 138.43, 132.66, 122.67, 122.44, 98.23, 74.64, 71.80, 61.08, 57.54, 56.48, 55.17, 48.63, 48.11, 46.50, 46.36, 41.40, 40.66, 39.75, 38.04, 35.92, 35.45, 35.41, 32.75, 32.24, 30.98, 30.42, 26.85, 26.08, 24.46, 23.21, 20.67, 16.72, 15.59, 8.90. ESI-HRMS calcd for C_40_H_56_N_3_O_6_^+^ ([M + H]^+^): 674.41636; found: 674.41357.

#### 4.2.9 (1-(p-tolyl)-1H-1,2,3-triazol-4-yl)methyl (4aR,5R,6aS,6bR,8aR,9S,10S,12aR,12bR,14bS)-9-formyl-5,10-dihydroxy-2,2,6a,6b,9,12a-hexamethyl-1,3,4,5,6,6a,6b,7,8,8a,9,10,11,12,12a,12b,13,14b-octadecahydropicene-4a (2H)-carboxylate (**A9**)

White solid; Yield: 56%; mp: 204–206°C; ^1^H-NMR (300 MHz, DMSO-*d6*) δ: 9.20 (s, 1H, -CHO), 8.70 (s, 1H, triazole-H), 7.76 (d, *J* = 6 Hz, 2H, Ar-H), 7.39 (d, *J* = 6 Hz, 2H, Ar-H), 5.18–5.07 (m, 3H), 4.85 (s, 1H), 4.65 (s, 1H), 4.34 (s, 1H), 3.63 (s, 1H), 2.91 (d, *J* = 12 Hz, 1H), 2.37 (s, 3H), 2.22 (t, *J* = 12 Hz, 1H), 1.90–1.67 (m, 5H), 1.62–1.47 (m, 5H), 1.41–1.28 (m, 6H), 1.20–1.05 (m, 5H), 0.98–0.90 (m, 6H), 0.82 (s, 6H), 0.67 (s, 4H). ^13^C-NMR (75 MHz, DMSO-*d6*) δ: 207.50, 176.08, 143.79, 143.30, 138.75, 134.75, 130.61, 123.56, 121.90, 120.26, 73.10, 70.83, 57.29, 55.52, 48.18, 46.70, 46.33, 41.36, 35.69, 35.42, 34.92, 33.24, 32.17, 31.66, 30.62, 26.82, 26.40, 24.58, 23.20, 21.01, 20.52, 16.74, 15.53, 9.24. ESI-HRMS calcd for C_40_H_56_N_3_O_5_^+^ ([M + H]^+^): 658.42145; found: 658.41998.

#### 4.2.10 (1-(4-nitrophenyl)-1H-1,2,3-triazol-4-yl)methyl (4aR,5R,6aS,6bR,8aR,9S,10S,12aR,12bR,14bS)-9-formyl-5,10-dihydroxy-2,2,6a,6b,9,12a-hexamethyl-1,3,4,5,6,6a,6b,7,8,8a,9,10,11,12,12a,12b,13,14b-octadecahydropicene-4a (2H)-carboxylate (**A10**)

Yellow solid; Yield: 59%; mp: 193–194°C; ^1^H-NMR (300 MHz, DMSO-*d6*) δ: 9.19 (s, 1H, -CHO), 9.03 (s, 1H, triazole-H), 8.47 (d, *J* = 9 Hz, 2H, Ar-H), 8.25 (d, *J* = 9 Hz, 2H, Ar-H), 5.17 (s, 3H), 4.85 (s, 1H), 4.63 (d, *J* = 6 Hz, 1H), 4.34 (s, 1H), 3.62 (s, 1H), 2.91 (d, *J* = 12 Hz, 1H), 2.37 (s, 3H), 2.22 (t, *J* = 15 Hz, 1H), 1.91–1.65 (m, 5H), 1.56–1.40 (m, 6H), 1.36–1.23 (m, 5H), 1.18–1.04 (m, 5H), 0.97–0.91 (m, 6H), 0.81 (d, *J* = 15 Hz, 6H), 0.61 (s, 4H). ^13^C-NMR (75 MHz, DMSO-*d6*)) δ: 207.49, 176.08, 147.17, 144.08, 143.71, 141.24, 126.03, 124.35, 121.94, 120.98, 73.08, 70.81, 57.08, 55.49, 48.18, 46.64, 46.27, 41.34, 38.03, 35.66, 35.40, 34.95, 33.25, 32.13, 31.65, 30.63, 26.80, 26.35, 24.57, 23.18, 20.55, 16.69, 15.46, 9.17. ESI-HRMS calcd for C_39_H_53_N_4_O_7_^+^ ([M + H]^+^): 689.39088; found: 689.38971.

#### 4.2.11 (5-phenyl-1,3,4-oxadiazol-2-yl)methyl (4aR,5R,6aS,6bR,8aR,9S,10S,12aR,12bR,14bS)-9-formyl-5,10-dihydroxy-2,2,6a,6b,9,12a-hexamethyl-1,3,4,5,6,6a,6b,7,8,8a,9,10,11,12,12a,12b,13,14b-octadecahydropicene-4a (2H)-carboxylate (**B1**)

White solid; Yield: 71%; mp: 130–132; ^1^H-NMR (300 MHz, CDCl_3_) δ: 9.41 (s, 1H, -CHO), 8.06 (d, *J* = 6 Hz, 2H, Ar-H), 7.59–7.51 (m, 3H), 5.41 (s, 1H, C_12_-H), 5.34–5.26 (m, 2H, -COO-CH_2_-), 4.59 (s, 1H, C_16_-H), 3.78 (d, *J* = 6 Hz, 1H, C_3_-H), 3.09 (d, *J* = 6 Hz, 1H, C_18_-H), 2.20 (t, *J* = 15 Hz, 1H), 1.95–1.80 (m, 6H), 1.68 (d, *J* = 18 Hz, 7H), 1.51 (s, 2H), 1.38 (s, 3H), 1.26 (t, *J* = 12 Hz, 3H), 1.03 (d, *J* = 15 Hz, 6H), 0.90 (d, *J* = 18 Hz, 7H), 0.65 (s, 3H). ^13^C-NMR (75 MHz, CDCl_3_) δ: 207.07, 175.72, 165.63, 161.66, 142.47, 132.17, 129.14, 127.02, 123.39, 122.79, 76.61, 74.64, 71.84, 55.34, 55.19, 49.02, 48.19, 46.58, 46.29, 41.41, 40.70, 39.80, 38.07, 35.93, 35.54, 35.37, 32.73, 32.24, 30.72, 30.39, 26.94, 26.11, 24.57, 23.26, 20.68, 16.94, 15.60, 8.92. ESI-HRMS calcd for C_39_H_53_N_2_O_6_^+^ ([M + H]^+^): 645.38981; found: 645.38849.

#### 4.2.12 (5-(4-fluorophenyl)-1,3,4-oxadiazol-2-yl)methyl (4aR,5R,6aS,6bR,8aR,9S,10S,12aR,12bR,14bS)-9-formyl-5,10-dihydroxy-2,2,6a,6b,9,12a-hexamethyl-1,3,4,5,6,6a,6b,7,8,8a,9,10,11,12,12a,12b,13,14b-octadecahydropicene-4a (2H)-carboxylate (**B2**)

White solid; Yield: 72%; mp: 122–124°C; Purity: 98.40%; ^1^H-NMR (300 MHz, CDCl_3_) δ: 9.42 (s, 1H, -CHO), 8.07 (t, *J* = 6 Hz, 2H, Ar-H), 7.23 (t, *J* = 9 Hz, 2H, Ar-H), 5.41 (s, 1H, C_12_-H), 5.31 (s, 2H, -COO-CH_2_-), 4.71–4.59 (m, 1H, C_16_-H), 3.80 (s, 1H, C_3_-H), 3.09 (d, *J* = 15 Hz, 1H, C_18_-H), 2.20 (t, *J* = 12 Hz, 1H), 1.95–1.80 (m, 5H), 1.78–1.61 (m, 8H), 1.52–1.39 (m, 6H), 1.34–1.23 (m, 4H), 1.07 (s, 4H), 0.99 (s, 3H), 0.93 (s, 3H), 0.89 (s, 3H), 0.66 (s, 3H). ^13^C-NMR (75 MHz, CDCl_3_) δ: 207.07, 175.74, 165.43 (d, *J* = 92.2 Hz), 164.02, 161.71, 142.48, 129.40, 129.33, 122.80, 119.74, 116.64, 116.46, 74.63, 71.88, 55.31, 55.18, 49.06, 48.23, 46.58, 46.28, 41.44, 40.74, 39.82, 38.09, 35.96, 35.56, 35.36, 32.72, 32.27, 30.68, 30.39, 26.96, 26.12, 24.59, 23.26, 20.68, 16.96, 15.61, 8.94. ESI-HRMS calcd for C_39_H_52_N_2_O_6_F^+^ ([M + H]^+^): 663.38039; found: 663.37909.

#### 4.2.13 (5-(4-chlorophenyl)-1,3,4-oxadiazol-2-yl)methyl (4aR,5R,6aS,6bR,8aR,9S,10S,12aR,12bR,14bS)-9-formyl-5,10-dihydroxy-2,2,6a,6b,9,12a-hexamethyl-1,3,4,5,6,6a,6b,7,8,8a,9,10,11,12,12a,12b,13,14b-octadecahydropicene-4a (2H)-carboxylate (**B3**)

White solid; Yield: 65%; mp: 132–134°C; Purity: 98.98%; ^1^H-NMR (300 MHz, CDCl_3_) δ: 9.41 (s, 1H, -CHO), 8.00 (d, *J* = 9 Hz, 2H, Ar-H), 7.52 (d, *J* = 9 Hz, 2H, Ar-H), 5.40 (s, 1H, C_12_-H), 5.31 (t, *J* = 15 Hz, 2H, -COO-CH_2_-), 4.59 (s, 1H, C_16_-H), 3.78 (t, *J* = 6 Hz, 1H, C_3_-H), 3.07 (dd, *J*_*1*_ = 12 Hz, *J*_*2*_ = 3 Hz, 1H, C_18_-H), 2.19 (t, *J* = 15 Hz, 1H), 1.95–1.84 (m, 4H), 1.78 (s, 1H), 1.70 (d, *J* = 9 Hz, 6H), 1.57–1.47 (m, 3H), 1.38 (s, 3H), 1.26 (t, *J* = 15 Hz, 3H), 1.17–1.11 (m, 2H), 1.06 (s, 3H), 0.98 (s, 3H), 0.93 (s, 3H), 0.88 (s, 3H), 0.64 (s, 3H). ^13^C-NMR (75 MHz, CDCl_3_) δ: 207.09, 175.74, 164.81, 161.84, 142.45, 138.54, 129.56, 128.29, 122.79, 121.85, 74.60, 71.85, 55.30, 55.18, 49.04, 48.20, 46.56, 46.26, 41.42, 40.71, 39.80, 38.07, 35.93, 35.54, 35.34, 32.72, 32.25, 30.68, 30.39, 26.94, 26.10, 24.58, 23.25, 20.67, 16.96, 15.60, 8.94. ESI-HRMS calcd for C_39_H_52_N_2_O_6_Cl^+^ ([M + H]^+^): 679.35084; found: 679.34991.

#### 4.2.14 (5-(4-methoxyphenyl)-1,3,4-oxadiazol-2-yl)methyl (4aR,5R,6aS,6bR,8aR,9S,10S,12aR,12bR,14bS)-9-formyl-5,10-dihydroxy-2,2,6a,6b,9,12a-hexamethyl-1,3,4,5,6,6a,6b,7,8,8a,9,10,11,12,12a,12b,13,14b-octadecahydropicene-4a (2H)-carboxylate (**B4**)

White solid; Yield: 65%; mp: 126–128°C; Purity: 97.88%; ^1^H-NMR (300 MHz, CDCl_3_) δ: 9.42 (s, 1H, -CHO), 7.99 (d, *J* = 9 Hz, 2H, Ar-H), 7.03 (d, *J* = 9 Hz, 2H, Ar-H), 5.41 (t, *J* = 6 Hz, 1H, C_12_-H), 5.31 (t, *J* = 6 Hz, 2H, -COO-CH_2_-), 4.60 (s, 1H, C_16_-H), 3.91 (s, 3H, -OCH_3_), 3.80 (s, 1H, C_3_-H), 3.08 (d, *J* = 12 Hz, 1H, C_18_-H), 2.19 (t, *J* = 12 Hz, 1H), 1.96–1.84 (m, 5H), 1.80 (t, *J* = 6 Hz, 1H), 1.71–1.62 (m, 7H), 1.52–1.46 (m, 3H), 1.38 (s, 4H), 1.34–1.22 (m, 4H), 1.06 (s, 4H), 0.99 (s, 3H), 0.93 (s, 3H), 0.89 (s, 3H), 0.66 (s, 3H). ^13^C-NMR (75 MHz, CDCl_3_) δ: 207.09, 175.75, 165.60, 162.67, 161.16, 142.51, 128.84, 122.78, 115.86, 114.57, 74.61, 71.85, 55.53, 55.37, 55.21, 49.04, 48.19, 46.60, 46.30, 41.42, 40.71, 39.81, 38.09, 35.95, 35.54, 35.37, 32.73, 32.25, 30.68, 30.39, 26.95, 26.11, 24.60, 23.26, 20.69, 16.96, 15.62, 8.92. ESI-HRMS calcd for C_40_H_55_N_2_O_7_^+^ ([M + H]^+^): 675.40038; found: 675.39911.

#### 4.2.15 (5-(3,4,5-trimethoxyphenyl)-1,3,4-oxadiazol-2-yl)methyl (4aR,5R,6aS,6bR,8aR,9S,10S,12aR,12bR,14bS)-9-formyl-5,10-dihydroxy-2,2,6a,6b,9,12a-hexamethyl-1,3,4,5,6,6a,6b,7,8,8a,9,10,11,12,12a,12b,13,14b-octadecahydropicene-4a (2H)-carboxylate (**B5**)

White solid; Yield: 66%; mp: 136–138°C; Purity: 97.48%; ^1^H-NMR (300 MHz, CDCl_3_) δ: 9.40 (s, 1H, -CHO), 5.40 (s, 1H, C_12_-H), 5.31 (s, 2H, -COO-CH_2_-), 4.59 (s, 1H, C_16_-H), 3.95 (s, 6H, -OCH_3_), 3.93 (s, 3H, -OCH_3_), 3.78 (d, *J* = 9 Hz, 1H, C_3_-H), 3.08 (dd, *J*_*1*_ = 15 Hz, *J*_*2*_ = 3 Hz, 1H, C_18_-H), 2.20 (t, *J* = 15 Hz, 1H), 1.95–1.78 (m, 6H), 1.68 (s, 7H), 1.51 (t, *J* = 12 Hz, 2H), 1.38 (s, 3H), 1.25 (t, t, *J* = 9 Hz, 3H), 1.17–1.11 (m, 2H), 1.05 (s, 3H), 0.99 (s, 3H), 0.90 (d, *J* = 18 Hz, 7H), 0.63 (s, 3H). ^13^C-NMR (75 MHz, CDCl_3_) δ: 207.06, 175.76, 165.50, 161.54, 153.73, 142.47, 141.50, 122.78, 118.39, 104.34, 74.58, 71.84, 61.05, 56.42, 55.30, 55.17, 49.05, 48.17, 46.56, 46.22, 41.43, 40.78, 39.79, 38.07, 35.91, 35.55, 35.34, 32.72, 32.24, 30.61, 30.38, 26.95, 26.09, 24.63, 23.27, 20.68, 16.94, 15.58, 8.93. ESI-HRMS calcd for C_42_H_59_N_2_O_9_^+^ ([M + H]^+^): 735.42151; found: 735.42041.

#### 4.2.16 (5-(4-nitrophenyl)-1,3,4-oxadiazol-2-yl)methyl (4aR,5R,6aS,6bR,8aR,9S,10S,12aR,12bR,14bS)-9-formyl-5,10-dihydroxy-2,2,6a,6b,9,12a-hexamethyl-1,3,4,5,6,6a,6b,7,8,8a,9,10,11,12,12a,12b,13,14b-octadecahydropicene-4a (2H)-carboxylate (**B6**)

White solid; Yield: 66%; mp: 140–142°C; Purity: 98.55%; ^1^H-NMR (300 MHz, CDCl_3_) δ: 9.41 (s, 1H, -CHO), 8.41 (d, *J* = 9 Hz, 2H, Ar-H), 8.26 (d, *J* = 9 Hz, 2H, Ar-H), 5.41 (s, 1H, C_12_-H), 5.34 (s, 2H, -COO-CH_2_-), 4.59 (s, 1H, C_16_-H), 3.79 (d, *J* = 6 Hz, 1H, C_3_-H), 3.08 (dd, *J*_*1*_ = 15 Hz, *J*_*2*_ = 6 Hz, 1H, C_18_-H), 2.20 (t, *J* = 12 Hz, 1H), 1.96 (s, 1H), 1.89–1.84 (m, 3H), 1.72–1.66 (m, 6H), 1.51 (t, *J* = 6 Hz, 2H), 1.39 (s, 3H), 1.27 (d, *J* = 12 Hz, 4H), 1.18–1.11 (m, 2H), 1.06 (s, 3H), 0.99 (s, 4H), 0.93–0.87 (m, 7H), 0.66 (s, 3H). ^13^C-NMR (75 MHz, CDCl_3_) δ: 207.07, 175.75, 163.80, 162.75, 149.82, 142.44, 128.84, 127.99, 124.46, 122.81, 74.58, 71.87, 55.27, 55.17, 49.07, 48.18, 46.54, 46.24, 41.45, 40.75, 39.82, 38.07, 35.94, 35.56, 35.32, 32.71, 32.26, 30.68, 30.38, 26.95, 26.10, 24.58, 23.27, 20.67, 16.97, 15.63, 8.96. ESI-HRMS calcd for C_39_H_52_N_3_O_8_^+^ ([M + H]^+^): 690.37489; found: 690.37347.

#### 4.2.17 2-(5-oxo-4-phenyl-4,5-dihydro-1H-1,2,4-triazol-1-yl)ethyl (4aR,5R,6aS,6bR,8aR,9S,10S,12aR,12bR,14bS)-9-formyl-5,10-dihydroxy-2,2,6a,6b,9,12a-hexamethyl-1,3,4,5,6,6a,6b,7,8,8a,9,10,11,12,12a,12b,13,14b-octadecahydropicene-4a (2H)-carboxylate (**C1**)

White solid; Yield: 70%; mp: 174–176°C; Purity: 99.00%; ^1^H-NMR (300 MHz, CDCl_3_) δ: 9.41 (s, 1H, -CHO), 7.72 (s, 1H, triazole-H), 7.58–7.48 (m, 4H, Ar-H), 7.39 (d, *J* = 9 Hz, 1H, Ar-H), 5.42 (s, 1H, C_12_-H), 4.49–4.42 (m, 2H, -COO-CH_2_-), 4.33–4.25 (m, 1H, C_16_-H), 4.13 (t, *J* = 6 Hz, 2H, -N-CH_2_-), 3.78 (t, *J* = 6 Hz, 1H, C_3_-H), 3.06 (dd, *J*_*1*_ = 15 Hz, *J*_*2*_ = 6 Hz, 1H, C_18_-H), 2.13–2.04 (m, 2H), 1.92–1.88 (m, 2H), 1.78–1.60 (m, 9H), 1.52–1.44 (m, 3H), 1.33–1.24 (m, 6H), 1.18–1.07 (s, 6H), 0.93 (d, *J* = 12 Hz, 10H), 0.74 (s, 3H). ^13^C-NMR (75 MHz, CDCl_3_) δ: 207.05, 176.62, 151.87, 142.47, 133.94, 133.75, 129.71, 127.73, 122.46, 121.74, 73.93, 71.82, 61.82, 55.23, 49.41, 48.22, 46.79, 46.00, 44.47, 41.49, 40.97, 40.00, 38.13, 35.95, 35.45, 34.98, 32.66, 32.21, 30.20, 29.13, 27.06, 26.12, 25.34, 23.27, 20.70, 17.15, 15.81, 8.91. ESI-HRMS calcd for C_40_H_56_N_3_O_6_^+^ ([M + H]^+^): 674.41636; found: 674.41486.

#### 4.2.18 2-(4-(4-fluorophenyl)-5-oxo-4,5-dihydro-1H-1,2,4-triazol-1-yl)ethyl (4aR,5R,6aS,6bR,8aR,9S,10S,12aR,12bR,14bS)-9-formyl-5,10-dihydroxy-2,2,6a,6b,9,12a-hexamethyl-1,3,4,5,6,6a,6b,7,8,8a,9,10,11,12,12a,12b,13,14b-octadecahydropicene-4a (2H)-carboxylate (**C2**)

White solid; Yield: 74%; mp: 176–178°C; ^1^H-NMR (300 MHz, CDCl_3_) δ: 9.39 (s, 1H, -CHO), 7.69 (d, *J* = 3 Hz, 1H, triazole-H), 7.52 (dd, *J*_*1*_ = 9 Hz, *J*_*2*_ = 3 Hz, 2H, Ar-H), 7.19 (t, *J* = 6 Hz, 2H, Ar-H), 5.41 (s, 1H, C_12_-H), 4.46–4.40 (m, 2H, -COO-CH_2_-), 4.31–4.24 (m, 1H, C_16_-H), 4.11 (t, *J* = 6 Hz, 2H, -N-CH_2_-), 3.78 (d, *J* = 6 Hz, 1H, C_3_-H), 3.04 (d, *J* = 12 Hz, 1H, C_18_-H), 2.20–2.03 (m, 2H), 1.90 (d, *J* = 6 Hz, 2H), 1.81–1.63 (m, 10H), 1.49 d, *J* = 9 Hz, 2H), 1.37–1.27 (m, 6H), 1.13 (t, *J* = 6 Hz, 2H), 1.06 (s, 3H), 0.95 (d, *J* = 3 Hz, 6H), 0.90 (s, 3H), 0.84 (s, 1H), 0.73 (s, 3H). ^13^C-NMR (75 MHz, CDCl_3_) δ: 207.11, 176.62, 161.65 (d, *J* = 246 Hz), 151.90, 142.46, 133.94, 129.72, 123.96 (d, *J* = 8.25 Hz), 122.45, 116.68 (d, *J* = 23.25 Hz), 73.90, 71.81, 61.77, 55.26, 49.40, 48.19, 46.77, 45.97, 44.52, 41.49, 40.96, 40.00, 38.13, 35.94, 35.42, 34.96, 32.65, 32.21, 30.20, 29.10, 27.05, 26.10, 25.33, 23.27, 20.70, 17.15, 15.81, 8.92. ESI-HRMS calcd for C_40_H_55_N_3_O_6_F^+^ ([M + H]^+^): 692.40694; found: 692.40527.

#### 4.2.19 2-(4-(4-chlorophenyl)-5-oxo-4,5-dihydro-1H-1,2,4-triazol-1-yl)ethyl (4aR,5R,6aS,6bR,8aR,9S,10S,12aR,12bR,14bS)-9-formyl-5,10-dihydroxy-2,2,6a,6b,9,12a-hexamethyl-1,3,4,5,6,6a,6b,7,8,8a,9,10,11,12,12a,12b,13,14b-octadecahydropicene-4a (2H)-carboxylate (**C3**)

White solid; Yield: 65%; mp: 166–168°C; Purity: 97.39%; ^1^H-NMR (300 MHz, CDCl_3_) δ: 9.39 (s, 1H, -CHO), 7.71 (s, 1H, triazole-H), 7.53 (d, *J* = 9 Hz, 2H, Ar-H), 7.47 (d, *J* = 9 Hz, 2H, Ar-H), 5.40 (s, 1H, C_12_-H), 4.44–4.39 (m, 2H, -COO-CH_2_-), 4.31–4.24 (m, 1H, C_16_-H), 4.11 (t, *J* = 6 Hz, 2H, -N-CH_2_-), 3.78 (d, *J* = 9 Hz, 1H, C_3_-H), 3.03 (dd, *J*_*1*_ = 15 Hz, *J*_*2*_ = 3 Hz, 1H, C_18_-H), 2.18–2.03 (m, 2H), 1.91–1.87 (m, 2H), 1.76–1.67 (m, 9H), 1.48 (t, *J* = 9 Hz, 2H), 1.40–1.22 (m, 7H), 1.13 (t, *J* = 6 Hz, 3H), 1.06 (s, 3H), 0.94 (s, 6H), 0.86 (s, 1H), 0.90 (s, 3H), 0.72 (s, 3H). ^13^C-NMR (75 MHz, CDCl_3_) δ: 207.11, 176.62, 151.68, 142.45, 133.53, 133.45, 132.26, 129.87, 122.87, 122.45, 73.91, 71.81, 61.74, 55.24, 49.40, 48.21, 46.76, 45.98, 44.54, 41.48, 40.95, 39.99, 38.13, 35.94, 35.41, 34.96, 32.65, 32.20, 30.20, 29.11, 27.05, 26.10, 25.33, 23.27, 20.70, 17.13, 15.81, 8.93. ESI-HRMS calcd for C_40_H_55_N_3_O_6_Cl^+^ ([M + H]^+^): 708.37739; found: 708.37628.

#### 4.2.20 2-(4-(3,4-dichlorophenyl)-5-oxo-4,5-dihydro-1H-1,2,4-triazol-1-yl)ethyl (4aR,5R,6aS,6bR,8aR,9S,10S,12aR,12bR,14bS)-9-formyl-5,10-dihydroxy-2,2,6a,6b,9,12a-hexamethyl-1,3,4,5,6,6a,6b,7,8,8a,9,10,11,12,12a,12b,13,14b-octadecahydropicene-4a (2H)-carboxylate (**C4**)

White solid; Yield: 55%; mp: 187–189°C; ^1^H-NMR (300 MHz, CDCl_3_) δ: 9.41 (s, 1H, -CHO), 7.77 (s, 1H, triazole-H), 7.73 (s, 1H, Ar-H), 7.58 (d, *J* = 9 Hz, 1H, Ar-H), 7.48 (d, *J* = 9 Hz, 1H, Ar-H), 5.39 (s, 1H, C_12_-H), 4.41 (s, 2H, -COO-CH_2_-), 4.30 (s, 1H, C_16_-H), 4.11 (s, 2H, -N-CH_2_-), 3.78 (s, 1H, C_3_-H), 3.04 (d, *J* = 12 Hz, 1H, C_18_-H), 2.08 (t, *J* = 12 Hz, 2H), 1.85 (d, *J* = 12 Hz, 3H), 1.69 (d, *J* = 12 Hz, 8H), 1.49 (t, *J* = 12 Hz, 3H), 1.30 (t, *J* = 18 Hz, 6H), 1.07 (s, 6H), 0.93 (d, *J* = 12 Hz, 9H), 0.72 (s, 3H). ^13^C-NMR (75 MHz, CDCl_3_) δ: 207.05, 176.58, 151.38, 142.45, 133.83, 133.01, 131.78, 131.36, 123.27, 122.45, 120.48, 74.01, 71.84, 61.66, 55.20, 49.36, 48.25, 46.74, 45.99, 44.64, 41.48, 40.93, 39.99, 38.12, 35.96, 35.44, 34.99, 32.65, 32.22, 30.22, 29.22, 27.06, 26.11, 25.28, 23.28, 20.70, 17.13, 15.81, 8.93. ESI-HRMS calcd for C_40_H_54_N_3_O_6_Cl_2_^+^ ([M + H]^+^): 742.33842; found: 742.33636.

#### 4.2.21 2-(4-(4-methoxyphenyl)-5-oxo-4,5-dihydro-1H-1,2,4-triazol-1-yl)ethyl (4aR,5R,6aS,6bR,8aR,9S,10S,12aR,12bR,14bS)-9-formyl-5,10-dihydroxy-2,2,6a,6b,9,12a-hexamethyl-1,3,4,5,6,6a,6b,7,8,8a,9,10,11,12,12a,12b,13,14b-octadecahydropicene-4a (2H)-carboxylate (**C5**)

White solid; Yield: 58%; mp: 186–188°C; Purity: 98.33%; ^1^H-NMR (300 MHz, CDCl_3_) δ: 9.40 (s, 1H, -CHO), 7.64 (s, 1H, triazole-H), 7.43 (d, *J* = 9 Hz, 2H, Ar-H), 7.00 (d, *J* = 9 Hz, 2H, Ar-H), 5.42 (s, 1H, C_12_-H), 4.47–4.40 (m, 2H, -COO-CH_2_-), 4.31–4.24 (m, 1H, C_16_-H), 4.11 (t, *J* = 2 Hz, 2H, -N-CH_2_-), 3.86 (s, 3H, -OCH_3_), 3.79 (d, *J* = 9 Hz, 1H, C_3_-H), 3.05 (dd, *J*_*1*_ = 12 Hz, *J*_*2*_ = 3 Hz, 1H, C_18_-H), 2.20–2.03 (m, 2H), 1.92–1.82 (m, 3H), 1.75–1.63 (m, 10H), 1.49 (t, *J* = 9 Hz, 2H), 1.38–1.24 (m, 6H), 1.16–1.09 (m, 2H), 1.06 (s, 3H), 0.95 (s, 6H), 0.91 (S, 3H), 0.74 (s, 3H). ^13^C-NMR (75 MHz, CDCl_3_) δ: 207.05, 176.63, 159.05, 152.17, 142.44, 134.42, 126.55, 123.81, 122.46, 114.84, 73.86, 71.82, 61.87, 55.60, 55.24, 49.46, 48.23, 46.80, 45.98, 44.47, 41.50, 41.01, 40.02, 38.13, 35.96, 35.45, 34.95, 32.65, 32.21, 30.19, 28.99, 27.07, 26.12, 25.41, 23.28, 20.72, 17.16, 15.82, 8.91. ESI-HRMS calcd for C_41_H_58_N_3_O_7_^+^ ([M + H]^+^): 704.42693; found: 704.42523.

#### 4.2.22 2-(5-oxo-4-(3,4,5-trimethoxyphenyl)-4,5-dihydro-1H-1,2,4-triazol-1-yl)ethyl (4aR,5R,6aS,6bR,8aR,9S,10S,12aR,12bR,14bS)-9-formyl-5,10-dihydroxy-2,2,6a,6b,9,12a-hexamethyl-1,3,4,5,6,6a,6b,7,8,8a,9,10,11,12,12a,12b,13,14b-octadecahydropicene-4a (2H)-carboxylate (**C6**)

White solid; Yield: 67%; mp: 174–176°C; Purity: 98.72%; ^1^H-NMR (300 MHz, CDCl_3_) δ: 9.40 (s, 1H, -CHO), 7.68 (s, 1H, triazole-H), 6.75 (s, 2H, Ar-H), 5.42 (s, 1H, C_12_-H), 4.45–4.38 (m, 2H, -COO-CH_2_-), 4.33–4.26 (m, 1H, C_16_-H), 4.11 (t, *J* = 6 Hz, 2H, -N-CH_2_-), 3.90 (s, 6H, -OCH_3_), 3.87 (s, 3H, -OCH_3_), 3.78 (dd, *J*_*1*_ = 9 Hz, *J*_*2*_ = 6 Hz, 1H, C_3_-H), 3.06 (d, *J* = 6 Hz, 1H, C_18_-H), 2.07 (t, *J* = 12 Hz, 1H), 1.91–1.82 (m, 4H), 1.77–1.64 (m, 10H), 1.50 (t, *J* = 9 Hz, 3H), 1.38 (d, *J* = 6 Hz, 1H), 1.32 (s, 3H), 1.27 (s, 1H), 1.12 (t, *J* = 6 Hz, 2H), 1.06 (s, 3H), 0.95 (s, 6H), 0.88 (d, *J* = 6 Hz, 4H), 0.75 (s, 3H). ^13^C-NMR (75 MHz, CDCl_3_) δ: 207.04, 176.60, 153.88, 151.99, 142.38, 134.24, 129.33, 122.49, 100.11, 73.85, 71.84, 61.81, 60.99, 56.40, 55.22, 49.50, 48.23, 46.80, 44.44, 41.52, 41.03, 40.04, 38.13, 35.96, 35.45, 34.94, 32.64, 32.24, 30.18, 28.91, 27.09, 26.10, 25.46, 23.29, 20.72, 17.20, 15.82, 8.93. ESI-HRMS calcd for C_43_H_62_N_3_O_9_^+^ ([M + H]^+^): 764.44806; found: 764.44617.

#### 4.2.23 (3-phenylisoxazol-5-yl)methyl (4aR,5R,6aS,6bR,8aR,9S,10S,12aR,12bR,14bS)-9-formyl-5,10-dihydroxy-2,2,6a,6b,9,12a-hexamethyl-1,3,4,5,6,6a,6b,7,8,8a,9,10,11,12,12a,12b,13,14b-octadecahydropicene-4a (2H)-carboxylate (**D**)

White solid; Yield: 66%; mp: 176–178°C; ^1^H-NMR (300 MHz, CDCl_3_) δ: 9.41 (s, 1H, -CHO), 7.81 (t, *J* = 6 Hz, 2H, Ar-H), 7.49 (d, *J* = 3 Hz, 3H, Ar-H), 6.60 (s, 1H, isoxazole-H), 5.42 (s, 1H, C_12_-H), 5.19 (s, 2H, -COO-CH_2_-), 4.59 (s, 1H, C_16_-H), 3.80 (s, 1H, C_3_-H), 3.09 (d, *J* = 15 Hz, 1H, C_18_-H), 2.20 (t, *J* = 15 Hz, 1H), 1.93–1.67 (m, 9H), 1.59 (s, 3H), 1.53–1.39 (m, 7H), 1.33–1.18 (m, 4H), 1.15–0.88 (m, 14H), 0.62 (s, 3H). ^13^C-NMR (75 MHz, CDCl_3_) δ: 207.07, 176.02, 167.09, 162.49, 142.56, 130.21, 128.97, 128.65, 126.79, 122.79, 102.36, 74.77, 71.83, 56.36, 55.19, 48.78, 48.20, 46.58, 46.31, 41.41, 40.74, 39.82, 38.08, 35.94, 35.53, 35.41, 32.76, 32.30, 30.94, 30.43, 26.91, 26.11, 24.48, 23.27, 20.67, 17.00, 15.57, 8.90. ESI-HRMS calcd for C_40_H_54_NO_6_^+^ ([M + H]^+^): 644.39456; found: 644.39355.

### 4.3 General procedure for synthesis of compounds **E**

Stir a mixture of quillaic acid (50 mg, 0.10 mmol), 2 equivalents of K_2_CO_3_, catalytic amount KI, and 2 equivalents of 1,2-dibromoethane in DMF at 60°C. The reaction was monitored by TLC. After quenching the reaction with cold water, extracting with dichloromethane, collecting the organic phase and evaporating to dryness to obtain a crude product. The crude product was added K_2_CO_3_, KI and 5-phenyl-1*H*-tetrazole in the DMF solution, and reacted at 60°C for 8 h. After the reaction was quenched with cold water, extracted with dichloromethane, the organic phase was collected and evaporated to dryness, and purified by silica gel column chromatography to obtain the target compound **E**.

#### 4.3.1 2-(5-phenyl-1H-tetrazol-1-yl)ethyl (4aR,5R,6aS,6bR,8aR,9S,10S,12aR,12bR,14bS)-9-formyl-5,10-dihydroxy-2,2,6a,6b,9,12a-hexamethyl-1,3,4,5,6,6a,6b,7,8,8a,9,10,11,12,12a,12b,13,14b-octadecahydropicene-4a (2H)-carboxylate (**E**)

White solid; Yield: 32%; mp: 196–198°C; Purity: 98.23%; ^1^H-NMR (300 MHz, CDCl_3_) δ: 9.38 (s, 1H, -CHO), 8.17 (t, *J* = 3 Hz, 2H, Ar-H), 7.51 (t, *J* = 3 Hz, 3H, Ar-H), 5.27 (t, *J* = 3 Hz, 1H, C_12_-H), 4.96–4.89 (m, 2H, -COO-CH_2_-), 4.71–4.65 (s, 1H, C_16_-H), 4.52–4.41 (m, 2H, -N-CH_2_-), 3.76 (d, *J* = 9 Hz, 1H, C_3_-H), 2.95 (d, *J* = 12 Hz, 1H, C_18_-H), 2.12 (t, *J* = 12 Hz, 1H), 1.84–1.73 (m, 6H), 1.65–1.59 (m, 5H), 1.53–1.15 (m, 11H), 1.04 (s, 5H), 0.89 (t, *J* = 12 Hz, 9H), 0.85 (s, 3H), 0.54 (s, 3H). ^13^C-NMR (75 MHz, CDCl_3_) δ: 206.97, 176.20, 165.48, 142.46, 130.53, 128.95, 127.15, 126.86, 122.63, 74.52, 71.80, 61.67, 55.16, 51.80, 48.73, 48.16, 46.55, 46.17, 41.27, 40.45, 39.71, 38.02, 35.90, 35.38, 35.33, 32.71, 32.03, 30.77, 30.34, 26.89, 26.09, 24.49, 23.16, 20.64, 16.75, 15.60, 8.86. ESI-HRMS calcd for C_39_H_55_N_4_O_5_^+^ ([M + H]^+^): 659.41670; found: 659.41547.

### 4.4 Cell lines and cell culture

The propidium iodide (PI) and Annexin V-FITC apoptosis detection kit were purchased from Invitrogen (Eugene, OR, United States). 3-(4,5-Dimethylthiazol-2-yl)-2,5-diphenyl-2H-tetrazolium bromide (MTT) was purchased from Sigma-Aldrich Co. (St. Louis, MO, United States). All human cell lines were used in this study, colorectal cancer cell HCT116, colorectal cancer cell SW620, breast cancer cell MCF-7, liver hepatocellular carcinoma cell BEL7402, liver hepatocellular carcinoma HepG2 and normal liver cell L02 were initially purchased from American Type Culture Collection (ATCC, Manassas, VA, United States). RPMI-1640 media, Dulbecco’s modified Eagle’s medium (DMEM), and foetal bovine serum (FBS) were provided from Gibco Company (Grand Island, NY, United States). Cell line MCF-7 and HepG2 were cultivated in DMEM containing 10% (v/v) heat-inactivated FBS, 100 units/ml penicillin and 100 mg/ml streptomycin (Grand Island, NY, United States). Cell lines HCT116, BEL7402, SW620, and L02 were cultivated in RPMI 1640 medium containing 10% (v/v) heat-inactivated FBS, 100 units/ml penicillin, and 100 mg/ml streptomycin. The cells were incubated at 37°C under a 5% CO_2_ and 90% relative humidity (RH) atmosphere.

### 4.5 *In vitro* anticancer activity (MTT assay)

Stock solutions were prepared by diluting target compounds in DMSO at concentrations (1.25, 2.5, 5, and 10 mM). The stock solution was then diluted (1.25, 2.5, 5, and 10 μM) with culture medium, and the concentration of DMSO was controlled not to exceed 0.1%.

Place the cells in a 96-well plate at an appropriate density to ensure exponential growth (1 × 10^4^–1.2×10^4^ cells per well) throughout the experiment, and then allow the cells to adhere for 24 h. The cells were then treated with continuous concentrations of each compound (1.25, 2.5, 5, and 10 μM) for 48 h. After 48 h of incubation, 15 μl of MTT solution was added to each well, and the final concentration was 2 mg/ml. Then incubate for another 4 h. After incubation, remove the MTT solution and add 150 ml of DMSO to each well for staining. Shake vigorously for 10 min at room temperature to ensure complete dissolution. The optical density (OD) was read on a microplate reader (ELx 800, BioTek, Highland Park, Winooski, VT, United States) with a wavelength of 492 nm, and then the data was analyzed.

### 4.6 Analysis for cell cycle by flow cytometry

Inoculate HCT116 cells in a 6-well plate (5.0 × 105 cells per well) and incubate at 37°C for 24 h. The exponentially growing cells were then incubated with compound **E** at 10 and 30 μM. After 24 h, untreated cells (control) or cells treated with compound **E** were centrifuged at 1,000 rpm for 15 min, and then fixed in 70% ethanol at 20°C for at least 24 h. The cells were then resuspended in phosphate buffered saline (PBS) containing 0.1 mg/ml RNase A and 5 mg/ml PI. The DNA content of cells used for cell cycle distribution analysis was measured by flow cytometry, and analyzed using FACSalibur flow cytometer and Cell Quest software (Becton-Dickinson, Franklin Lakes, NJ, United States).

### 4.7 Colony formation assay

Exponentially growing HCT116 cells (600 cells per well) were plated on 6-well plates with Roswell Park Memorial Institute (RPMI) 1,640 medium containing 10% fetal bovine serum (FBS). After incubating for 12 h, replace with fresh medium. Then, the cells were treated with different concentrations of compound **E** (2.5, 5 and 10 μM) dissolved in DMSO. Some cells only use DMSO as a negative control. Then incubate for another 7 days. Finally, the cells were fixed with 95% ethanol for 25 min, stained with 0.1% crystal violet for 15 min at room temperature, and then washed with PBS until the colonies were completely removed.

### 4.8 Apoptosis analysis

Apoptosis detection kit (Invitrogen, Eugene, OR, United State) was used to detect cell apoptosis. Culture the cells in a 6-well plate (5.0 × 10^5^ cells per well) and incubate at 37°C for 24 h. The exponentially growing cells were then incubated with compound **E** at 10 and 30 μM. After 24 h of incubation, the cells were collected, washed twice with PBS, and washed with 1× binding buffer at room temperature for 30 min in the dark. A FACSCalibur flow cytometer with Cell Quest software (Bectone Dickinson, Franklin Lakes, NJ, United State) was used to count apoptotic cells.

### 4.9 Western blot analysis

HCT116 cells were cultured with different concentrations of compound **E** for 24 h. Then, the floating cells were collected and washed twice with ice-cold PBS. Add cold RIPA buffer. After the cells were lysed on ice for 20 min, the lysate was centrifuged at 12,000 rpm for 15 min at 4°C. The BCA method determines the protein concentration. Separated by SDS/PAGE electrophoresis and transferred to a nitrocellulose filter membrane for Western blot analysis. The membrane was blocked with PBS containing 5% skim milk at room temperature for 2 h. Then incubate the membrane with primary antibodies against Bax, Bcl-2, p-IκB, IκB, p-NF-κB p65, NF-κB p65, p-ERK, ERK, p-JNK, JNK, p-p38, and p38 protein and β-actin at 4°C with gentle rotation overnight. Next, incubate the membrane with the fluorescent secondary antibody for 2 h. The protein is detected by electrochemiluminescence (Bio-Rad, CA, United State).

## Data Availability

The original contributions presented in the study are included in the article/Supplementary Material, further inquiries can be directed to the corresponding authors.
