# Supplementary material for: Synthesis and evaluation of anticancer activity of quillaic acid derivatives: A cell cycle arrest and apoptosis inducer through NF-κB and MAPK pathways
Source: Front Chem. 2022 Sep 7;10:951713. doi: 10.3389/fchem.2022.951713 (PMC9490060; doi:10.3389/fchem.2022.951713)
Supplement: Supplementary file 1 [file DataSheet1.docx]

**Synthesis and Evaluation of Anticancer Activity of Quillaic Acid Derivatives: A cell cycle arrest and apoptosis inducer through NF-κB and MAPK pathways**

Xing Huang^1^, Chang-Hao Zhang^1^, Hao Deng, Dan Wu, Hong-Yan Guo, Jung Joon Lee, Fen-Er Chen, Qing-Kun Shen, Li-Li Jin*, Zhe-Shan Quan*

Key Laboratory of Natural Medicines of the Changbai Mountain, Affifiliated Ministry of Education, College of Pharmacy, Yanbian University, Yanji, Jilin, 133002, China

*Corresponding author:

E-mail: [zsquan@ybu.edu.cn](mailto:cmjin@ybu.edu.cn) (Z. S. Quan).

E-mail: jinlili@ybu.edu.cn (L. L. Jin).

^1^These authors contributed equally

**Table 1.** log P values for compounds **A1-A10**, **B1-B6**, **C1-C**6, **D** and **E.**

| **Compound** | log P | **Compound** | log P |
| --- | --- | --- | --- |
| **A1** | **7.63** | **B3** | **7.57** |
| **A2** | **7.95** | **B4** | **7.71** |
| **A3** | **8.52** | **B5** | **7.13** |
| **A4** | **7.17** | **B6** | **7.44** |
| **A5** | **8.67** | **C1** | **7.84** |
| **A6** | **7.86** | **C2** | **8.10** |
| **A7** | **7.54** | **C3** | **8.67** |
| **A8** | **7.14** | **C4** | **9.30** |
| **A9** | **8.13** | **C5** | **7.83** |
| **A10** | **7.74** | **C6** | **7.15** |
| **B1** | **7.02** | **D** | **7.71** |
| **B2** | **7.17** | **E** | **8.22** |

Stability of Compound **E** under Pharmacological Experimental Conditions

**Experimental method:** Samples **A-E** were prepared and detected by liquid phase. Details are as follows.

**A**: The purity of compound **E** was detected by liquid phase, and the peak time of compound E was judged.

**B**: Liquid detection RPMI 1640 medium

**C**: Compound **E** was dissolved in the RPMI 1640 medium and detected by liquid phase.

**D**: Compound **E** was dissolved in the RPMI 1640 medium and placed in the incubator for 24 hours before detection by liquid phase.

**E**: Compound **E** was dissolved in the RPMI 1640 medium and placed in the incubator for 48 hours before detection by liquid phase.

The liquid test results are shown in **Figure A-E**.

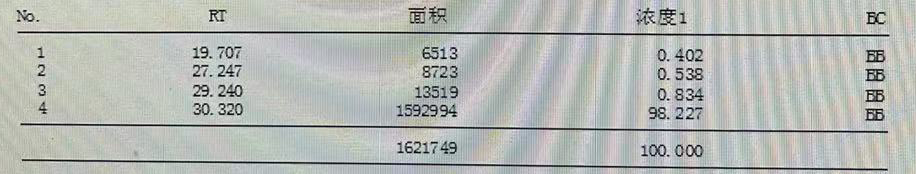


**Figure A**. Purity and peak time of compound **E**.

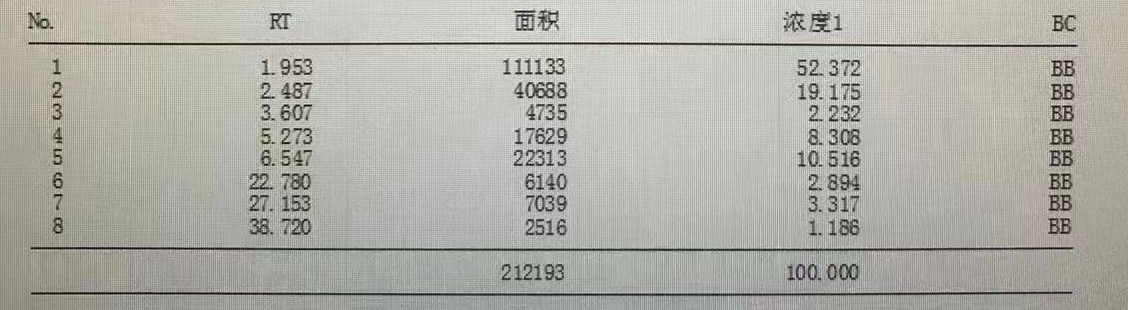


**Figure B**. HPLC chart of RPMI 1640 medium.

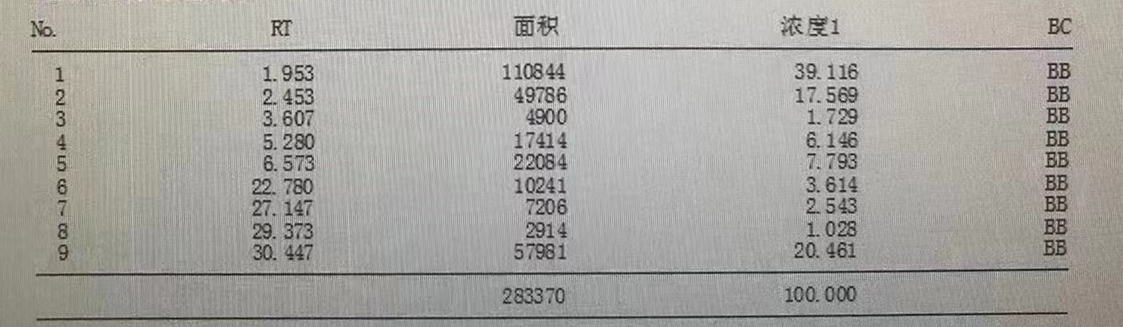


**Figure C**. HPLC profile of compound **E** in RPMI 1640 medium (Check directly/**0** hours).

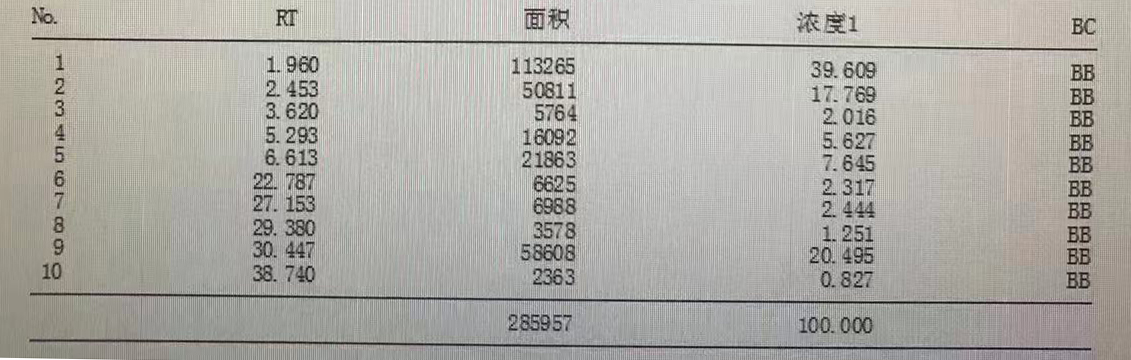


**Figure D**. HPLC profile of compound **E** in RPMI 1640 medium (Test after **24** hours in incubator).

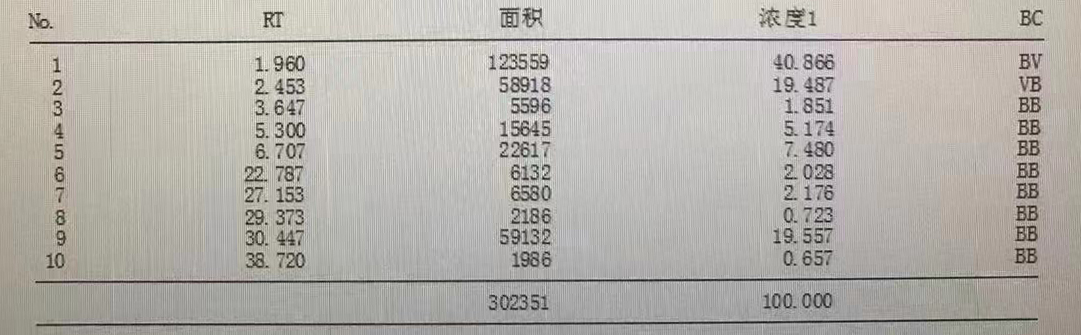


**Figure E**. HPLC profile of compound **E** in RPMI 1640 medium (Test after **48** hours in incubator).

**Analyze:**

As can be seen from **Figure A**, compound **E** will peak at 30.320 minutes.

As can be seen from **Figure B**, the RPMI 1640 medium does not have a peak of 30.320. **Figures A** and **B** show that the peak obtained at 30.320 minutes is compound **E**.

**Figure C**, **Figure D** and **Figure E** have corresponding peaks at 30.447 minutes, and this peak is judged to be the peak of compound **E**. The contents are 20.461%, 20.495% and 19.557% in sequence. Therefore, it can be judged that the content of compound E in **Figure C**, **Figure D** and **Figure E** is basically the same.

**Conclusion:** Compound **E** has high stability under pharmacological experimental conditions.


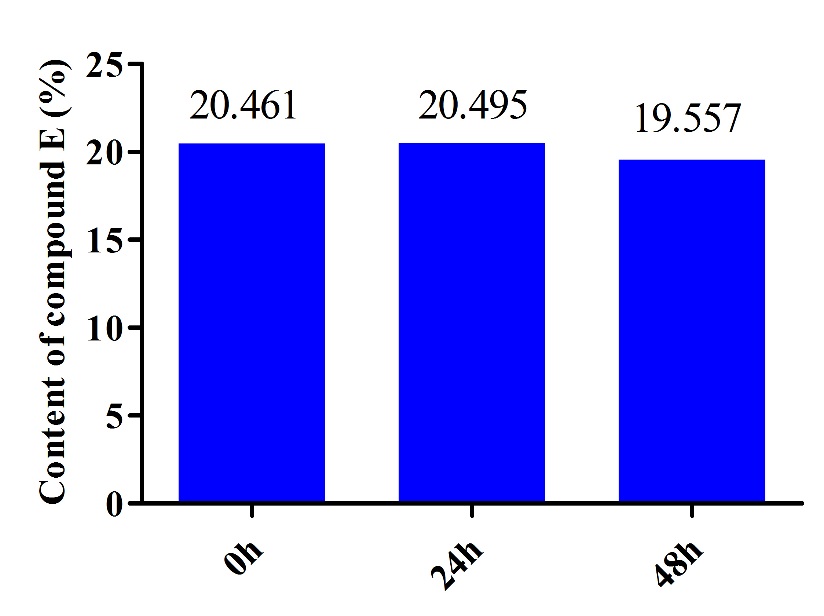


**Figure F**. The content of compound **E** at different times (0h, 24h, 48h).


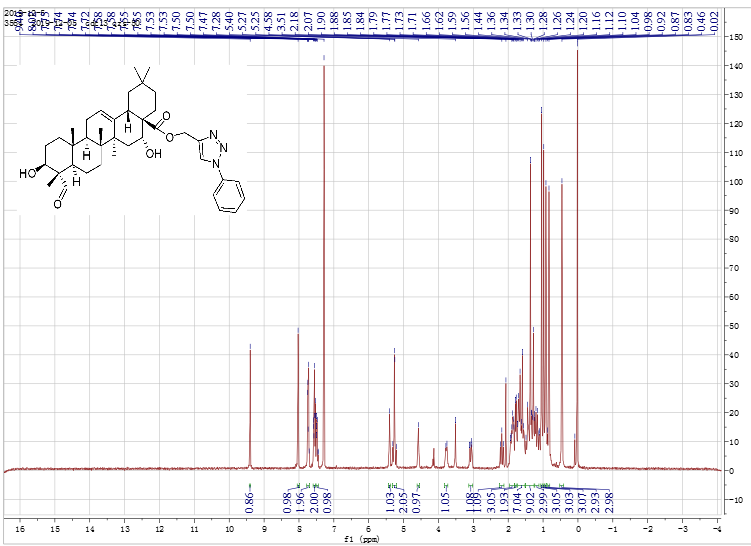


**Figure 1.** ^1^H-NMR spectrum of compound **A1.**

(1-phenyl-1H-1,2,3-triazol-4-yl)methyl (4aR,5R,6aS,6bR,8aR,9S,10S,12aR,12bR,14bS)-9-formyl-5,10-dihydroxy-2,2,6a,6b,9,12a-hexamethyl-1,3,4,5,6,6a,6b,7,8,8a,9,10,11,12,12a,12b,13,14b-octadecahydropicene-4a(2H)-carboxylate (**A1**)


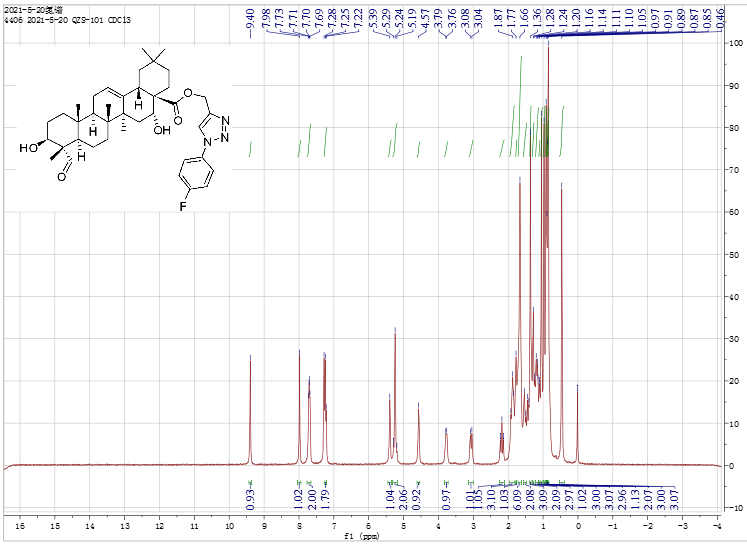


**Figure 2.** ^1^H-NMR spectrum of compound **A2.**

(1-(4-fluorophenyl)-1H-1,2,3-triazol-4-yl)methyl (4aR,5R,6aS,6bR,8aR,9S,10S,12aR,12bR,14bS)-9-formyl-5,10-dihydroxy-2,2,6a,6b,9,12a-hexamethyl-1,3,4,5,6,6a,6b,7,8,8a,9,10,11,12,12a,12b,13,14b-octadecahydropicene-4a(2H)-carboxylate (**A2**)


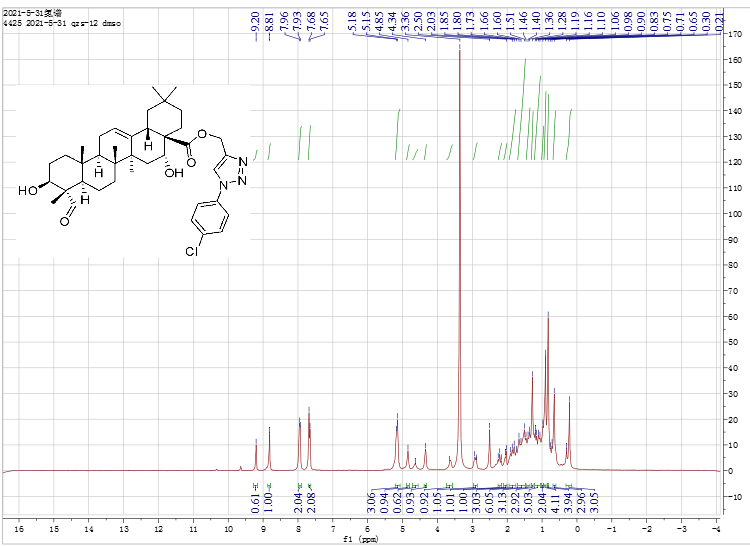


**Figure 3.** ^1^H-NMR spectrum of compound **A3.**

(1-(4-chlorophenyl)-1H-1,2,3-triazol-4-yl)methyl (4aR,5R,6aS,6bR,8aR,9S,10S,12aR,12bR,14bS)-9-formyl-5,10-dihydroxy-2,2,6a,6b,9,12a-hexamethyl-1,3,4,5,6,6a,6b,7,8,8a,9,10,11,12,12a,12b,13,14b-octadecahydropicene-4a(2H)-carboxylate (**A3**)


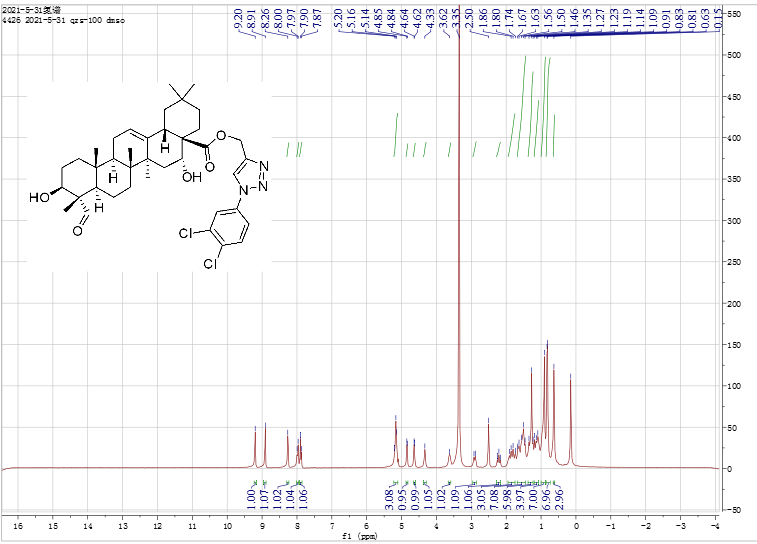


**Figure 4.** ^1^H-NMR spectrum of compound **A4.**

(1-(3,4-dichlorophenyl)-1H-1,2,3-triazol-4-yl)methyl (4aR,5R,6aS,6bR,8aR,9S,10S,12aR,12bR,14bS)-9-formyl-5,10-dihydroxy-2,2,6a,6b,9,12a-hexamethyl-1,3,4,5,6,6a,6b,7,8,8a,9,10,11,12,12a,12b,13,14b-octadecahydropicene-4a(2H)-carboxylate (**A4**)


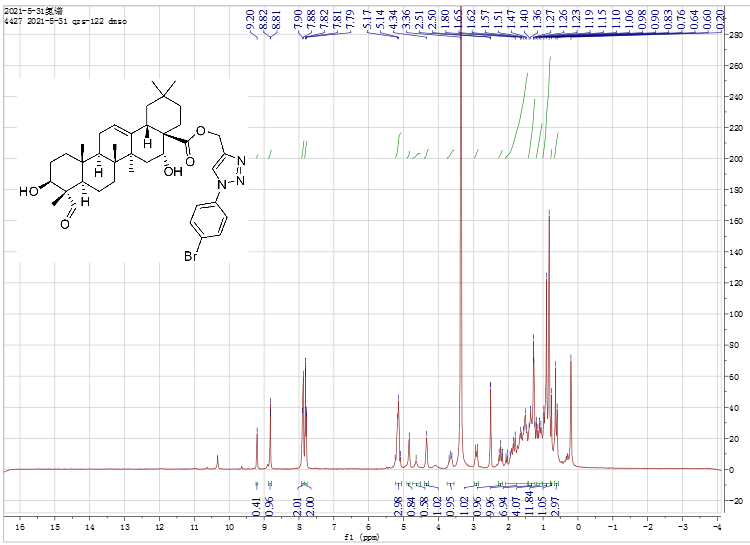


**Figure 5.** ^1^H-NMR spectrum of compound **A5.**

(1-(4-bromophenyl)-1H-1,2,3-triazol-4-yl)methyl (4aR,5R,6aS,6bR,8aR,9S,10S,12aR,12bR,14bS)-9-formyl-5,10-dihydroxy-2,2,6a,6b,9,12a-hexamethyl-1,3,4,5,6,6a,6b,7,8,8a,9,10,11,12,12a,12b,13,14b-octadecahydropicene-4a(2H)-carboxylate (**A5**)


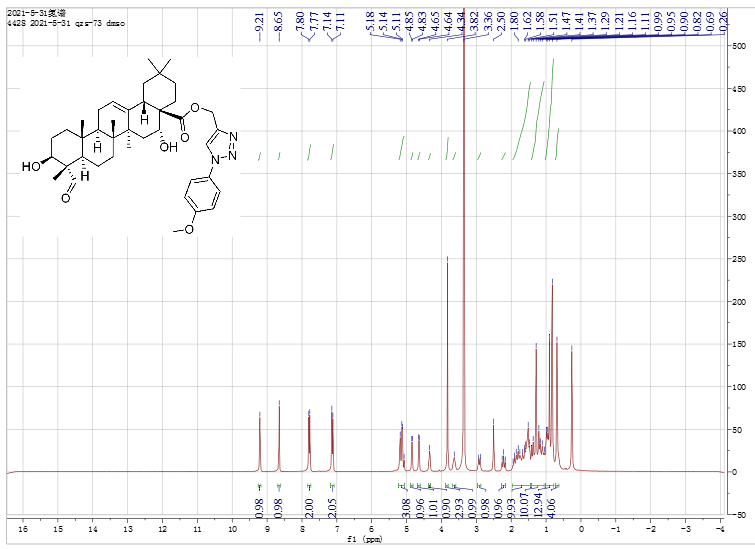


**Figure 6.** ^1^H-NMR spectrum of compound **A6.**

(1-(4-methoxyphenyl)-1H-1,2,3-triazol-4-yl)methyl (4aR,5R,6aS,6bR,8aR,9S,10S,12aR,12bR,14bS)-9-formyl-5,10-dihydroxy-2,2,6a,6b,9,12a-hexamethyl-1,3,4,5,6,6a,6b,7,8,8a,9,10,11,12,12a,12b,13,14b-octadecahydropicene-4a(2H)-carboxylate (**A6**)


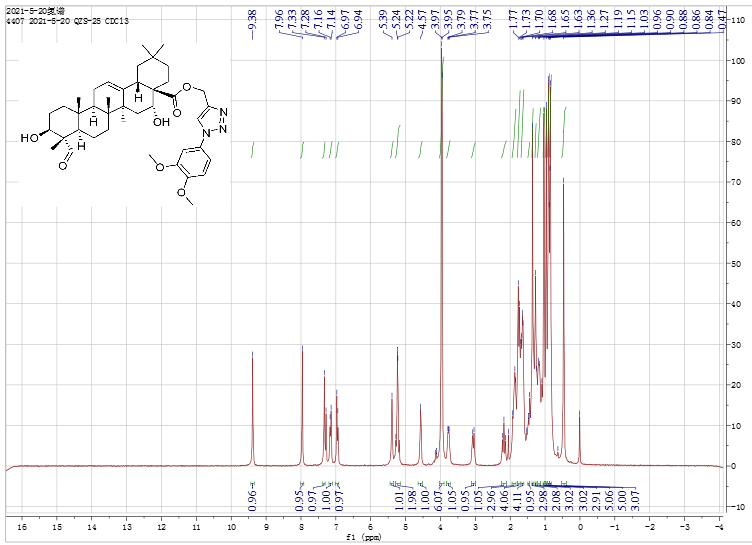


**Figure 7.** ^1^H-NMR spectrum of compound **A7.**

(1-(3,4-dimethoxyphenyl)-1H-1,2,3-triazol-4-yl)methyl (4aR,5R,6aS,6bR,8aR,9S,10S,12aR,12bR,14bS)-9-formyl-5,10-dihydroxy-2,2,6a,6b,9,12a-hexamethyl-1,3,4,5,6,6a,6b,7,8,8a,9,10,11,12,12a,12b,13,14b-octadecahydropicene-4a(2H)-carboxylate (**A7**)


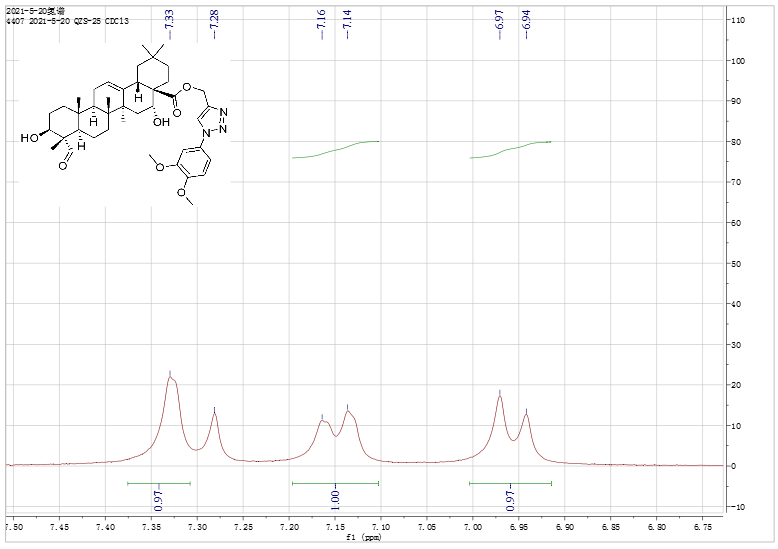


**Figure 7B.** ^1^H-NMR zoomed-in spectrum of compound **A7.** Range 7.33-6.94.

The peak 7.28 for CDCl_3_ was not integrated.


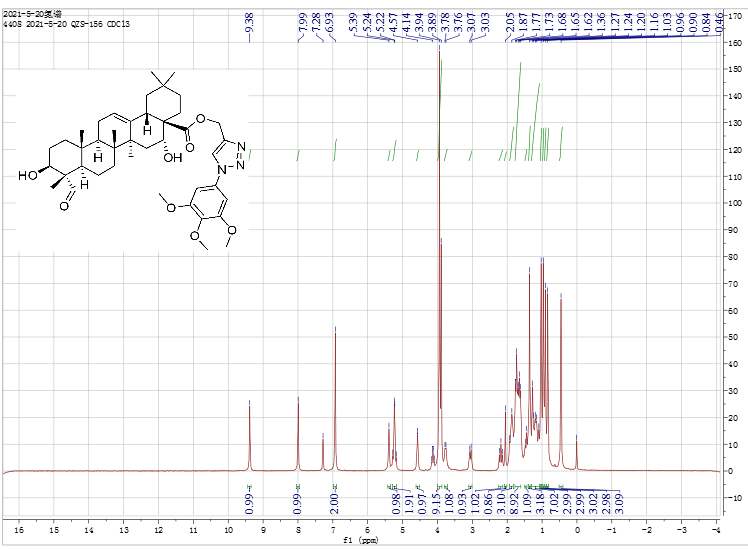


**Figure 8.** ^1^H-NMR spectrum of compound **A8.**

(1-(3,4,5-trimethoxyphenyl)-1H-1,2,3-triazol-4-yl)methyl (4aR,5R,6aS,6bR,8aR,9S,10S,12aR,12bR,14bS)-9-formyl-5,10-dihydroxy 2,2,6a,6b,9,12a-hexamethyl-1,3,4,5,6,6a,6b,7,8,8a,9,10,11,12,12a,12b,13,14b octadecahydropicene-4a(2H)-carboxylate (**A8**)


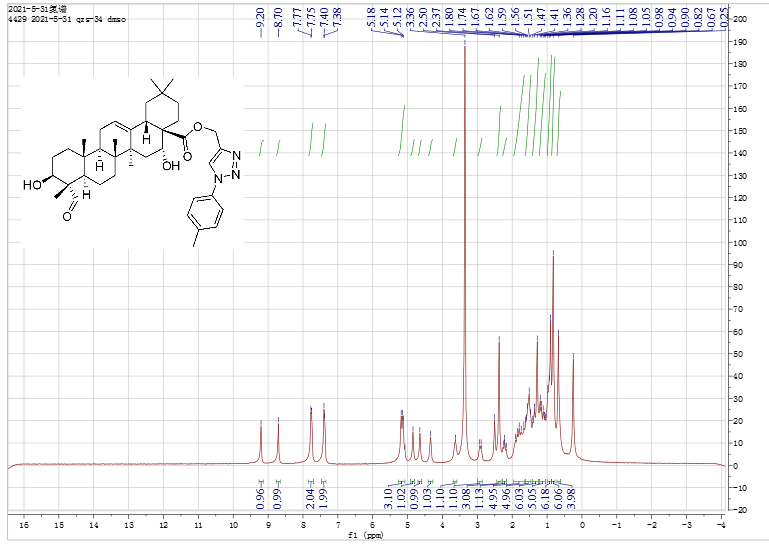


**Figure 9.** ^1^H-NMR spectrum of compound **A9.**

(1-(p-tolyl)-1H-1,2,3-triazol-4-yl)methyl (4aR,5R,6aS,6bR,8aR,9S,10S,12aR,12bR,14bS)-9-formyl-5,10-dihydroxy-2,2,6a,6b,9,12a-hexamethyl-1,3,4,5,6,6a,6b,7,8,8a,9,10,11,12,12a,12b,13,14b-octadecahydropicene-4a(2H)-carboxylate (**A9**)


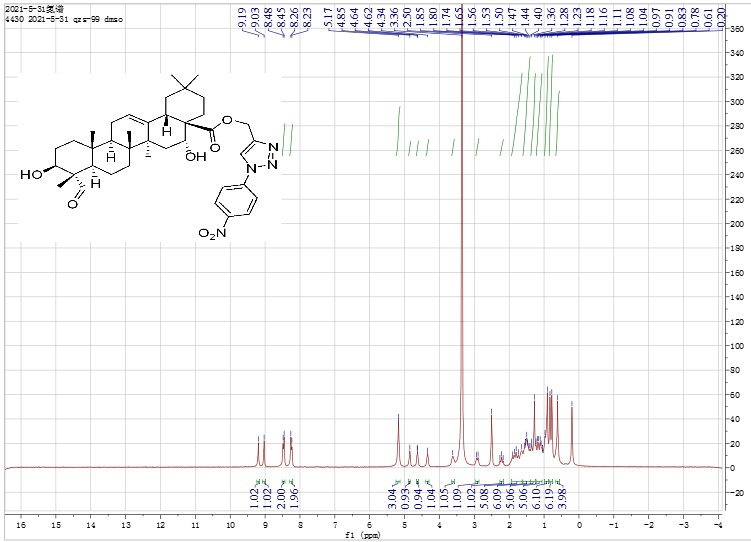


**Figure 10.** ^1^H-NMR spectrum of compound **A10.**

(1-(4-nitrophenyl)-1H-1,2,3-triazol-4-yl)methyl (4aR,5R,6aS,6bR,8aR,9S,10S,12aR,12bR,14bS)-9-formyl-5,10-dihydroxy-2,2,6a,6b,9,12a-hexamethyl-1,3,4,5,6,6a,6b,7,8,8a,9,10,11,12,12a,12b,13,14b-octadecahydropicene-4a(2H)-carboxylate (**A10**)


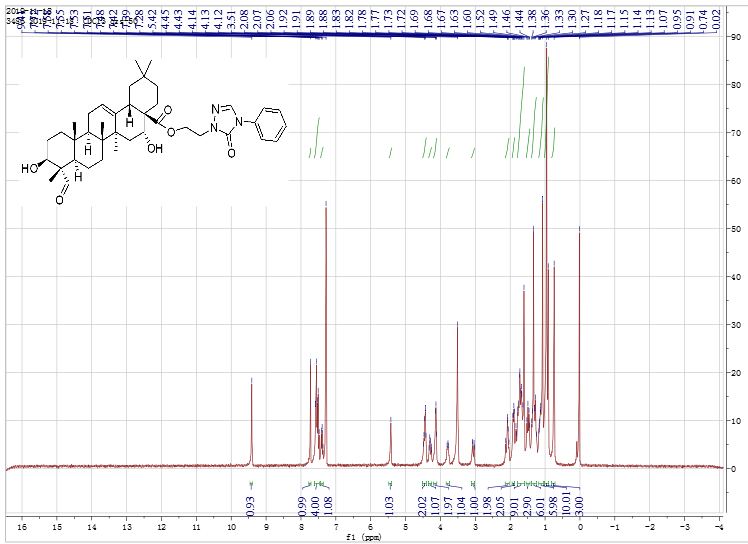


**Figure 11.** ^1^H-NMR spectrum of compound **C1.**

2-(5-oxo-4-phenyl-4,5-dihydro-1H-1,2,4-triazol-1-yl)ethyl (4aR,5R,6aS,6bR,8aR,9S,10S,12aR,12bR,14bS)-9-formyl-5,10-dihydroxy-2,2,6a,6b,9,12a-hexamethyl-1,3,4,5,6,6a,6b,7,8,8a,9,10,11,12,12a,12b,13,14b-octadecahydropicene-4a(2H)-carboxylate (**C1**)


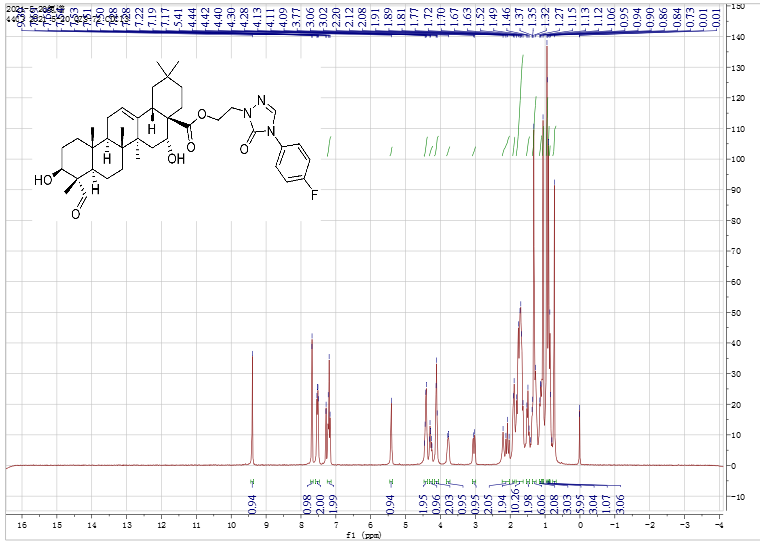


**Figure 12.** ^1^H-NMR spectrum of compound **C2.**

2-(4-(4-fluorophenyl)-5-oxo-4,5-dihydro-1H-1,2,4-triazol-1-yl)ethyl (4aR,5R,6aS,6bR,8aR,9S,10S,12aR,12bR,14bS)-9-formyl-5,10-dihydroxy-2,2,6a,6b,9,12a-hexamethyl-1,3,4,5,6,6a,6b,7,8,8a,9,10,11,12,12a,12b,13,14b-octadecahydropicene-4a(2H)-carboxylate (**C2**)


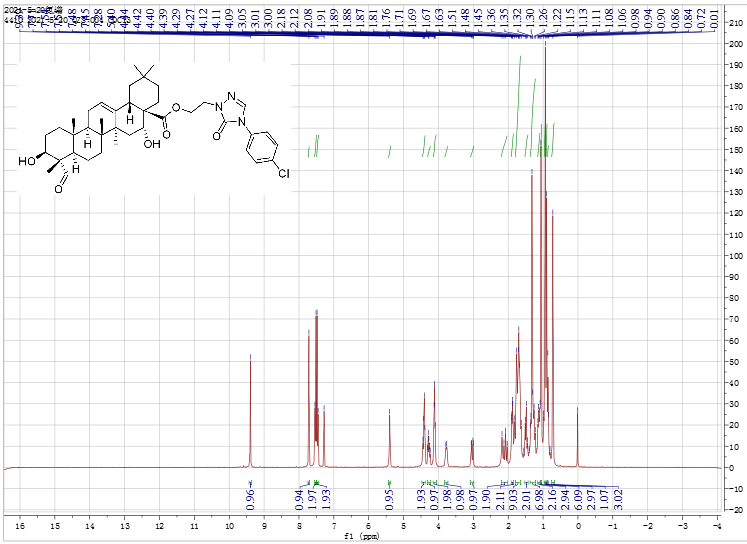


**Figure 13.** ^1^H-NMR spectrum of compound **C3.**

2-(4-(4-chlorophenyl)-5-oxo-4,5-dihydro-1H-1,2,4-triazol-1-yl)ethyl (4aR,5R,6aS,6bR,8aR,9S,10S,12aR,12bR,14bS)-9-formyl-5,10-dihydroxy-2,2,6a,6b,9,12a-hexamethyl-1,3,4,5,6,6a,6b,7,8,8a,9,10,11,12,12a,12b,13,14b-octadecahydropicene-4a(2H)-carboxylate (**C3**)


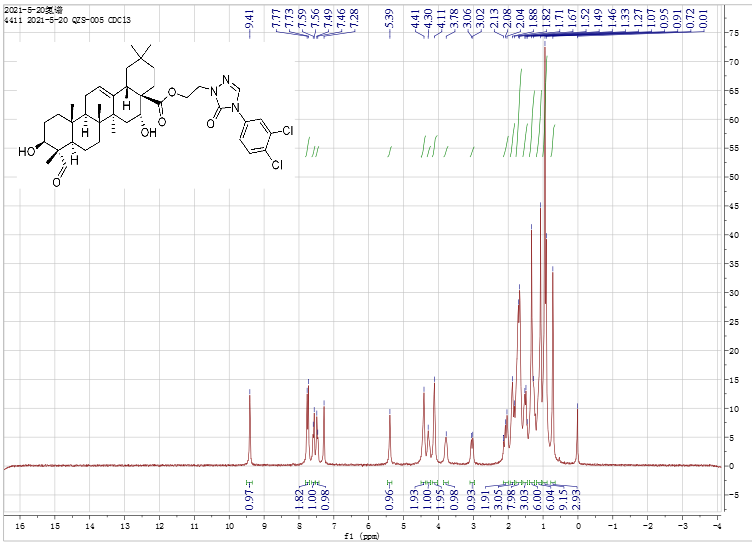


**Figure 14.** ^1^H-NMR spectrum of compound **C4.**

2-(4-(3,4-dichlorophenyl)-5-oxo-4,5-dihydro-1H-1,2,4-triazol-1-yl)ethyl (4aR,5R,6aS,6bR,8aR,9S,10S,12aR,12bR,14bS)-9-formyl-5,10-dihydroxy-2,2,6a,6b,9,12a-hexamethyl-1,3,4,5,6,6a,6b,7,8,8a,9,10,11,12,12a,12b,13,14b-octadecahydropicene-4a(2H)-carboxylate (**C4**)


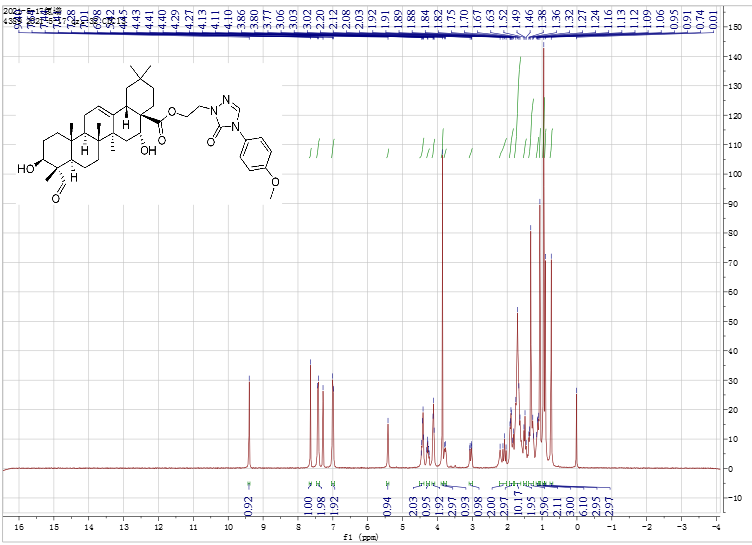


**Figure 15.** ^1^H-NMR spectrum of compound **C5.**

2-(4-(4-methoxyphenyl)-5-oxo-4,5-dihydro-1H-1,2,4-triazol-1-yl)ethyl (4aR,5R,6aS,6bR,8aR,9S,10S,12aR,12bR,14bS)-9-formyl-5,10-dihydroxy-2,2,6a,6b,9,12a-hexamethyl-1,3,4,5,6,6a,6b,7,8,8a,9,10,11,12,12a,12b,13,14b-octadecahydropicene-4a(2H)-carboxylate (**C5**)


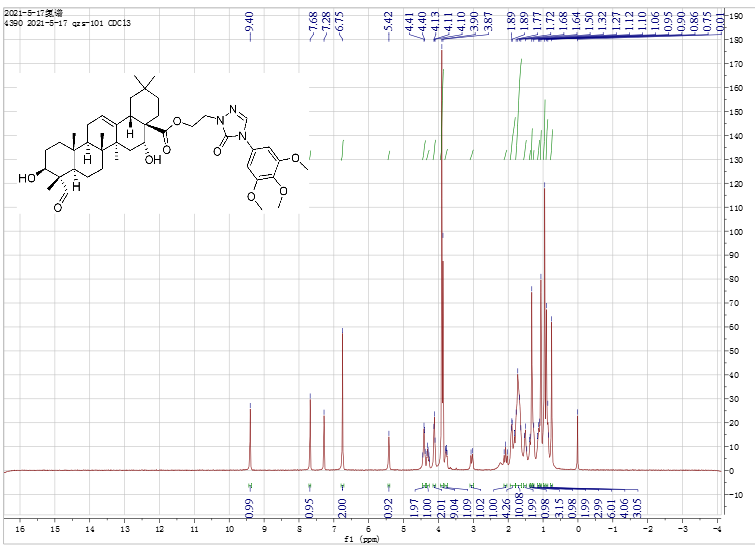


**Figure 16.** ^1^H-NMR spectrum of compound **C6.**

2-(5-oxo-4-(3,4,5-trimethoxyphenyl)-4,5-dihydro-1H-1,2,4-triazol-1-yl)ethyl (4aR,5R,6aS,6bR,8aR,9S,10S,12aR,12bR,14bS)-9-formyl-5,10-dihydroxy-2,2,6a,6b,9,12a-hexamethyl-1,3,4,5,6,6a,6b,7,8,8a,9,10,11,12,12a,12b,13,14b-octadecahydropicene-4a(2H)-carboxylate (**C6**)


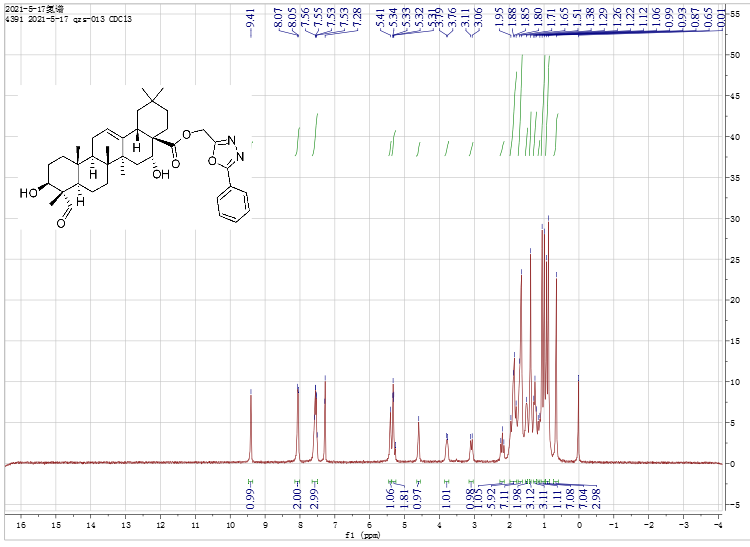


**Figure 17.** ^1^H-NMR spectrum of compound **B1.**

(5-phenyl-1,3,4-oxadiazol-2-yl)methyl (4aR,5R,6aS,6bR,8aR,9S,10S,12aR,12bR,14bS)-9-formyl-5,10-dihydroxy-2,2,6a,6b,9,12a-hexamethyl-1,3,4,5,6,6a,6b,7,8,8a,9,10,11,12,12a,12b,13,14b-octadecahydropicene-4a(2H)-carboxylate (**B1**)


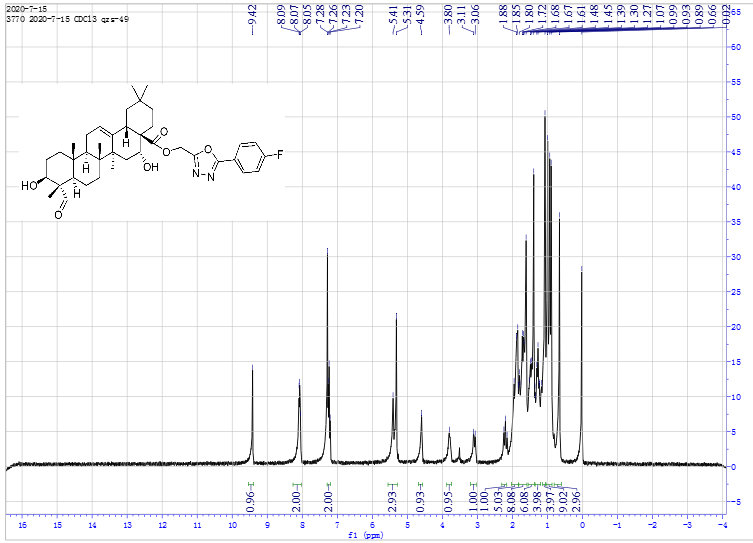


**Figure 18.** ^1^H-NMR spectrum of compound **B2.**

(5-(4-fluorophenyl)-1,3,4-oxadiazol-2-yl)methyl (4aR,5R,6aS,6bR,8aR,9S,10S,12aR,12bR,14bS)-9-formyl-5,10-dihydroxy-2,2,6a,6b,9,12a-hexamethyl-1,3,4,5,6,6a,6b,7,8,8a,9,10,11,12,12a,12b,13,14b-octadecahydropicene-4a(2H)-carboxylate (**B2**)


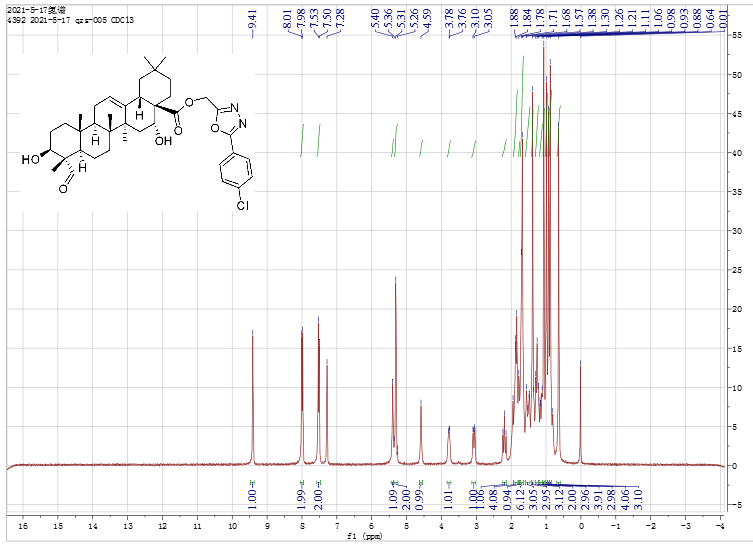


**Figure 19.** ^1^H-NMR spectrum of compound **B3.**

(5-(4-chlorophenyl)-1,3,4-oxadiazol-2-yl)methyl (4aR,5R,6aS,6bR,8aR,9S,10S,12aR,12bR,14bS)-9-formyl-5,10-dihydroxy-2,2,6a,6b,9,12a-hexamethyl-1,3,4,5,6,6a,6b,7,8,8a,9,10,11,12,12a,12b,13,14b-octadecahydropicene-4a(2H)-carboxylate (**B3**)


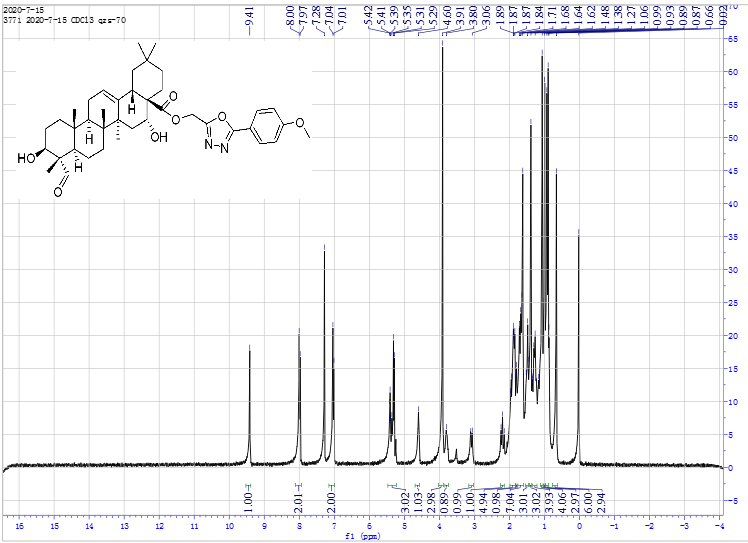


**Figure 20.** ^1^H-NMR spectrum of compound **B4.**

(5-(4-methoxyphenyl)-1,3,4-oxadiazol-2-yl)methyl (4aR,5R,6aS,6bR,8aR,9S,10S,12aR,12bR,14bS)-9-formyl-5,10-dihydroxy-2,2,6a,6b,9,12a-hexamethyl-1,3,4,5,6,6a,6b,7,8,8a,9,10,11,12,12a,12b,13,14b-octadecahydropicene-4a(2H)-carboxylate (**B4**)


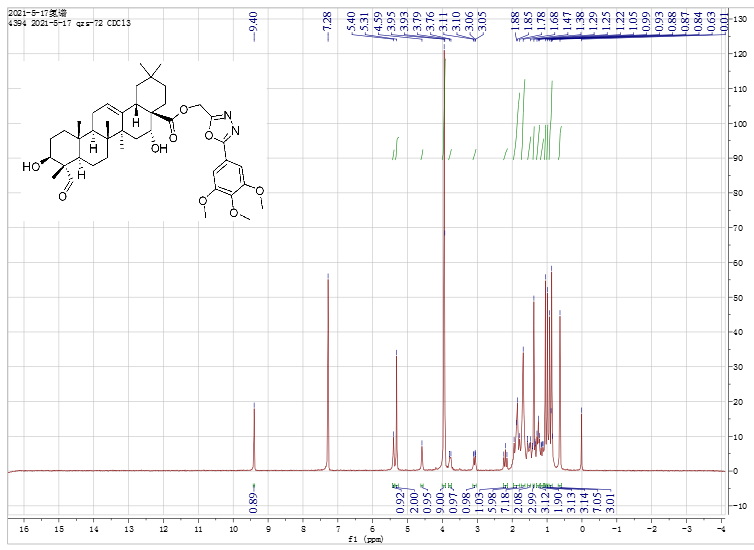


**Figure 21.** ^1^H-NMR spectrum of compound **B5.**

(5-(3,4,5-trimethoxyphenyl)-1,3,4-oxadiazol-2-yl)methyl (4aR,5R,6aS,6bR,8aR,9S,10S,12aR,12bR,14bS)-9-formyl-5,10-dihydroxy-2,2,6a,6b,9,12a-hexamethyl-1,3,4,5,6,6a,6b,7,8,8a,9,10,11,12,12a,12b,13,14b-octadecahydropicene-4a(2H)-carboxylate (**B5**)


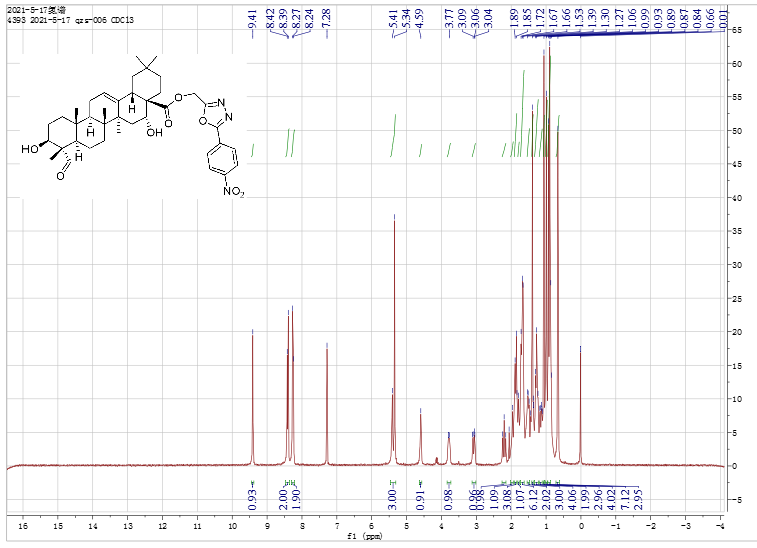


**Figure 22.** ^1^H-NMR spectrum of compound **B6.**

(5-(4-nitrophenyl)-1,3,4-oxadiazol-2-yl)methyl (4aR,5R,6aS,6bR,8aR,9S,10S,12aR,12bR,14bS)-9-formyl-5,10-dihydroxy-2,2,6a,6b,9,12a-hexamethyl-1,3,4,5,6,6a,6b,7,8,8a,9,10,11,12,12a,12b,13,14b-octadecahydropicene-4a(2H)-carboxylate (**B6**)


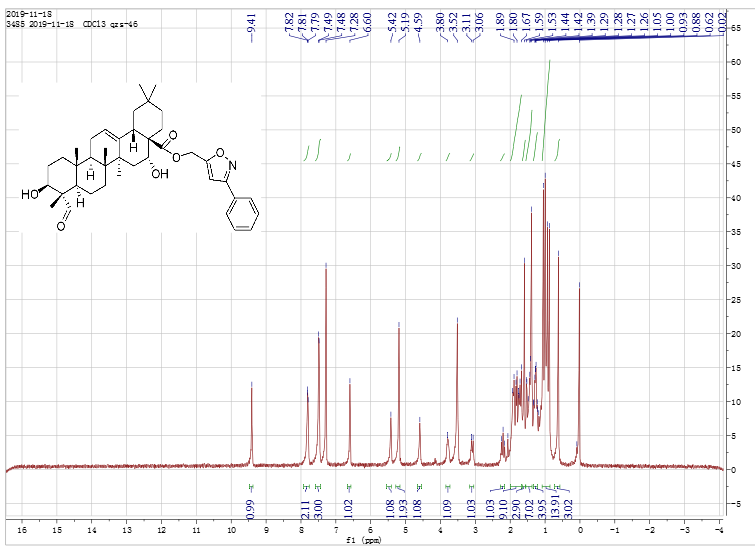


**Figure 23.** ^1^H-NMR spectrum of compound **D.**

(3-phenylisoxazol-5-yl)methyl (4aR,5R,6aS,6bR,8aR,9S,10S,12aR,12bR,14bS)-9-formyl-5,10-dihydroxy-2,2,6a,6b,9,12a-hexamethyl-1,3,4,5,6,6a,6b,7,8,8a,9,10,11,12,12a,12b,13,14b-octadecahydropicene-4a(2H)-carboxylate (**D**)


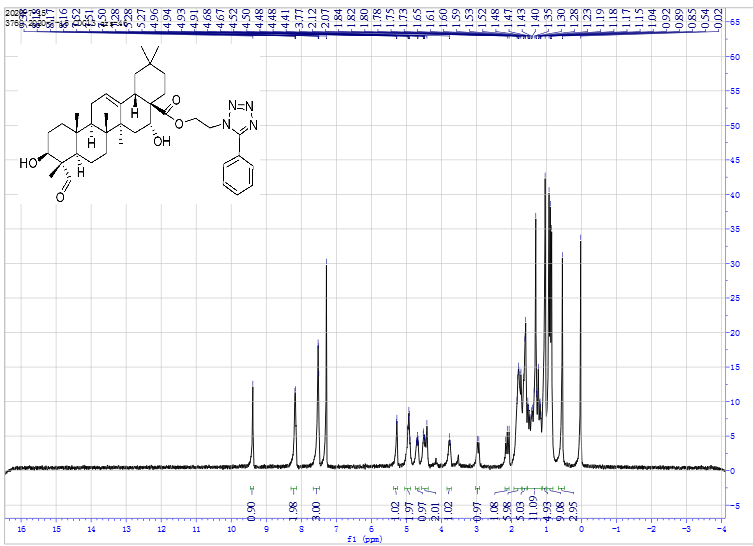


**Figure 24.** ^1^H-NMR spectrum of compound **F.**

2-(5-phenyl-1H-tetrazol-1-yl)ethyl (4aR,5R,6aS,6bR,8aR,9S,10S,12aR,12bR,14bS)-9-formyl-5,10-dihydroxy-2,2,6a,6b,9,12a-hexamethyl-1,3,4,5,6,6a,6b,7,8,8a,9,10,11,12,12a,12b,13,14b-octadecahydropicene-4a(2H)-carboxylate (**F**)


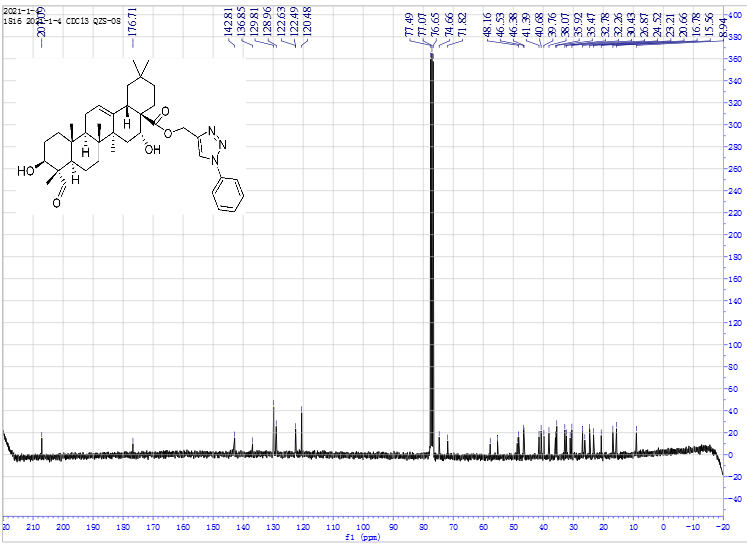


**Figure 25.** ^13^H-NMR spectrum of compound **A1.**

(1-phenyl-1H-1,2,3-triazol-4-yl)methyl (4aR,5R,6aS,6bR,8aR,9S,10S,12aR,12bR,14bS)-9-formyl-5,10-dihydroxy-2,2,6a,6b,9,12a-hexamethyl-1,3,4,5,6,6a,6b,7,8,8a,9,10,11,12,12a,12b,13,14b-octadecahydropicene-4a(2H)-carboxylate (**A1**)


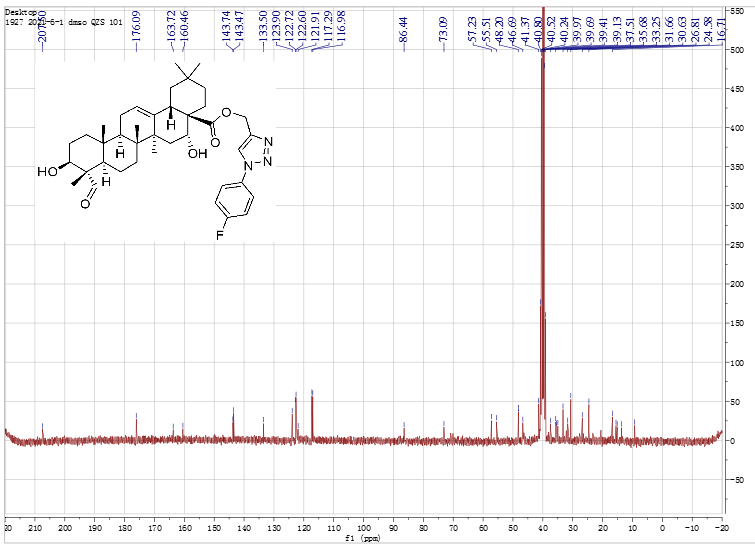


**Figure 26.** ^13^H-NMR spectrum of compound **A2.**

(1-(4-fluorophenyl)-1H-1,2,3-triazol-4-yl)methyl (4aR,5R,6aS,6bR,8aR,9S,10S,12aR,12bR,14bS)-9-formyl-5,10-dihydroxy-2,2,6a,6b,9,12a-hexamethyl-1,3,4,5,6,6a,6b,7,8,8a,9,10,11,12,12a,12b,13,14b-octadecahydropicene-4a(2H)-carboxylate (**A2**)


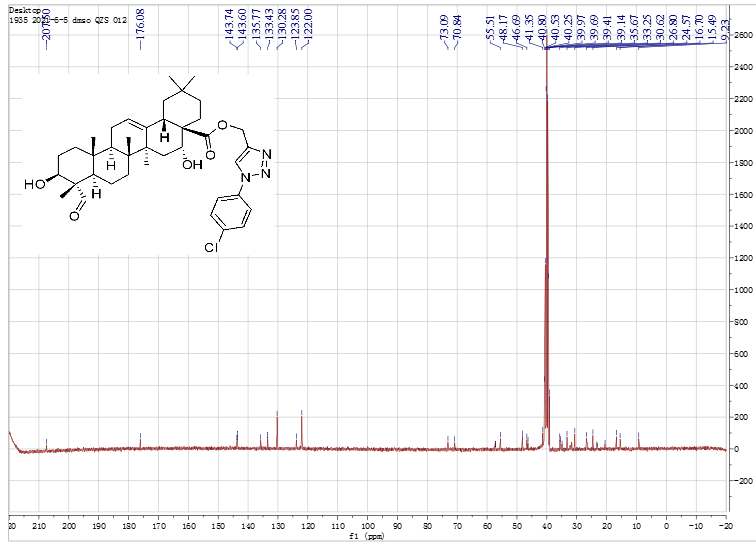


**Figure 27.** ^13^H-NMR spectrum of compound **A3.**

(1-(4-chlorophenyl)-1H-1,2,3-triazol-4-yl)methyl (4aR,5R,6aS,6bR,8aR,9S,10S,12aR,12bR,14bS)-9-formyl-5,10-dihydroxy-2,2,6a,6b,9,12a-hexamethyl-1,3,4,5,6,6a,6b,7,8,8a,9,10,11,12,12a,12b,13,14b-octadecahydropicene-4a(2H)-carboxylate (**A3**)


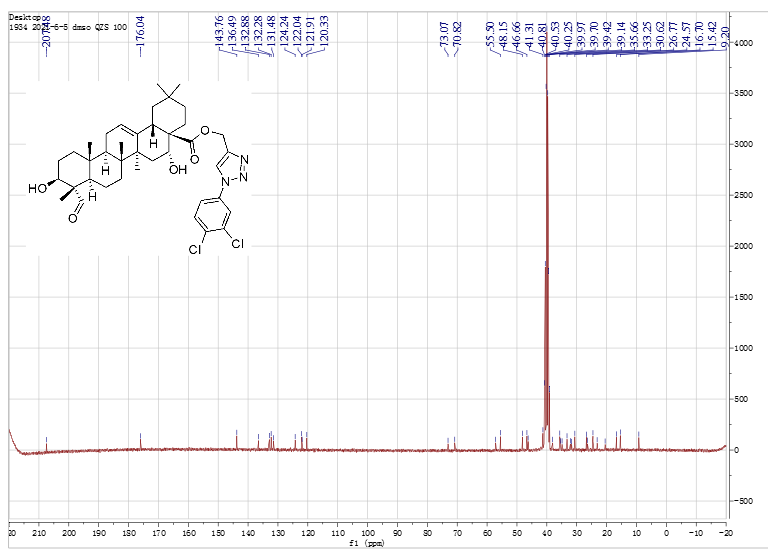


**Figure 28.** ^13^H-NMR spectrum of compound **A4.**

(1-(3,4-dichlorophenyl)-1H-1,2,3-triazol-4-yl)methyl (4aR,5R,6aS,6bR,8aR,9S,10S,12aR,12bR,14bS)-9-formyl-5,10-dihydroxy-2,2,6a,6b,9,12a-hexamethyl-1,3,4,5,6,6a,6b,7,8,8a,9,10,11,12,12a,12b,13,14b-octadecahydropicene-4a(2H)-carboxylate (**A4**)


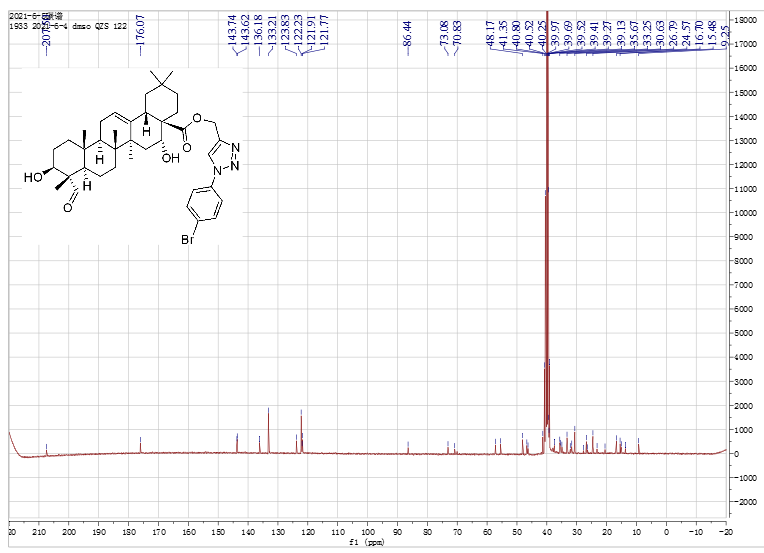


**Figure 29.** ^13^H-NMR spectrum of compound **A5.**

(1-(4-bromophenyl)-1H-1,2,3-triazol-4-yl)methyl (4aR,5R,6aS,6bR,8aR,9S,10S,12aR,12bR,14bS)-9-formyl-5,10-dihydroxy-2,2,6a,6b,9,12a-hexamethyl-1,3,4,5,6,6a,6b,7,8,8a,9,10,11,12,12a,12b,13,14b-octadecahydropicene-4a(2H)-carboxylate (**A5**)


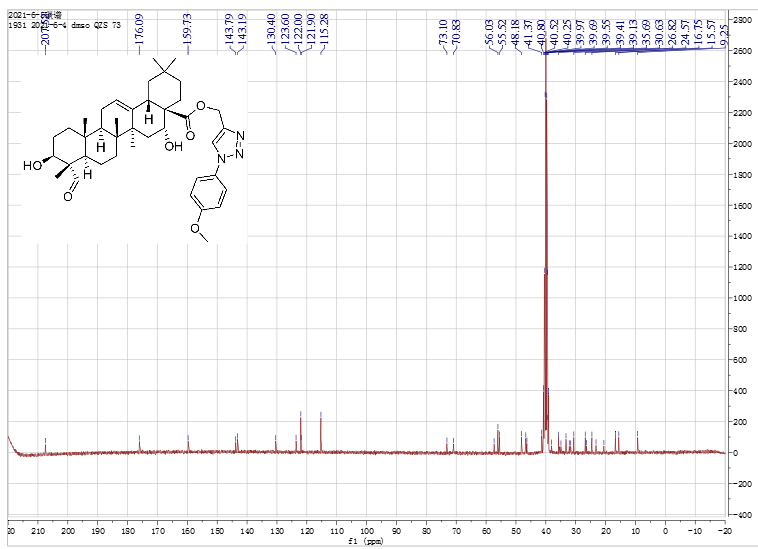


**Figure 30.** ^13^H-NMR spectrum of compound **A6.**

(1-(4-methoxyphenyl)-1H-1,2,3-triazol-4-yl)methyl (4aR,5R,6aS,6bR,8aR,9S,10S,12aR,12bR,14bS)-9-formyl-5,10-dihydroxy-2,2,6a,6b,9,12a-hexamethyl-1,3,4,5,6,6a,6b,7,8,8a,9,10,11,12,12a,12b,13,14b-octadecahydropicene-4a(2H)-carboxylate (**A6**)


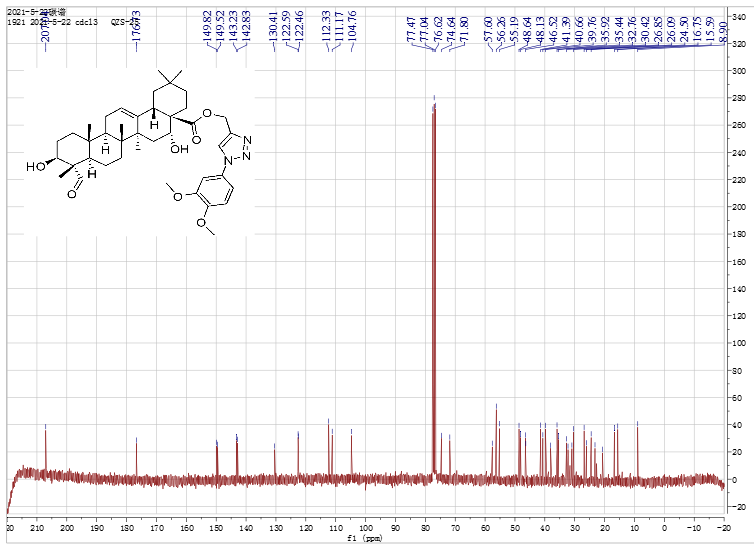


**Figure 31.** ^13^H-NMR spectrum of compound **A7.**

(1-(3,4-dimethoxyphenyl)-1H-1,2,3-triazol-4-yl)methyl (4aR,5R,6aS,6bR,8aR,9S,10S,12aR,12bR,14bS)-9-formyl-5,10-dihydroxy-2,2,6a,6b,9,12a-hexamethyl-1,3,4,5,6,6a,6b,7,8,8a,9,10,11,12,12a,12b,13,14b-octadecahydropicene-4a(2H)-carboxylate (**A7**)


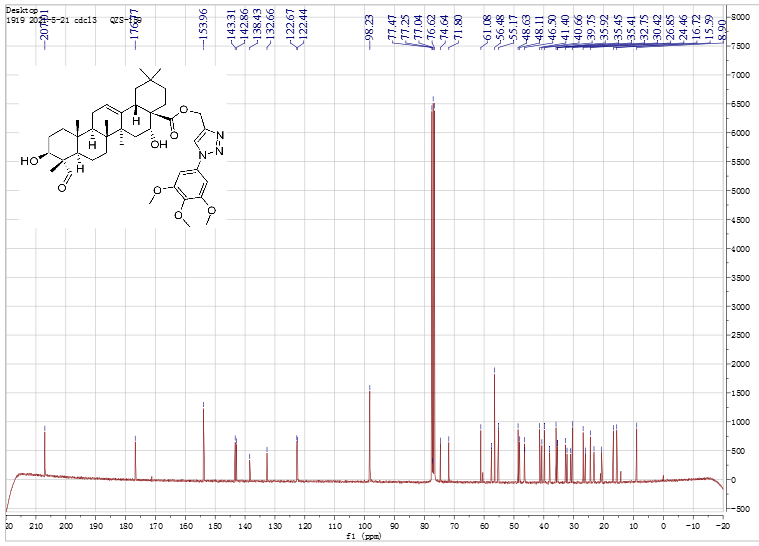


**Figure 32.** ^13^H-NMR spectrum of compound **A8.**

(1-(3,4,5-trimethoxyphenyl)-1H-1,2,3-triazol-4-yl)methyl (4aR,5R,6aS,6bR,8aR,9S,10S,12aR,12bR,14bS)-9-formyl-5,10-dihydroxy 2,2,6a,6b,9,12a-hexamethyl-1,3,4,5,6,6a,6b,7,8,8a,9,10,11,12,12a,12b,13,14b octadecahydropicene-4a(2H)-carboxylate (**A8**)


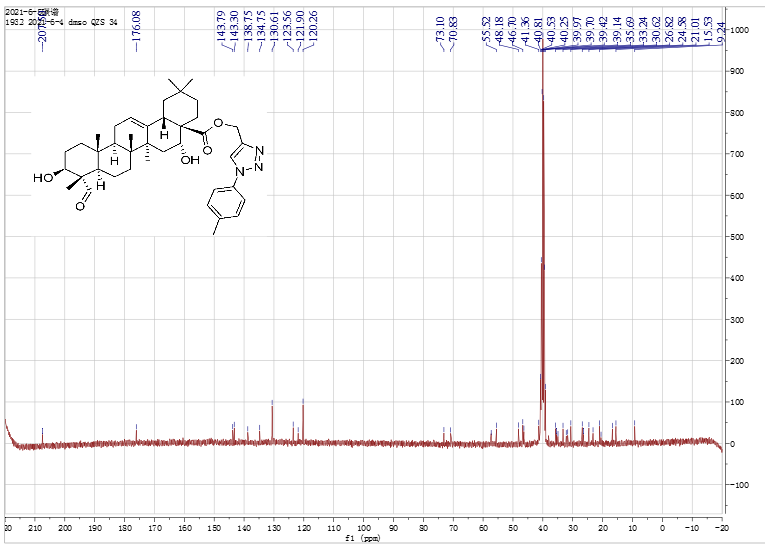


**Figure 33.** ^13^H-NMR spectrum of compound **A9.**

(1-(p-tolyl)-1H-1,2,3-triazol-4-yl)methyl (4aR,5R,6aS,6bR,8aR,9S,10S,12aR,12bR,14bS)-9-formyl-5,10-dihydroxy-2,2,6a,6b,9,12a-hexamethyl-1,3,4,5,6,6a,6b,7,8,8a,9,10,11,12,12a,12b,13,14b-octadecahydropicene-4a(2H)-carboxylate (**A9**)


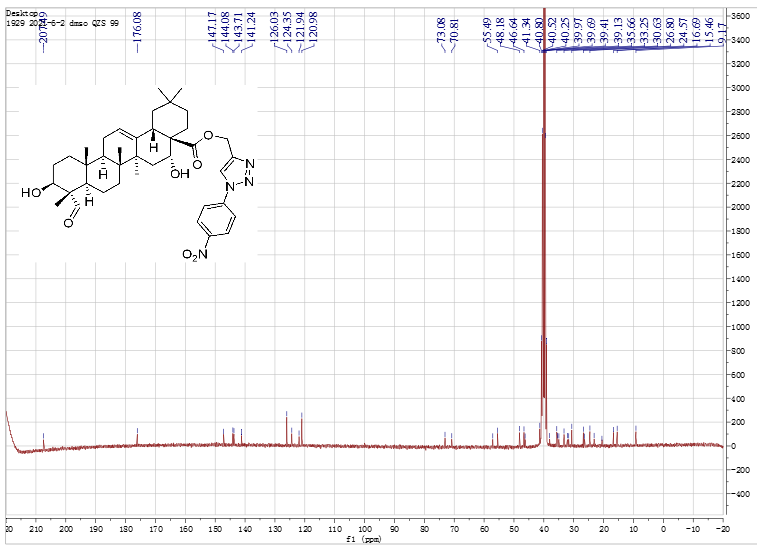


**Figure 34.** ^13^H-NMR spectrum of compound **A10.**

(1-(4-nitrophenyl)-1H-1,2,3-triazol-4-yl)methyl (4aR,5R,6aS,6bR,8aR,9S,10S,12aR,12bR,14bS)-9-formyl-5,10-dihydroxy-2,2,6a,6b,9,12a-hexamethyl-1,3,4,5,6,6a,6b,7,8,8a,9,10,11,12,12a,12b,13,14b-octadecahydropicene-4a(2H)-carboxylate (**A10**)


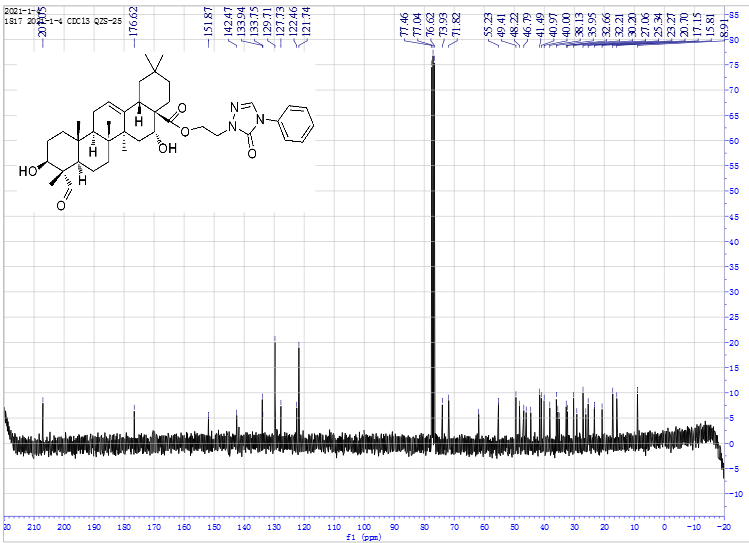


**Figure 35.** ^13^H-NMR spectrum of compound **C1.**

2-(5-oxo-4-phenyl-4,5-dihydro-1H-1,2,4-triazol-1-yl)ethyl (4aR,5R,6aS,6bR,8aR,9S,10S,12aR,12bR,14bS)-9-formyl-5,10-dihydroxy-2,2,6a,6b,9,12a-hexamethyl-1,3,4,5,6,6a,6b,7,8,8a,9,10,11,12,12a,12b,13,14b-octadecahydropicene-4a(2H)-carboxylate (**C1**)


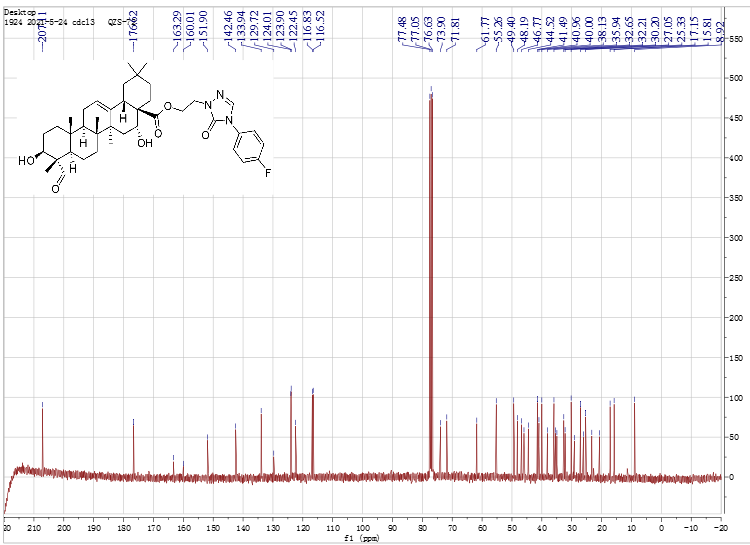


**Figure 36.** ^13^H-NMR spectrum of compound **C2.**

2-(4-(4-fluorophenyl)-5-oxo-4,5-dihydro-1H-1,2,4-triazol-1-yl)ethyl (4aR,5R,6aS,6bR,8aR,9S,10S,12aR,12bR,14bS)-9-formyl-5,10-dihydroxy-2,2,6a,6b,9,12a-hexamethyl-1,3,4,5,6,6a,6b,7,8,8a,9,10,11,12,12a,12b,13,14b-octadecahydropicene-4a(2H)-carboxylate (**C2**)


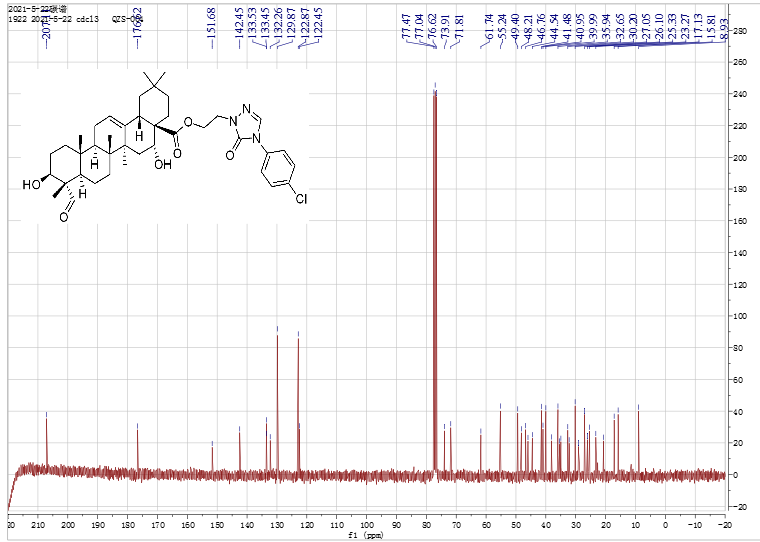


**Figure 37.** ^13^H-NMR spectrum of compound **C3.**

2-(4-(4-chlorophenyl)-5-oxo-4,5-dihydro-1H-1,2,4-triazol-1-yl)ethyl (4aR,5R,6aS,6bR,8aR,9S,10S,12aR,12bR,14bS)-9-formyl-5,10-dihydroxy-2,2,6a,6b,9,12a-hexamethyl-1,3,4,5,6,6a,6b,7,8,8a,9,10,11,12,12a,12b,13,14b-octadecahydropicene-4a(2H)-carboxylate (**C3**)


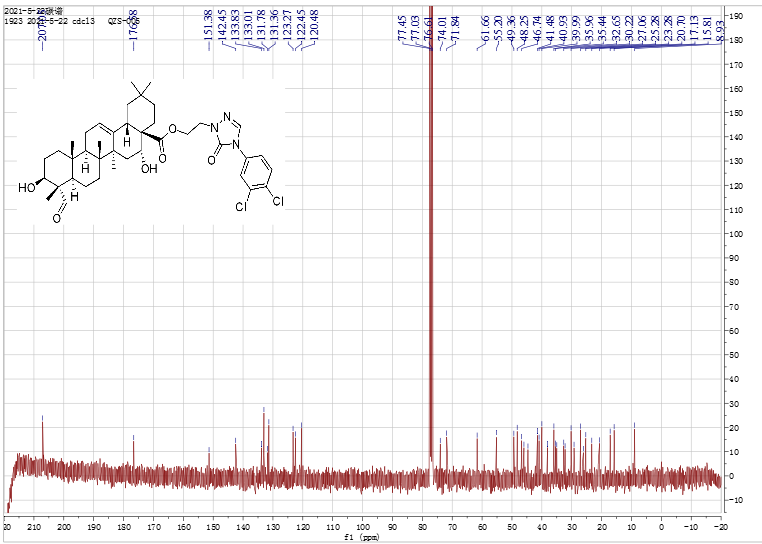


**Figure 38.** ^13^H-NMR spectrum of compound **C4.**

2-(4-(3,4-dichlorophenyl)-5-oxo-4,5-dihydro-1H-1,2,4-triazol-1-yl)ethyl (4aR,5R,6aS,6bR,8aR,9S,10S,12aR,12bR,14bS)-9-formyl-5,10-dihydroxy-2,2,6a,6b,9,12a-hexamethyl-1,3,4,5,6,6a,6b,7,8,8a,9,10,11,12,12a,12b,13,14b-octadecahydropicene-4a(2H)-carboxylate (**C4**)


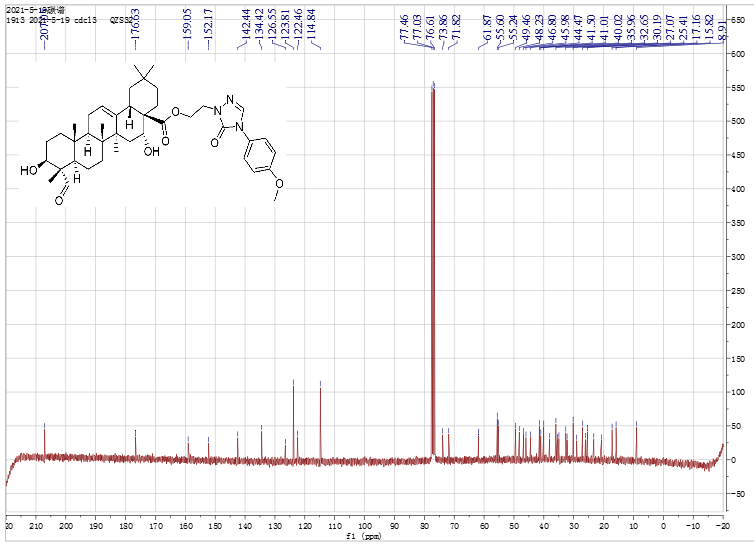


**Figure 39.** ^13^H-NMR spectrum of compound **C5.**

2-(4-(4-methoxyphenyl)-5-oxo-4,5-dihydro-1H-1,2,4-triazol-1-yl)ethyl (4aR,5R,6aS,6bR,8aR,9S,10S,12aR,12bR,14bS)-9-formyl-5,10-dihydroxy-2,2,6a,6b,9,12a-hexamethyl-1,3,4,5,6,6a,6b,7,8,8a,9,10,11,12,12a,12b,13,14b-octadecahydropicene-4a(2H)-carboxylate (**C5**)


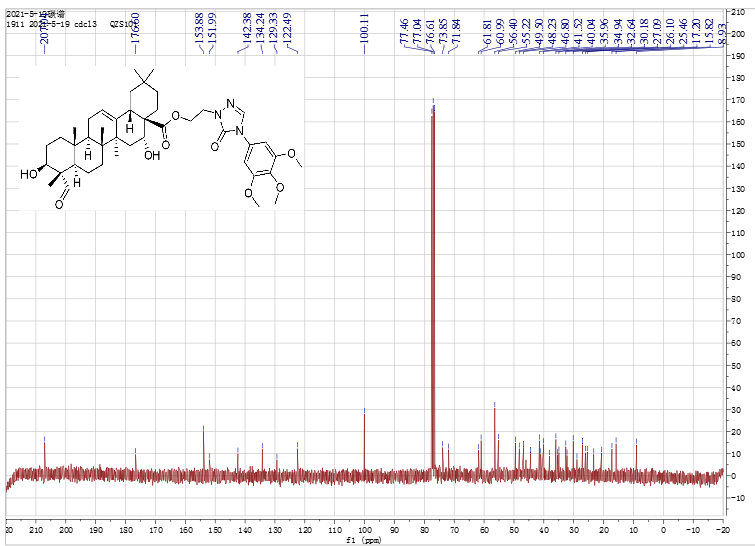


**Figure 40.** ^13^H-NMR spectrum of compound **C6.**

2-(5-oxo-4-(3,4,5-trimethoxyphenyl)-4,5-dihydro-1H-1,2,4-triazol-1-yl)ethyl (4aR,5R,6aS,6bR,8aR,9S,10S,12aR,12bR,14bS)-9-formyl-5,10-dihydroxy-2,2,6a,6b,9,12a-hexamethyl-1,3,4,5,6,6a,6b,7,8,8a,9,10,11,12,12a,12b,13,14b-octadecahydropicene-4a(2H)-carboxylate (**C6**)


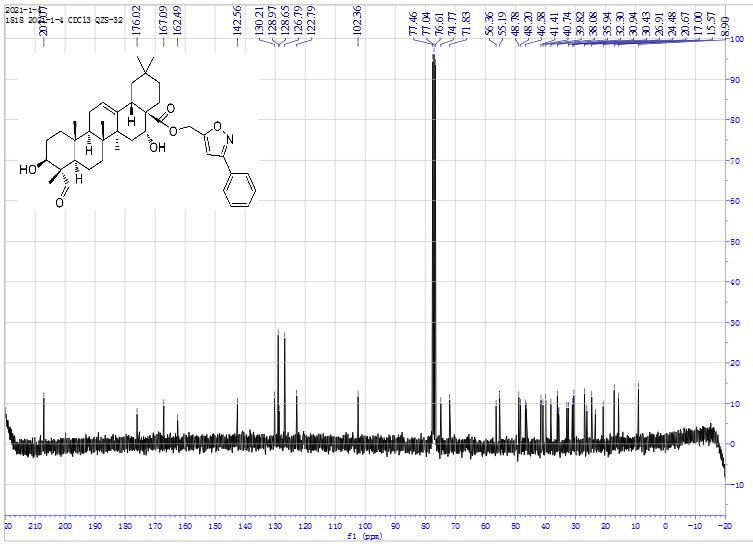


**Figure 47.** ^13^H-NMR spectrum of compound **D.**

(3-phenylisoxazol-5-yl)methyl (4aR,5R,6aS,6bR,8aR,9S,10S,12aR,12bR,14bS)-9-formyl-5,10-dihydroxy-2,2,6a,6b,9,12a-hexamethyl-1,3,4,5,6,6a,6b,7,8,8a,9,10,11,12,12a,12b,13,14b-octadecahydropicene-4a(2H)-carboxylate (**D**)


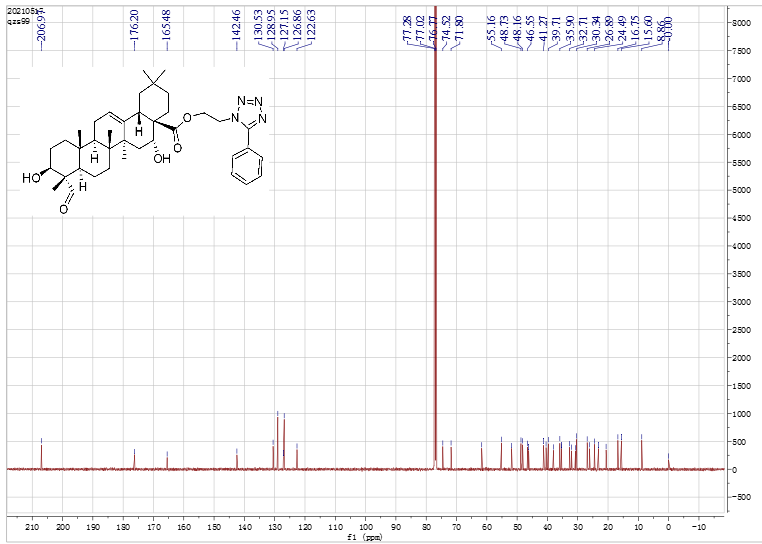


**Figure 48.** ^13^H-NMR spectrum of compound **F.**

2-(5-phenyl-1H-tetrazol-1-yl)ethyl (4aR,5R,6aS,6bR,8aR,9S,10S,12aR,12bR,14bS)-9-formyl-5,10-dihydroxy-2,2,6a,6b,9,12a-hexamethyl-1,3,4,5,6,6a,6b,7,8,8a,9,10,11,12,12a,12b,13,14b-octadecahydropicene-4a(2H)-carboxylate (**F**)

**Figure 49.** HRMS spectrum of compound **A1.**

(1-phenyl-1H-1,2,3-triazol-4-yl)methyl (4aR,5R,6aS,6bR,8aR,9S,10S,12aR,12bR,14bS)-9-formyl-5,10-dihydroxy-2,2,6a,6b,9,12a-hexamethyl-1,3,4,5,6,6a,6b,7,8,8a,9,10,11,12,12a,12b,13,14b-octadecahydropicene-4a(2H)-carboxylate (**A1**)

**Figure 50.** HRMS spectrum of compound **A2.**

(1-(4-fluorophenyl)-1H-1,2,3-triazol-4-yl)methyl (4aR,5R,6aS,6bR,8aR,9S,10S,12aR,12bR,14bS)-9-formyl-5,10-dihydroxy-2,2,6a,6b,9,12a-hexamethyl-1,3,4,5,6,6a,6b,7,8,8a,9,10,11,12,12a,12b,13,14b-octadecahydropicene-4a(2H)-carboxylate (**A2**)

**Figure 51.** HRMS spectrum of compound **A3.**

(1-(4-chlorophenyl)-1H-1,2,3-triazol-4-yl)methyl (4aR,5R,6aS,6bR,8aR,9S,10S,12aR,12bR,14bS)-9-formyl-5,10-dihydroxy-2,2,6a,6b,9,12a-hexamethyl-1,3,4,5,6,6a,6b,7,8,8a,9,10,11,12,12a,12b,13,14b-octadecahydropicene-4a(2H)-carboxylate (**A3**)

**Figure 52.** HRMS spectrum of compound **A4.**

(1-(3,4-dichlorophenyl)-1H-1,2,3-triazol-4-yl)methyl (4aR,5R,6aS,6bR,8aR,9S,10S,12aR,12bR,14bS)-9-formyl-5,10-dihydroxy-2,2,6a,6b,9,12a-hexamethyl-1,3,4,5,6,6a,6b,7,8,8a,9,10,11,12,12a,12b,13,14b-octadecahydropicene-4a(2H)-carboxylate (**A4**)

**Figure 53.** HRMS spectrum of compound **A5.**

(1-(4-bromophenyl)-1H-1,2,3-triazol-4-yl)methyl (4aR,5R,6aS,6bR,8aR,9S,10S,12aR,12bR,14bS)-9-formyl-5,10-dihydroxy-2,2,6a,6b,9,12a-hexamethyl-1,3,4,5,6,6a,6b,7,8,8a,9,10,11,12,12a,12b,13,14b-octadecahydropicene-4a(2H)-carboxylate (**A5**)

**Figure 54.** HRMS spectrum of compound **A6.**

(1-(4-methoxyphenyl)-1H-1,2,3-triazol-4-yl)methyl (4aR,5R,6aS,6bR,8aR,9S,10S,12aR,12bR,14bS)-9-formyl-5,10-dihydroxy-2,2,6a,6b,9,12a-hexamethyl-1,3,4,5,6,6a,6b,7,8,8a,9,10,11,12,12a,12b,13,14b-octadecahydropicene-4a(2H)-carboxylate (**A6**)

**Figure 55.** HRMS spectrum of compound **A71.**

(1-(3,4-dimethoxyphenyl)-1H-1,2,3-triazol-4-yl)methyl (4aR,5R,6aS,6bR,8aR,9S,10S,12aR,12bR,14bS)-9-formyl-5,10-dihydroxy-2,2,6a,6b,9,12a-hexamethyl-1,3,4,5,6,6a,6b,7,8,8a,9,10,11,12,12a,12b,13,14b-octadecahydropicene-4a(2H)-carboxylate (**A7**)

**Figure 56.** HRMS spectrum of compound **A8.**

(1-(3,4,5-trimethoxyphenyl)-1H-1,2,3-triazol-4-yl)methyl (4aR,5R,6aS,6bR,8aR,9S,10S,12aR,12bR,14bS)-9-formyl-5,10-dihydroxy 2,2,6a,6b,9,12a-hexamethyl-1,3,4,5,6,6a,6b,7,8,8a,9,10,11,12,12a,12b,13,14b octadecahydropicene-4a(2H)-carboxylate (**A8**)

**Figure 57.** HRMS spectrum of compound **A9.**

(1-(p-tolyl)-1H-1,2,3-triazol-4-yl)methyl (4aR,5R,6aS,6bR,8aR,9S,10S,12aR,12bR,14bS)-9-formyl-5,10-dihydroxy-2,2,6a,6b,9,12a-hexamethyl-1,3,4,5,6,6a,6b,7,8,8a,9,10,11,12,12a,12b,13,14b-octadecahydropicene-4a(2H)-carboxylate (**A9**)

**Figure 58.** HRMS spectrum of compound **A10.**

(1-(4-nitrophenyl)-1H-1,2,3-triazol-4-yl)methyl (4aR,5R,6aS,6bR,8aR,9S,10S,12aR,12bR,14bS)-9-formyl-5,10-dihydroxy-2,2,6a,6b,9,12a-hexamethyl-1,3,4,5,6,6a,6b,7,8,8a,9,10,11,12,12a,12b,13,14b-octadecahydropicene-4a(2H)-carboxylate (**A10**)

**Figure 59.** HRMS spectrum of compound **B1.**

(5-phenyl-1,3,4-oxadiazol-2-yl)methyl (4aR,5R,6aS,6bR,8aR,9S,10S,12aR,12bR,14bS)-9-formyl-5,10-dihydroxy-2,2,6a,6b,9,12a-hexamethyl-1,3,4,5,6,6a,6b,7,8,8a,9,10,11,12,12a,12b,13,14b-octadecahydropicene-4a(2H)-carboxylate (**B1**)

**Figure 60.** HRMS spectrum of compound **B2.**

(5-(4-fluorophenyl)-1,3,4-oxadiazol-2-yl)methyl (4aR,5R,6aS,6bR,8aR,9S,10S,12aR,12bR,14bS)-9-formyl-5,10-dihydroxy-2,2,6a,6b,9,12a-hexamethyl-1,3,4,5,6,6a,6b,7,8,8a,9,10,11,12,12a,12b,13,14b-octadecahydropicene-4a(2H)-carboxylate (**B2**)

**Figure 61.** HRMS spectrum of compound **B3.**

(5-(4-chlorophenyl)-1,3,4-oxadiazol-2-yl)methyl (4aR,5R,6aS,6bR,8aR,9S,10S,12aR,12bR,14bS)-9-formyl-5,10-dihydroxy-2,2,6a,6b,9,12a-hexamethyl-1,3,4,5,6,6a,6b,7,8,8a,9,10,11,12,12a,12b,13,14b-octadecahydropicene-4a(2H)-carboxylate (**B3**)

**Figure 62.** HRMS spectrum of compound **B4.**

(5-(4-methoxyphenyl)-1,3,4-oxadiazol-2-yl)methyl (4aR,5R,6aS,6bR,8aR,9S,10S,12aR,12bR,14bS)-9-formyl-5,10-dihydroxy-2,2,6a,6b,9,12a-hexamethyl-1,3,4,5,6,6a,6b,7,8,8a,9,10,11,12,12a,12b,13,14b-octadecahydropicene-4a(2H)-carboxylate (**B4**)

**Figure 63.** HRMS spectrum of compound **B5.**

(5-(3,4,5-trimethoxyphenyl)-1,3,4-oxadiazol-2-yl)methyl (4aR,5R,6aS,6bR,8aR,9S,10S,12aR,12bR,14bS)-9-formyl-5,10-dihydroxy-2,2,6a,6b,9,12a-hexamethyl-1,3,4,5,6,6a,6b,7,8,8a,9,10,11,12,12a,12b,13,14b-octadecahydropicene-4a(2H)-carboxylate (**B5**)

**Figure 64.** HRMS spectrum of compound **B6.**

(5-(4-nitrophenyl)-1,3,4-oxadiazol-2-yl)methyl (4aR,5R,6aS,6bR,8aR,9S,10S,12aR,12bR,14bS)-9-formyl-5,10-dihydroxy-2,2,6a,6b,9,12a-hexamethyl-1,3,4,5,6,6a,6b,7,8,8a,9,10,11,12,12a,12b,13,14b-octadecahydropicene-4a(2H)-carboxylate (**B6**)

**Figure 65.** HRMS spectrum of compound **C1.**

2-(5-oxo-4-phenyl-4,5-dihydro-1H-1,2,4-triazol-1-yl)ethyl (4aR,5R,6aS,6bR,8aR,9S,10S,12aR,12bR,14bS)-9-formyl-5,10-dihydroxy-2,2,6a,6b,9,12a-hexamethyl-1,3,4,5,6,6a,6b,7,8,8a,9,10,11,12,12a,12b,13,14b-octadecahydropicene-4a(2H)-carboxylate (**C1**)

**Figure 66.** HRMS spectrum of compound **C2.**

2-(4-(4-fluorophenyl)-5-oxo-4,5-dihydro-1H-1,2,4-triazol-1-yl)ethyl (4aR,5R,6aS,6bR,8aR,9S,10S,12aR,12bR,14bS)-9-formyl-5,10-dihydroxy-2,2,6a,6b,9,12a-hexamethyl-1,3,4,5,6,6a,6b,7,8,8a,9,10,11,12,12a,12b,13,14b-octadecahydropicene-4a(2H)-carboxylate (**C2**)

**Figure 67.** HRMS spectrum of compound **C3.**

2-(4-(4-chlorophenyl)-5-oxo-4,5-dihydro-1H-1,2,4-triazol-1-yl)ethyl (4aR,5R,6aS,6bR,8aR,9S,10S,12aR,12bR,14bS)-9-formyl-5,10-dihydroxy-2,2,6a,6b,9,12a-hexamethyl-1,3,4,5,6,6a,6b,7,8,8a,9,10,11,12,12a,12b,13,14b-octadecahydropicene-4a(2H)-carboxylate (**C3**)

**Figure 68.** HRMS spectrum of compound **C4.**

2-(4-(3,4-dichlorophenyl)-5-oxo-4,5-dihydro-1H-1,2,4-triazol-1-yl)ethyl (4aR,5R,6aS,6bR,8aR,9S,10S,12aR,12bR,14bS)-9-formyl-5,10-dihydroxy-2,2,6a,6b,9,12a-hexamethyl-1,3,4,5,6,6a,6b,7,8,8a,9,10,11,12,12a,12b,13,14b-octadecahydropicene-4a(2H)-carboxylate (**C4**)

**Figure 69.** HRMS spectrum of compound **C5.**

2-(4-(4-methoxyphenyl)-5-oxo-4,5-dihydro-1H-1,2,4-triazol-1-yl)ethyl (4aR,5R,6aS,6bR,8aR,9S,10S,12aR,12bR,14bS)-9-formyl-5,10-dihydroxy-2,2,6a,6b,9,12a-hexamethyl-1,3,4,5,6,6a,6b,7,8,8a,9,10,11,12,12a,12b,13,14b-octadecahydropicene-4a(2H)-carboxylate (**C5**)

**Figure 70.** HRMS spectrum of compound **C6.**

2-(5-oxo-4-(3,4,5-trimethoxyphenyl)-4,5-dihydro-1H-1,2,4-triazol-1-yl)ethyl (4aR,5R,6aS,6bR,8aR,9S,10S,12aR,12bR,14bS)-9-formyl-5,10-dihydroxy-2,2,6a,6b,9,12a-hexamethyl-1,3,4,5,6,6a,6b,7,8,8a,9,10,11,12,12a,12b,13,14b-octadecahydropicene-4a(2H)-carboxylate (**C6**)

**Figure 71.** HRMS spectrum of compound **D.**

(3-phenylisoxazol-5-yl)methyl (4aR,5R,6aS,6bR,8aR,9S,10S,12aR,12bR,14bS)-9-formyl-5,10-dihydroxy-2,2,6a,6b,9,12a-hexamethyl-1,3,4,5,6,6a,6b,7,8,8a,9,10,11,12,12a,12b,13,14b-octadecahydropicene-4a(2H)-carboxylate (**D**)

**Figure 72.** HRMS spectrum of compound **E.**

2-(5-phenyl-1H-tetrazol-1-yl)ethyl (4aR,5R,6aS,6bR,8aR,9S,10S,12aR,12bR,14bS)-9-formyl-5,10-dihydroxy-2,2,6a,6b,9,12a-hexamethyl-1,3,4,5,6,6a,6b,7,8,8a,9,10,11,12,12a,12b,13,14b-octadecahydropicene-4a(2H)-carboxylate (**E**)


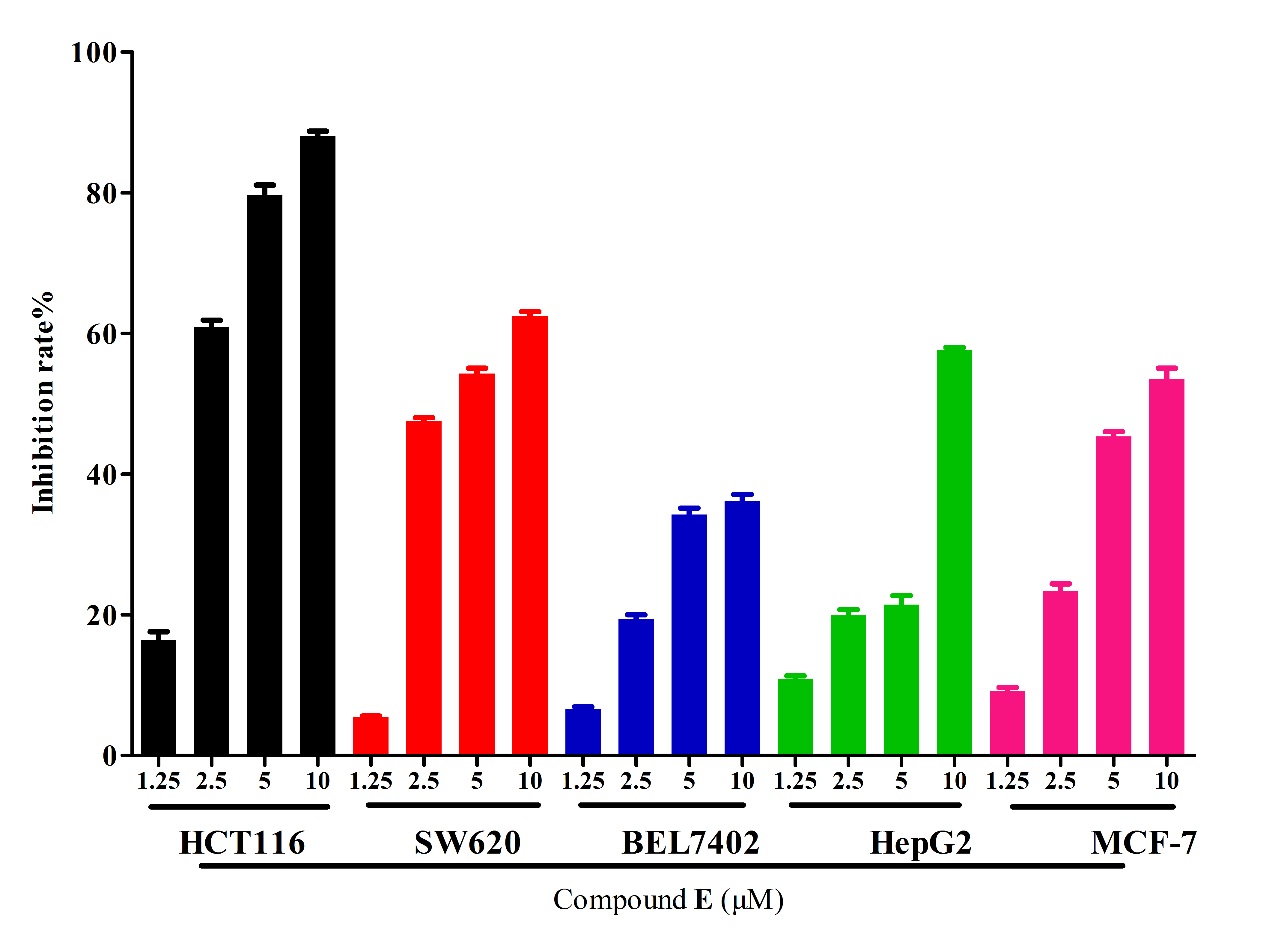


**Figure 73.** The inhibition rate of compound **E** on different tumor cells (HCT116, SW620, BEL7402, HepG2, MCF-7) at different concentrations (1.25, 2.5, 5, 10 μM).

**Figure 74.** HPLC spectrum of compound **A1.**

(1-phenyl-1H-1,2,3-triazol-4-yl)methyl (4aR,5R,6aS,6bR,8aR,9S,10S,12aR,12bR,14bS)-9-formyl-5,10-dihydroxy-2,2,6a,6b,9,12a-hexamethyl-1,3,4,5,6,6a,6b,7,8,8a,9,10,11,12,12a,12b,13,14b-octadecahydropicene-4a(2H)-carboxylate (**A1**)

**Figure 75.** HPLC spectrum of compound **A2.**

(1-(4-fluorophenyl)-1H-1,2,3-triazol-4-yl)methyl (4aR,5R,6aS,6bR,8aR,9S,10S,12aR,12bR,14bS)-9-formyl-5,10-dihydroxy-2,2,6a,6b,9,12a-hexamethyl-1,3,4,5,6,6a,6b,7,8,8a,9,10,11,12,12a,12b,13,14b-octadecahydropicene-4a(2H)-carboxylate (**A2**)

**Figure 76.** HPLC spectrum of compound **B2.**

(5-(4-fluorophenyl)-1,3,4-oxadiazol-2-yl)methyl (4aR,5R,6aS,6bR,8aR,9S,10S,12aR,12bR,14bS)-9-formyl-5,10-dihydroxy-2,2,6a,6b,9,12a-hexamethyl-1,3,4,5,6,6a,6b,7,8,8a,9,10,11,12,12a,12b,13,14b-octadecahydropicene-4a(2H)-carboxylate (**B2**)

**Figure 77.** HPLC spectrum of compound **B3.**

(5-(4-chlorophenyl)-1,3,4-oxadiazol-2-yl)methyl (4aR,5R,6aS,6bR,8aR,9S,10S,12aR,12bR,14bS)-9-formyl-5,10-dihydroxy-2,2,6a,6b,9,12a-hexamethyl-1,3,4,5,6,6a,6b,7,8,8a,9,10,11,12,12a,12b,13,14b-octadecahydropicene-4a(2H)-carboxylate (**B3**)

**Figure 78.** HPLC spectrum of compound **B4.**

(5-(4-methoxyphenyl)-1,3,4-oxadiazol-2-yl)methyl (4aR,5R,6aS,6bR,8aR,9S,10S,12aR,12bR,14bS)-9-formyl-5,10-dihydroxy-2,2,6a,6b,9,12a-hexamethyl-1,3,4,5,6,6a,6b,7,8,8a,9,10,11,12,12a,12b,13,14b-octadecahydropicene-4a(2H)-carboxylate (**B4**)

**Figure 79.** HPLC spectrum of compound **B5.**

(5-(3,4,5-trimethoxyphenyl)-1,3,4-oxadiazol-2-yl)methyl (4aR,5R,6aS,6bR,8aR,9S,10S,12aR,12bR,14bS)-9-formyl-5,10-dihydroxy-2,2,6a,6b,9,12a-hexamethyl-1,3,4,5,6,6a,6b,7,8,8a,9,10,11,12,12a,12b,13,14b-octadecahydropicene-4a(2H)-carboxylate (**B5**)

**Figure 80.** HPLC spectrum of compound **B6.**

(5-(4-nitrophenyl)-1,3,4-oxadiazol-2-yl)methyl (4aR,5R,6aS,6bR,8aR,9S,10S,12aR,12bR,14bS)-9-formyl-5,10-dihydroxy-2,2,6a,6b,9,12a-hexamethyl-1,3,4,5,6,6a,6b,7,8,8a,9,10,11,12,12a,12b,13,14b-octadecahydropicene-4a(2H)-carboxylate (**B6**)

**Figure 81.** HPLC spectrum of compound **C1.**

2-(5-oxo-4-phenyl-4,5-dihydro-1H-1,2,4-triazol-1-yl)ethyl (4aR,5R,6aS,6bR,8aR,9S,10S,12aR,12bR,14bS)-9-formyl-5,10-dihydroxy-2,2,6a,6b,9,12a-hexamethyl-1,3,4,5,6,6a,6b,7,8,8a,9,10,11,12,12a,12b,13,14b-octadecahydropicene-4a(2H)-carboxylate (**C1**)

**Figure 82.** HPLC spectrum of compound **C3.**

2-(4-(4-chlorophenyl)-5-oxo-4,5-dihydro-1H-1,2,4-triazol-1-yl)ethyl (4aR,5R,6aS,6bR,8aR,9S,10S,12aR,12bR,14bS)-9-formyl-5,10-dihydroxy-2,2,6a,6b,9,12a-hexamethyl-1,3,4,5,6,6a,6b,7,8,8a,9,10,11,12,12a,12b,13,14b-octadecahydropicene-4a(2H)-carboxylate (**C3**)

**Figure 83.** HPLC spectrum of compound **C5.**

2-(4-(4-methoxyphenyl)-5-oxo-4,5-dihydro-1H-1,2,4-triazol-1-yl)ethyl (4aR,5R,6aS,6bR,8aR,9S,10S,12aR,12bR,14bS)-9-formyl-5,10-dihydroxy-2,2,6a,6b,9,12a-hexamethyl-1,3,4,5,6,6a,6b,7,8,8a,9,10,11,12,12a,12b,13,14b-octadecahydropicene-4a(2H)-carboxylate (**C5**)

**Figure 84.** HPLC spectrum of compound **C6.**

2-(5-oxo-4-(3,4,5-trimethoxyphenyl)-4,5-dihydro-1H-1,2,4-triazol-1-yl)ethyl (4aR,5R,6aS,6bR,8aR,9S,10S,12aR,12bR,14bS)-9-formyl-5,10-dihydroxy-2,2,6a,6b,9,12a-hexamethyl-1,3,4,5,6,6a,6b,7,8,8a,9,10,11,12,12a,12b,13,14b-octadecahydropicene-4a(2H)-carboxylate (**C6**)

**Figure 85.** HPLC spectrum of compound **E.**

2-(5-phenyl-1H-tetrazol-1-yl)ethyl (4aR,5R,6aS,6bR,8aR,9S,10S,12aR,12bR,14bS)-9-formyl-5,10-dihydroxy-2,2,6a,6b,9,12a-hexamethyl-1,3,4,5,6,6a,6b,7,8,8a,9,10,11,12,12a,12b,13,14b-octadecahydropicene-4a(2H)-carboxylate (**E**)
